# Gene-based microbiome representation enhances host phenotype classification

**DOI:** 10.1128/msystems.00531-23

**Published:** 2023-07-05

**Authors:** Thomas Deschênes, Fred Wilfried Elom Tohoundjona, Pier-Luc Plante, Vincenzo Di Marzo, Frédéric Raymond

**Affiliations:** 1 Centre Nutrition, Santé et Société (NUTRISS) – Institut sur la Nutrition et les Aliments Fonctionnels (INAF), Université Laval, Québec, Canada; 2 Canada Research Excellence Chair on the Microbiome-Endocannabinoidome Axis in Metabolic Health (CERC-MEND), Quebec City, Quebec, Canada; 3 Institut Intelligence et Données, Université Laval, Québec, Canada; 4 École de nutrition, Faculté des sciences de l’agriculture et de l’alimentation (FSAA), Université Laval, Québec, Canada; 5 Centre de recherche de l’Institut universitaire de cardiologie et de pneumologie de Québec (IUCPQ), Québec, Canada; 6 Département de médecine, Faculté de Médecine, Université Laval, Québec, Canada; 7 Joint International Unit on Chemical and Biomolecular Research on the Microbiome and its Impact on Metabolic Health and Nutrition (UMI-MicroMeNu), Quebec City, Canada; University of California San Diego, La Jolla, California, USA

**Keywords:** microbiome, machine learning, metagenomics, shotgun microbiome, feature selection, gene clusters, interpretable models, metabolic health, gut-brain axis, endocannabinoidome

## Abstract

With the concomitant advances in both the microbiome and machine learning fields, the gut microbiome has become of great interest for the potential discovery of biomarkers to be used in the classification of the host health status. Shotgun metagenomics data derived from the human microbiome is composed of a high-dimensional set of microbial features. The use of such complex data for the modeling of host-microbiome interactions remains a challenge as retaining *de novo* content yields a highly granular set of microbial features. In this study, we compared the prediction performances of machine learning approaches according to different types of data representations derived from shotgun metagenomics. These representations include commonly used taxonomic and functional profiles and the more granular gene cluster approach. For the five case-control datasets used in this study (Type 2 diabetes, obesity, liver cirrhosis, colorectal cancer, and inflammatory bowel disease), gene-based approaches, whether used alone or in combination with reference-based data types, allowed improved or similar classification performances as the taxonomic and functional profiles. In addition, we show that using subsets of gene families from specific functional categories of genes highlight the importance of these functions on the host phenotype. This study demonstrates that both reference-free microbiome representations and curated metagenomic annotations can provide relevant representations for machine learning based on metagenomic data.

**IMPORTANCE:**

Data representation is an essential part of machine learning performance when using metagenomic data. In this work, we show that different microbiome representations provide varied host phenotype classification performance depending on the dataset. In classification tasks, untargeted microbiome gene content can provide similar or improved classification compared to taxonomical profiling. Feature selection based on biological function also improves classification performance for some pathologies. Function-based feature selection combined with interpretable machine learning algorithms can generate new hypotheses that can potentially be assayed mechanistically. This work thus proposes new approaches to represent microbiome data for machine learning that can potentiate the findings associated with metagenomic data.

## INTRODUCTION

Associations between microbiome-derived features and human diseases have been extensively documented in recent years, especially in chronic diseases (1 - 5). Numerous studies have applied machine learning (ML) on a wide range of these microbiome datasets to harvest hidden knowledge and better understand the implication of the gut microbiome in health and diseases (6 - 11). In the context of the inference of host phenotype for disease classification, such modeling can be used to point toward important microbial biomarkers or serve as potential diagnostic tools.

High-throughput sequencing technologies permit the deep characterization of microbiomes, including non-cultivable microbes (12). However, metagenomic data need to be processed to allow their association with the host phenotype. Shotgun metagenomics studies often use reference-based data types like taxonomic and functional profiles (6). For example, the MetaPhlAn software computes taxonomic composition from metagenomic data, an output similar to 16S metataxonomic analysis (13). HUMAnN, instead, provides functional annotation of metagenomes on known metabolic pathways (13). Maybe the greatest challenge in building the functional profiles of microbial communities is the difficulty to associate genes with specific functions. Indeed, 39.6% of the comprehensive Integrated Gene Catalog (IGC) of the human gut microbiome was unmapped to functional databases, and 15–20% of the remaining has been observed before but with no known function (14). The use of metagenome *de novo* assembly can help overcome these limitations as the information encoded in assemblies is not limited to the content of databases.

Several machine learning algorithms have been used to classify the host phenotype from microbiome data, ranging from random forest (RF) to deep learning-based approaches (15, 16). Databases have been built to simplify the access to microbiome data for machine learning purposes (17). Most studies use taxonomic profiling as input data for prediction, either based on 16S metataxonomic data or taxonomic profiling from shotgun data (18 - 20). Using publicly available 16S metataxonomic data, Giliberti and collaborators have observed that classification of host phenotypes based on microbiome data is mainly driven by the presence or absence of microbial taxa (21). Multi-omics approaches combining microbiome profiling with metabolomics (15) or host-related variables (22) are also being increasingly published. Lee and Rho have used deep neural networks to obtain an embedded representation that successfully combined genome-level data with taxonomic and functional profiles, providing improved classification (23). Multiclassification strategies, where several diseases are classified using a single algorithm, have also been proposed (24). Ecosystem-inspired machine learning approaches have shown promising results (25). An interesting example of such multi-omics approaches was the prediction of glycemic response to food from microbiome and clinical data (26). The available literature on the use of metagenomic gene content for machine learning classification remains marginal although it does suggest that such an approach is a promising avenue. Indeed, the work by Le Goallec and collaborators suggests that using gene content for phenotype prediction could outperform taxonomic profiling (7). Machine learning has also been used to identify biomarkers of Type 2 diabetes (T2D) from microbiome genes annotated with eggNOG or KEGG databases (27).

**Fig 1:**
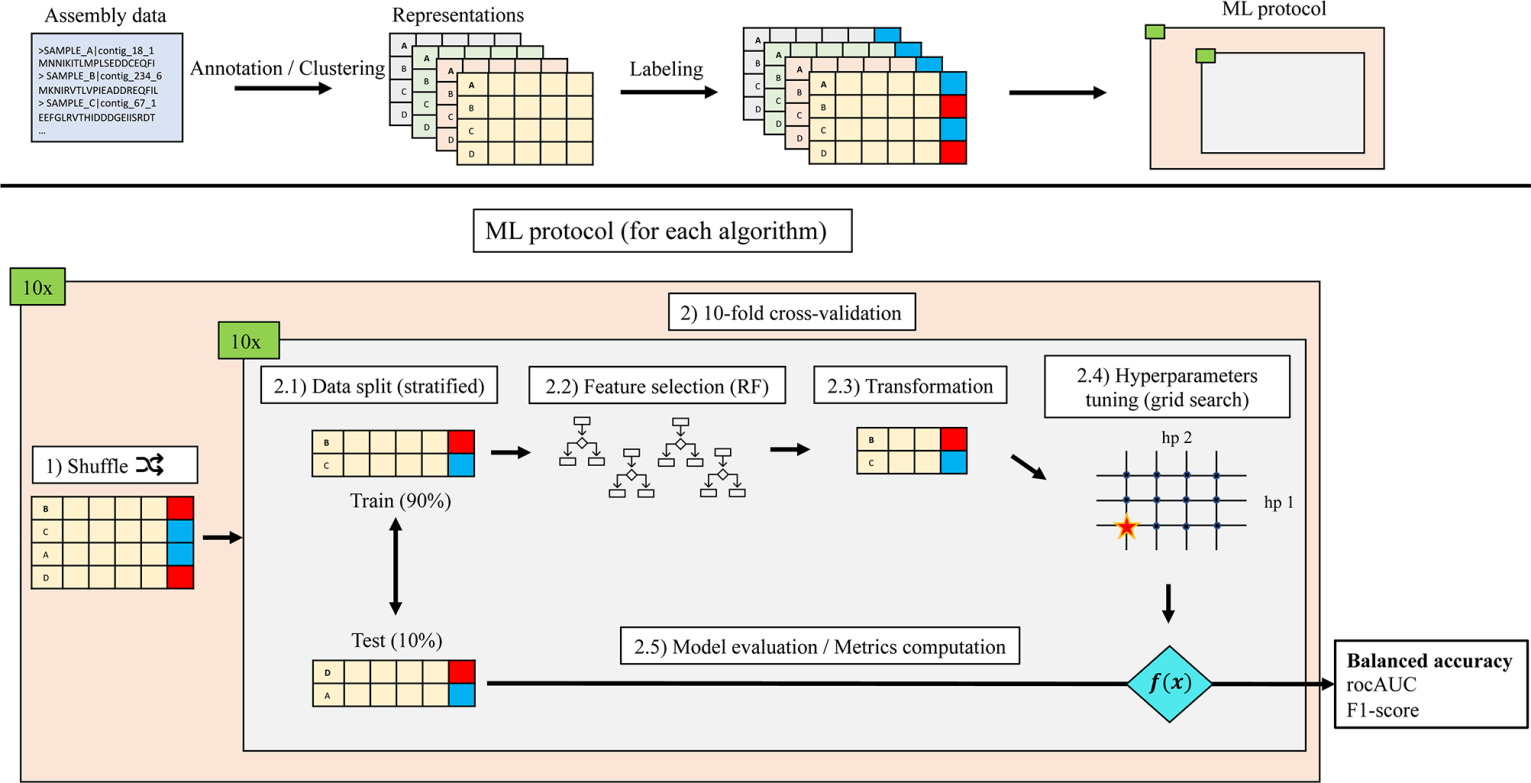
Schematic representation of bioinformatics and machine learning protocols. Each representation was given as input to the machine learning (ML) protocol. (1) Each 10 repeats of the 10-fold cross-validation was initiated with a different random seed. To limit randomization bias, the examples in each split were the same for all representations. (2.1) For each split, the train and test sets were stratified according to class proportions. (2.2) A random forest (RF) was applied to select important features. (2.3) Only selected features were kept for the next steps. (2.4) For each algorithm, an array of hyperparameters (hps) was comprehensively tested and the combination yielding the best balanced accuracy was kept. Other metrics were also computed: area under the receiver operating characteristic curve (rocAUC) and F1-score.

In this work, we compared disease classification performances using different data representation strategies based on shotgun metagenomics. Our hypothesis was that a granular, gene-based microbiome representation could provide improved classification and interpretability compared to taxonomical profiling. In addition to total microbiome gene content, we also used curated subsets of gene families, based on existing databases, and hypothesis-driven selection based on molecular function. We selected five human microbiome datasets of shotgun metagenomics from case-control studies with publicly available raw data. The five datasets included in our study were from Type 2 diabetes (T2D, *n* = 199) (2), obesity (OB, *n* = 265) (1), liver cirrhosis (LC, *n* = 237) (4), colorectal cancer (CRC, *n* = 141) (5), and inflammatory bowel disease (IBD, *n* = 124) studies (3). For every dataset, we evaluated the effect of data representation in a prediction task consisting of a binary classification of healthy and diseased individuals. The data representations used in our study were the taxonomic profile (MetaPhlAn3) (13), the potential metabolic functional profile (HUMAnN3) (13), clusters of genes at 70% identity, and different subsets of gene clusters based on reference databases or hypothesis-driven protein domain selection. For every data type, we assessed the predictive performance of nine classifiers: two support vector machines (SVMs) with linear kernels (L1- and L2-regularized), a SVM with a radial basis function kernel (SVM-rbf), two logistic regressions (LRs) (L1- and L2-regularized), a decision tree (DT), a random forest (RF), a set covering machine (SCM) (28), and a random set covering machine (rSCM) (29), an ensemble algorithm derived from the SCM. For every dataset, we applied a feature-selection random forest prior to the machine learning protocol. We evaluated the final models with different metrics, notably the balanced accuracy to account for imbalanced classes, and the popular area under the receiver operating characteristic curve (rocAUC). Figure 1 summarizes our experimental protocol. Our study demonstrates that gene families, used either as reference-free microbiome representations or as curated metagenomic annotations, hold the potential to contribute to a better understanding of host-microbiome biology.

## RESULTS

### Gene content is an alternative to microbiome taxonomic and functional profiles

**Fig 2:**
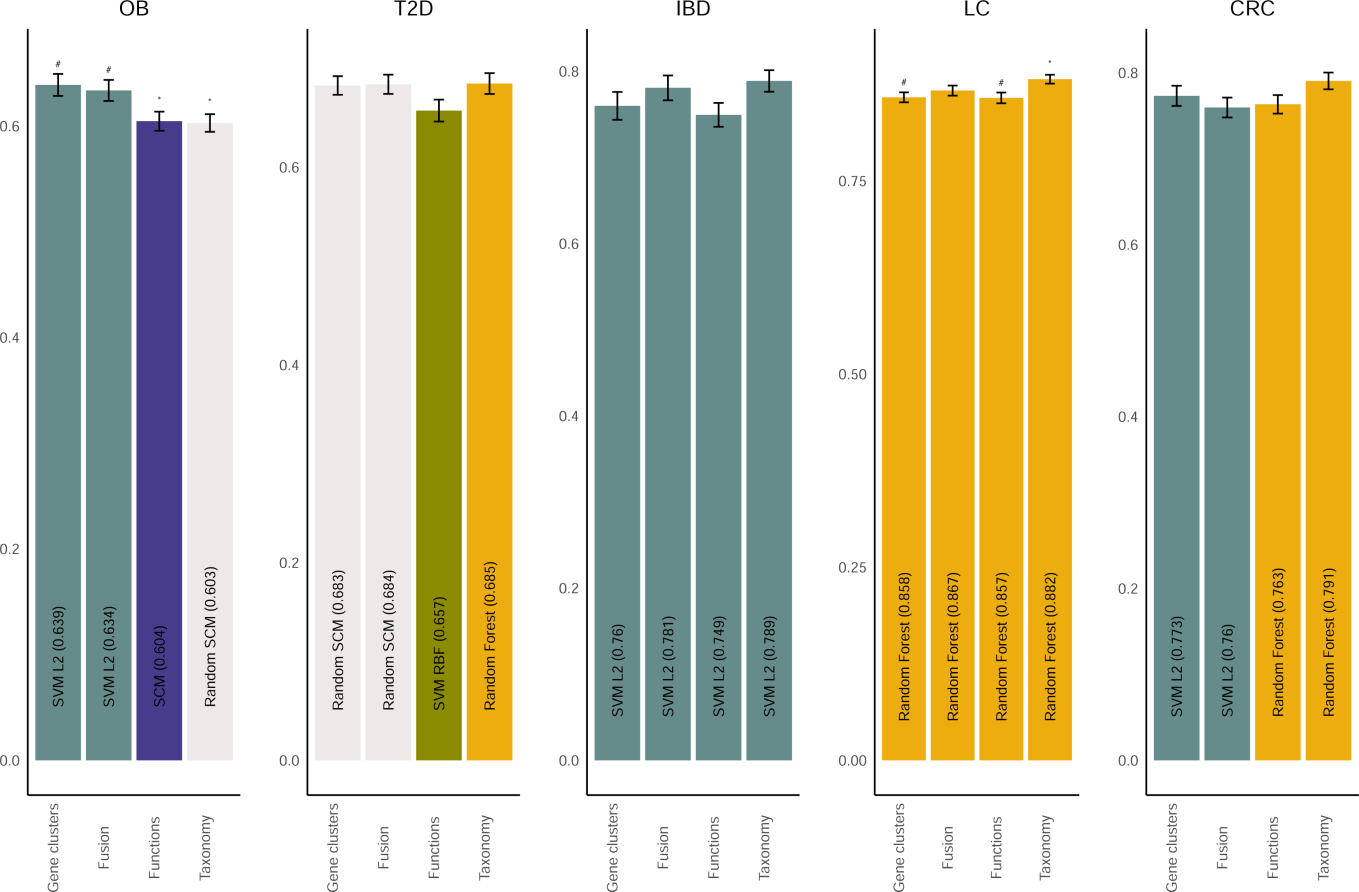
Metagenomic data representation method influences machine learning classification results in a dataset-dependent manner. Comparison of the performance of gene clusters, gene functions, taxonomy, and fusion of the three representations for case/control classification of colorectal cancer (CRC), inflammatory bowel diseases (IBD), liver cirrhosis (LC), obesity (OB) and Type 2 diabetes (T2D). Performance was assessed using balanced accuracy. For each data representation, the average balanced accuracy of the best performing algorithm is shown with error bars representing 95% confidence intervals. The name of the best performing algorithm for each representation is shown within the bars. Symbols above bars indicate that the balanced accuracy of data representation is significantly different after false discovery rate (FDR) correction (*P* < 0.05) compared to taxonomy (#) or gene clusters (*).

Our first objective was to determine if gene cluster abundances performed better, similar, or worse than taxonomical abundances in machine learning problems aiming to classify health/disease from shotgun metagenomics data. To do so, we compared, for each dataset, the performance of data representations and machine learning algorithms using a generalized linear model (GLM) with random individual effects based on sample splits. We compared the balanced accuracy obtained using gene clusters and taxonomical relative abundances for nine different machine learning algorithms (Fig. 2 for best algorithms and Supplementary Figure S1 to S5 for all algorithms). The performance of microbiome representations generally depended on the algorithm used for machine learning, and the best performing combination was generally different among datasets. Results for F1 score and AUC are shown in Supplementary Figure S6 to S15, respectively. When considering the best performing algorithm for each representation, gene clusters had significantly higher balanced accuracy than taxonomy for OB, were not significantly different from taxonomy for T2D, IBD, and CRC, and were significantly lower than taxonomy for LC (Fig. 2). These results indicate that gene clusters encode the relevant information for disease prediction in a way that is similar to taxonomy, although for some problem/algorithm combinations one representation is better than the other.

We also investigated how gene clusters and taxonomy compared to functional profiling of microbiome determined using the HuMANN3 software (identified as Functions in Fig. 2). Gene clusters balanced accuracy was significantly higher than HuMANN3 functions in OB only. Taxonomy was significantly higher than HuMANN3 functions in LC only. Learning using a combination of gene clusters, taxonomy, and functions (identified as Fusion in Fig. 2) showed a signal significantly higher than taxonomy in OB only. Despite the high dimensionality of the gene clusters, the features selected with the initial random forest generally allowed classification performances comparable to taxonomical abundances or functional profiling across the five datasets.

### COG classification favors interpretability in phenotype prediction

**Fig 3:**
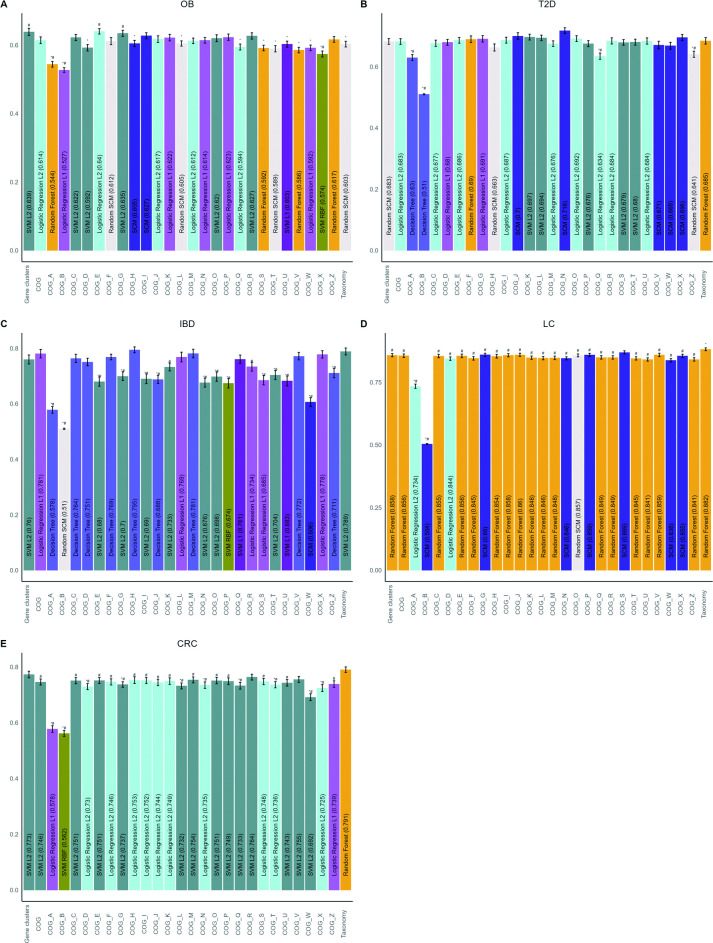
COG families’ gene clusters improve classification performance for obesity and Type 2 diabetes. Comparison of the performance of gene clusters filtered for similarity with genes from COG families affects case/control classification of (**A**) obesity (OB), (**B**) Type 2 diabetes (T2D), (**C**) inflammatory bowel diseases (IBD), (**D**) liver cirrhosis (LC), and (**E**) colorectal cancer (CRC). Performance was assessed using balanced accuracy. For each data representation, the average balanced accuracy of the best performing algorithm is shown with error bars representing 95% confidence intervals. The name of the best performing algorithm for each representation is shown within the bars. Symbols above bars indicate that the balanced accuracy of data representation is significantly different after FDR (*P* < 0.05) compared to taxonomy (#) or gene clusters (*).

We investigated the classification potential of gene clusters selected based on the COG database, which allows to classify bacterial genes in 26 broad function categories (Fig. 3 for best algorithms, Supplementary Figure S16 to S20 for all algorithms, Supplementary Figure S21 to S25 for F1 score, and Supplementary Figure S26 to S30 for AUC). Following the previous experiment, we hypothesized that, for specific diseases, some COG categories could allow improved disease phenotype prediction, as specific gene functions could be associated with the phenotype. In addition, the use of smaller feature sets could importantly reduce the resources needed for model building. We thus assigned gene clusters to COG categories based on sequence similarity and used the same ML protocol as before for each new subset of gene clusters. For colorectal cancer and liver cirrhosis, gene clusters filtered by COG categories yielded significantly lower balanced accuracy than taxonomy. In contrast, for obesity phenotype prediction, two COGs had balanced accuracy significantly higher than that of taxonomy, namely, amino acid transport and metabolism (E) and carbohydrate transport and metabolism (G). In IBD phenotype prediction, co-enzyme transport and metabolism (H) provided the highest balanced accuracy, although this difference was not statistically significant compared to gene clusters and taxonomy. Finally, for Type 2 diabetes, the COG category cell motility (*N*) had higher balanced accuracy than taxonomy and all gene clusters but was not statistically significant. This model was generated using the SCM algorithm, which provides sparse interpretable models. In this case, the flagellar protein export ATPase FliI from *Roseburia* and *Lachnospira*, both belonging to the family *Lachnospiraceae,* were associated with the healthy condition. As was the prepilin-type N-terminal cleavage/methylation domain-containing protein from *Phascolarctobacterium faecium*. Overall, these results suggest that functional classification of gene clusters can provide, in certain cases, improved prediction compared to common methods, in addition to favoring interpretability.

### Database-derived feature selection

**Fig 4:**
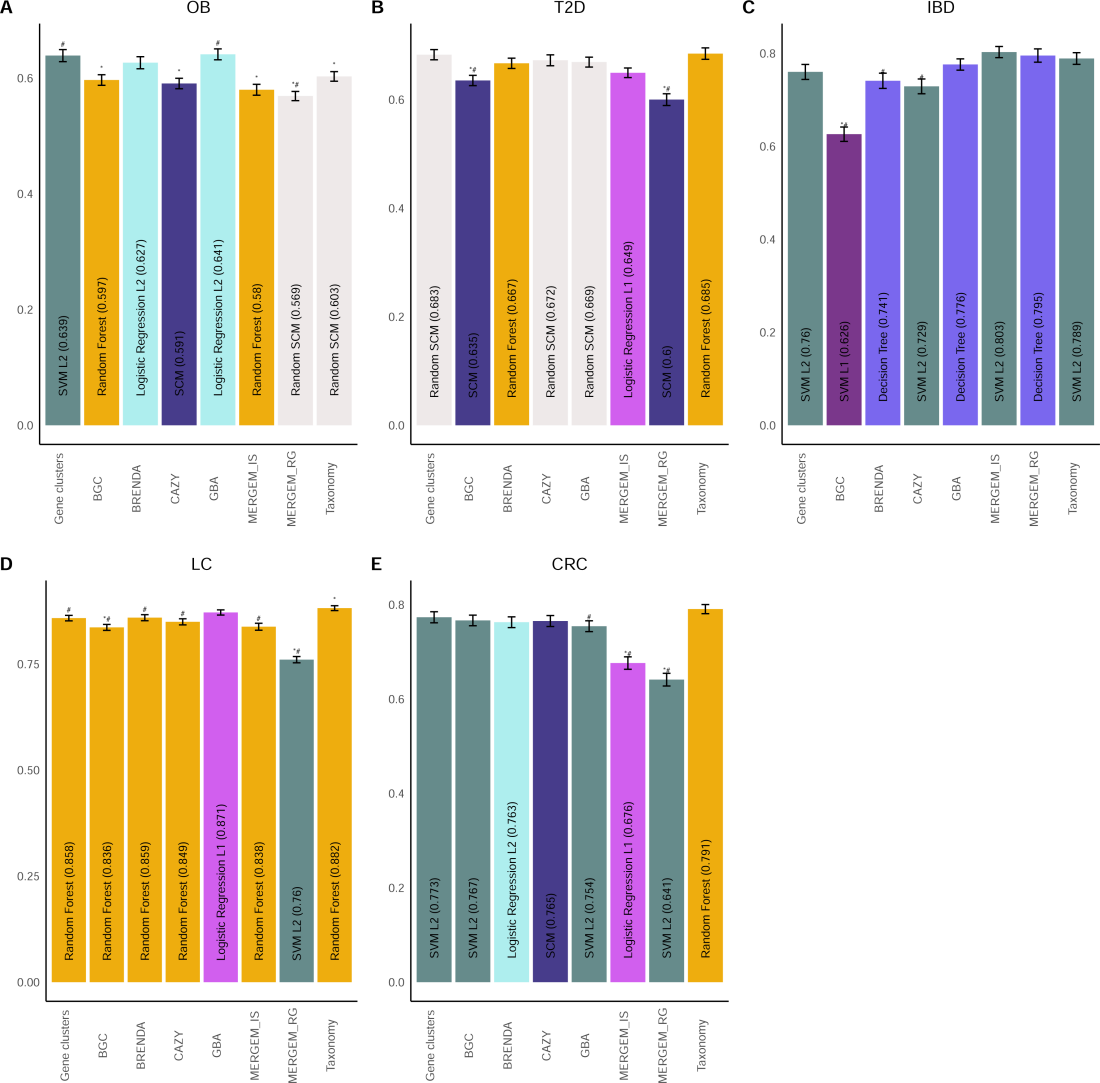
Subsets of gene families derived from function-based databases affect classification performance. Comparison of the performance of gene clusters filtered for similarity with genes from datasets affects case/control classification of (**A**) obesity (OB), (**B**) Type 2 diabetes (T2D), (**C**) inflammatory bowel diseases (IBD), (**D**) liver cirrhosis (LC), and (**E**) colorectal cancer (CRC). Performance was assessed using balanced accuracy. For each data representation, the average balanced accuracy of the best performing algorithm is shown with error bars representing 95% confidence intervals. The name of the best performing algorithm for each representation is shown within the bars. Symbols above bars indicate that the balanced accuracy of data representation is significantly different after FDR (*P* < 0.05) compared to taxonomy (#) or gene clusters (*).

To investigate the use of feature selection based on specific biological properties of the genes, we tested our protocol on specific groups of gene clusters selected based on specific biological functions. Each group consisted of gene clusters that contained at least one sequence with similarities to known genes with confirmed function. The databases used in our protocol were from specific functional categories of genes: enzymes implicated in the use of carbohydrates [Carbohydrate-Active Enzymes (CAZy) database (30)], genes of antibiotic resistance [Mobile Elements and Resistance Genes Enhanced for Metagenomics (MERGEM) database (31)], insertion sequences (MERGEM), biosynthetic gene clusters [Minimum Information about a Biosynthetic Gene cluster (MIBiG) database (32)], and enzymes from the comprehensive enzymes database BRENDA (33). Representations based on subsets of gene clusters did not significantly outperform using all gene clusters for the five datasets (Fig. 4 for best algorithms, Supplementary Figure S31 to S35 for all algorithms, Supplementary Figure S36 to S40 for F1 score, and Supplementary Figure S41 to S45 for AUC). However, in the IBD dataset, insertion sequences and resistance genes from the MERGEM database showed higher balanced accuracy than both gene clusters and taxonomy, although the difference was not statistically significant. While the performance of some subsets of gene clusters did not generally outperform gene clusters or taxonomy-based predictions, we suggest that domain knowledge is a potential tool to be applied for feature selection for the prediction of phenotypes using metagenomic gene content.

### Hypothesis-driven feature selection for project-specific interpretability

**Fig 5:**
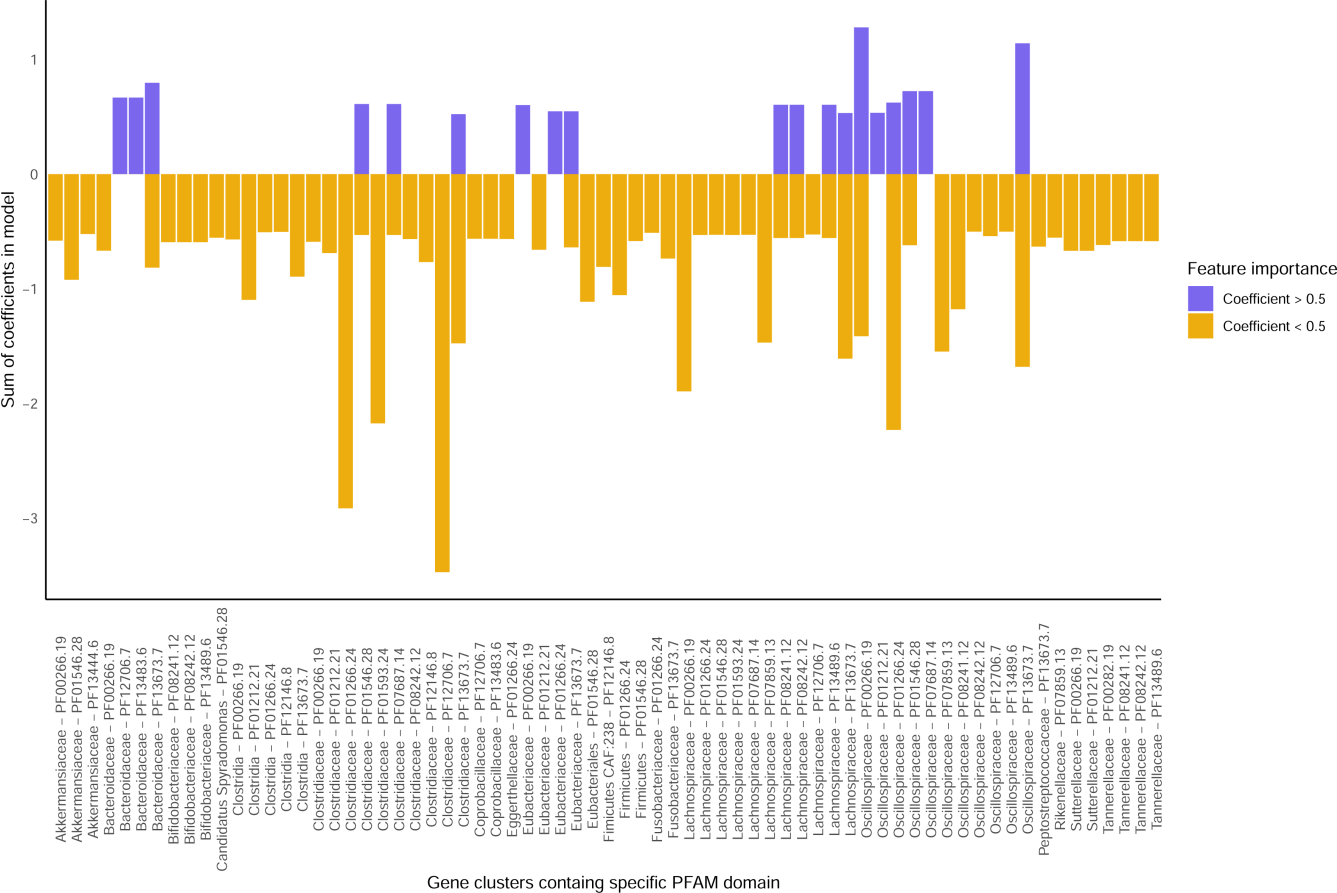
Most important features to classify obesity phenotype using L2-regularized logistic regression on gene clusters containing protein domains potentially associated with the production of gut-brain axis or endocannabinoid-like molecules. Gene clusters were grouped based on their putative taxonomical origin at the family rank, or higher if not possible to classify at the family, and the Pfam domains associated with GBA that they contained. The sum of the positive coefficients (purple) or negative coefficients (orange) is shown in the barplot. Only gene clusters with absolute values greater than 0.5 in the model are shown.

As a proof of concept, we designed an experiment with gene cluster selection based on molecular functions related to a specific hypothesis. In this regard, we hypothesized that microbial genes containing Pfam (34) domains from enzymes implicated in the production and degradation of endocannabinoid analogs or of potential gut-brain axis effectors could encode relevant information about the crosstalk between the gut microbiome and metabolic diseases (35 - 37). Interestingly, genes containing gut-brain axis (GBA) associated domains permitted the highest balanced accuracy for the prediction of obesity compared to the other representations (Fig. 4, identified as GBA). Indeed, the GBA subset of genes significantly outperformed taxonomy for the OB dataset and had higher balanced accuracy than gene clusters, but this difference was not statistically significant. This model was obtained using L2-regularized data with the logistic regression algorithm. Since the subset of gene clusters used to build this model was associated with a specific hypothesis, we investigated the features that were important in the generated models and interpreted them in view of current knowledge. Thus, we summed the coefficients of the 100 models generated for all the features that were included in at least one model, providing a consolidated list of the features that were used for prediction. We investigated the gene clusters that had summed coefficients greater than 0.5 or lower than −0.5 (Fig. 5). In this model, 14 gene clusters had values >0.5 while 68 had values lower than −0.5. A majority of gene clusters were similar to genes found in the class *Clostridia*, specifically *Clostridiaceae, Eubacteriaceae, Lachnospiraceae,* and *Oscillospiraceae. Bacteroidaceae* gene clusters were positive predictors of obesity, while numerous other taxa were negatively associated with obesity, including *Akkermansiaceae, Bifidobacteriaceae,* and *Eggerthellaceae,* which are generally associated with good metabolic health (38, 39). In particular, GNAT family *N*-acetyltransferase domains were the most represented in the gene clusters included in models and were observed mostly in *Bacteroidaceae,* as previously observed (40), and several *Clostridia* families. These enzymes are involved in the synthesis of *N*-acyl amides, including lipoaminoacids and endocannabinoid analogs, which are agonists of G-protein coupled receptors and nuclear (i.e., peroxisome proliferator-activated) receptors playing important roles in physiological and pathological metabolic responses (40). The strongest negative coefficient was observed for the β-lactamase superfamily from *Clostridiaceae*. In humans, metallo-β-lactamases can play a role in bioactive lipid metabolism, as in the case of the enzyme NAPE-PLD, which catalyzes the biosynthesis of the endocannabinoid, anandamide, and other *N*-acyl ethanolamines from the corresponding fatty acids (41). The differential distribution of these domains in *Clostridia* families and species could explain the proposed dual nature of these taxa in metabolic control (42). This proof-of-concept experiment demonstrates the potential of hypothesis-driven data representations for machine learning. In addition, this approach allowed us to investigate a well-studied dataset from a novel perspective and provides insight that might lead to new findings.

## DISCUSSION

In this study, we investigated how different representations of shotgun microbiome data could affect the capacity to classify health and disease phenotypes in five classification tasks. We observed that the best performing representation was not the same for all pathological conditions as represented by the studied datasets, which suggests that there exists no one-size-fits-all data representation approach to predict diseases from shotgun sequencing gut microbiome data. Indeed, the best performing combination of data representations and machine learning algorithms was different among the five problems studied, each represented by a single study. The most frequently used representation for microbiome data, i.e., taxonomical abundance, in this case determined using MetaPhlAn3, showed consistently higher balanced accuracy than all the other representations tested for colorectal cancer and liver cirrhosis, using random forest algorithm. Our curated collection of genes potentially involved in the production of molecules associated with the gut-brain axis provided the best predictive properties for obesity using the L2-regularized logistic regression. For T2D, genes associated with cell motility (COG category N) provided optimal prediction using the SCM algorithm. Finally, genes associated with mobile genetic elements provided the best prediction for IBD using the L2-regularized SVM algorithm. Thus, our main conclusion is that different microbiome representations should be compared to improve phenotype classification based on shotgun microbiome data. We initially hypothesized that the complete gene content from shotgun microbiome data could improve phenotype prediction compared to taxonomic composition, which was validated only in OB classification problems. Gene families have the potential to encode information that is lost when using only taxonomic composition. Since genes with unknown functions are not eliminated from the data, potentially important biological information encoded in these genes might increase the quality of the predictions. Gene clusters may include genes that are representative of species or even strains. They may even allow to account for genes that can be horizontally transferred between species or gene families that may be shared by unrelated taxa.

To improve the interpretability of machine learning models and to reduce the initial feature size of datasets, we filtered the gene families for similarity with gene databases or COG categories. This approach allowed us to screen for the association of specific functions with diseases while retaining feature granularity. A similar filtering approach has previously been used to compare microbial genomes’ k-mer content to determine if specific gene functions could be associated with intraspecies subgroups (43). Here, we did observe improved performance when using specific subgroups of genes to train machine learning models compared to taxonomy, indicating that this approach has potential use in machine learning applied to metagenomic data. Moreover, the computing time and memory required to learn models on these smaller datasets are orders of magnitude lower than using all gene clusters, while providing similar or improved performance. A striking example of this potential was observed for the T2D dataset, where the sparse SCM algorithm allowed to pinpoint genes associated with cell motility. Flagellar protein export ATPase FliI from *Lachnospiraceae* and prepilin-type N-terminal cleavage/methylation domain-containing protein from *Phascolarctobacterium faecium* were identified as potential biomarkers for T2D. Indeed, *Lachnospiraceae* has been associated with T2D in previous studies (2, 44), but the present sparse machine learning model provided information potentially useful to gain better mechanistic insights into this disease based on specific genes from both taxa. Therefore, this approach has the potential to uncover genes associated with disease phenotypes. Predictive performances of gene-level data type in host phenotype prediction might indicate that, for certain diseases, microbial functions, perhaps unsurprisingly, are in a closer relationship with the phenotype than the microbes themselves. In addition, sparse models targeting specific genes would be more amenable to cross-cohort validation and to potential clinical applications (6).

We also used custom gene family selection based on selected Pfam domains to investigate potential associations between diseases and genes potentially involved in the synthesis or degradation of gut-brain axis-related metabolites. The potential synthesis of neuroactive mediators by gut prokaryotes has been shown previously, for example, with dopamine-related metabolites (45). Very recently, commensal gut bacteria have also been shown to produce endocannabinoid analogs which have the potential to interact with the host GPCR signaling system and modulate gut and metabolic function (40). Knowing this, we hypothesized that domains from enzymes implicated in the production and degradation of endocannabinoid-like molecules, such as *N*-acylated amino acids and *N*-acylethanolamines, and neurotransmitters could encode new information about the crosstalk between dysbiosis, the expanded endocannabinoid system, and disease. This could also highlight the potential roles of microbial gut-brain axis effectors in contexts other than psychiatric and neurological disorders. For instance, endocannabinoid congeners and analogs have been shown to be involved in metabolic diseases, intestinal processes, and mental disorders (46, 47), as they can be agonists or antagonists to different receptors involved in these systems. There exist numerous metabolites that can be biosynthesized by the microbiome, and each amino acid-fatty acid combination in the form of amides may provide either a positive or negative impact on host metabolic or mental phenotype, much in the same way as some host-produced *N*-acyl amino acids do (48 - 50). GNAT *N*-acetyltransferase orthologs that were part of the predictor for obesity could be involved in such processes (40, 51 - 54). Although these results remain preliminary, they demonstrate the high potential of a hypothesis-driven approach for microbiome feature selection that could inspire future machine learning approaches and provide new perspectives to investigate existing datasets.

A challenge of using gene families for phenotype prediction is the size of the resulting matrix, which requires important memory and computing to produce models. For example, the machine learning protocol on the T2D task (*n* = 199) took 6:09:01 (h:m:s) and 65.5 gigabytes of memory when using the matrix of gene clusters. Filtering the gene clusters yielded substantially smaller datasets and was therefore less demanding in terms of resources. For cluster matching insertion sequences (MERGEM_IS), the protocol took 1:58:38 and 1.16 gigabytes of memory. In comparison, taxonomic and functional profiles (MetaPhlAn3 and HUMAnN3) took 2:48:06 for 0.223 gigabytes and 06:13:48 for 0.300 gigabytes of memory, respectively. Information about resources needed for other data types can be found in Supplementary Table S1.

An additional risk of high-dimensional data type is overfitting, which is the learning of subject-specific features instead of class-defining features. To deal with this problem, we used an embedded feature selection technique that consisted in training a random forest, ranking the features according to the resulting models, and learning on subsets of these ranked features, a method that has been used previously (6, 11). Clustering co-abundant gene groups could also contribute to efficient feature reduction (55). In the future, other methods for data reduction should be investigated to favor the use of granular metagenomic data, such as gene content or k-mers (56), which allow nucleotide resolution but generate immense feature sizes with more than billions of features.

Another potential challenge of this analysis is the presence of numerous correlated features. Even at the taxonomy level, measuring pairwise correlation can be computationally challenging. As the number of possible two-feature interactions for a dataset with *n* features is [*n ×* (*n −* 1)]/2, this often results in millions of calculations since microbiomes are composed of thousands of microorganisms. Plus, because microbes live in a community, it is likely that there are three-feature, four-feature, or more interactions (57). These problems are exacerbated when using microbiome data at a gene-level granularity, the genes being potentially carried by correlated microbes. It is possible that genes used in prediction models would be proxies for specific taxonomical groups rather than functions directly associated with the predicted phenotype. These elements should be considered in the development of dimensionality reduction techniques for gene-level metagenomics data.

A limitation of this study is the use of a single cohort for each classification problem. This limits the reach of the results, as the best combinations of data representations and learning algorithms might not be transferable to similar classification problems using other datasets. Also, the models interpreted in this work may not be transferable to other datasets. As such, they should be considered as hypotheses that should be validated in future works. In all cases, model interpretation should be followed by experimental validation using external cohorts. Nonetheless, using these five classification problems, well studied in the literature, allowed us to demonstrate the potential usefulness of gene clusters or subsets of gene clusters in classification using metagenomic data.

### Conclusions

In summary, our results indicate that different data representations should be investigated when using machine learning based on microbiome data to predict disease conditions. We showed that comparable prediction can be obtained using taxonomical composition and gene clusters. Selecting subsets of genes based on public databases or manually curated properties associated with a scientific hypothesis can provide improved accuracy and interpretability for the prediction of some pathologies. When using representation based on granular features such as gene clusters, algorithms producing sparse models should be considered, as they provide biomarkers that can potentially be ported to independent validation cohorts or even be tested *in vitro*.

## MATERIALS AND METHODS

### Production of the different data types

The taxonomic and functional profiles produced, respectively, with MetaPhlAn3 and HUMAnN3 (13) were downloaded via the R package curatedMetagenomicData (version 1.20.0) (58). The IBD and T2D datasets were downloaded from NCBI Sequence Read Archive under accession numbers ERA000116 and PRJNA422434, respectively. The OB, CRC, and LC datasets were downloaded from the European Nucleotide Archive (ENA) under the accession numbers PRJEB4336, PRJEB6070, and PRJEB6337 , respectively. The gene clusters matrices were produced following three steps: the assembly of raw reads into contigs using MEGAHIT (version 1.2.9) (59), the prediction of protein-coding genes and their subsequent translation into amino acids using Prodigal with the default parameters (version 2.6.3) (60), and the clustering of the resulting amino acids sequences at a 70% identity threshold using CD-HIT (version 4.7) (61) with parameters -c 0.70 -aS 0.90 -d 0 -M 0 -T 0 -g 1 -G 0. To measure the effect of clustering parameters on prediction performances, we evaluated identity thresholds *c* and coverage thresholds *aS* in the sets {0.70, 0.85, 0.95} and {0.70, 0.90}, respectively. Values 0.70 and 0.90 for *c* and *aS* had overall results slightly higher than the other combinations (Supplementary Figure S46) and were thus selected for the subsequent steps. The fusion data type was produced by the concatenation of three tables: the taxonomic and functional profiles and the gene clusters.

To produce the filtered tables of gene clusters, we first clustered together both the gene-coding sequences from the databases and the gene-coding sequences of the samples. The sequences used for this analysis were from the CAZy (30), COG (62), MERGEM (31), BRENDA (33), and MIBiG (32) databases. Next, we kept every gene cluster that contained at least one sequence from the databases and one sequence from the samples. To select the gene clusters containing genes potentially implicated in the production or the degradation of neurotransmitters and endocannabinoids analogs, we constructed a local database of Hidden Markov Model (HMM) profiles of protein domains (from Pfam) found in enzymes included in the pathways of synthesis and degradation of neurotransmitters, endocannabinoids, and endocannabinoids analogs (Supplementary Table S2). We then searched for these HMM profiles in the sequences of each gene cluster’s representative sequence with the command hmmsearch (-E 0.001) from HMMER (63) and kept every gene cluster whose representative sequence matched at least one of the HMM profiles. For all data types, features present only in one sample were discarded because they were deemed uninformative.

### Machine learning protocol

The machine learning protocol was developed in Python (version 3.6.3) with scikit-learn (version 0.22.1). SCM (28) (https://github.com/aldro61/pyscm) and rSCM (29) (https://github.com/thibgo/randomscm) algorithms were downloaded via their Github repository.

The classification performances were assessed by a 10-fold cross-validation, repeated on 10 independent runs, yielding 100 different data splits. For each split, the training set (90% of examples) and testing set (10% of examples) were separated before feature selection. Therefore, the test set was not seen by the algorithm before the final evaluation of the models built on the training set. The first step of the protocol was the application of a simple RF—scikit-learn’s RandomForestClassifier with 100 decision trees (*n_estimators*) and all other hyperparameters set at default—on all remaining features after the preprocessing steps. The features were sorted according to the “*features_importances_”* method, which is a score derived from the Gini impurity, where the higher the score is, the more important is the feature. The features having an importance greater than the mean of importances were selected and used for the training of the final model. For every algorithm, an array of hyperparameters was tested, and the combination yielding the best score was kept. To take into account the imbalanced classes of every classification task, we evaluated model performances with the balanced accuracy score. Models were compared using a generalized linear model (GLM) for each dataset with the NLME R package (version 3.1–148). Balanced accuracies have been normalized using ranked values fitted into LME (~ data representation * algorithm), and significance has been tested by analysis of variance (ANOVA) with random effects nested within sample splits. Specific comparisons were evaluated using the Contrast R package (version 0.22). *P*-values corrected for multiple tests using a false discovery rate <0.05 were considered significant.

### Algorithms and hyperparameters

SVMs are algorithms that aim at finding the hyperplane that maximizes the margin between the samples from each class (64). When the classes are not linearly separable, kernel functions can be used to map data to a higher dimensional space to find the hyperplane. For hyperparameters tuning, the regularization parameter C was chosen in the set {10^−4^, 10^−3^, 10^−2^, 10^−1^, 0.25, 0.5, 0.8, 0.9, 1, 10, 100, 500, 1,000} for both the SVM with linear and rbf kernels. The same values were used for the C parameter in L1- and L2-regularized LR. For the DT, the maximum depth of the tree was in {1, 3, 5, 10, 25}. The minimum number of samples required to split a node was in {2, 5, 10}, and the minimum number of samples required to be at a leaf node was in {1, 2, 4}. For RF, the number of trees was fixed at 500. The minimum number of samples required to split a node, the maximum depth of the trees, and the minimum number of samples required to be at a leaf node were in {2, 5, 10}, {1, 3, 5, 10, 25}, and {1, 2, 4}, respectively. The SCM is a rule-based algorithm that learns conjunctions (logical-AND) and disjunctions (logical-OR) which are logical combinations of rules (28, 56). For SCM, the trade-off parameter for the utility function was in {0.5, 1, 2}, the maximum number of rules was in {1, 2, 3, 4, 5}, and the type of model varied between conjunction and disjunction. The rSCM is an ensemble algorithm that uses SCM with bootstrap aggregating. For rSCM, the number of base estimators was in {30, 100}, the proportions of samples and features used to train each estimator were both in {0.6, 0.85}, and the trade-off parameter p for the utility function was in {0.1, 0.316, 0.45, 0.562, 0.65, 0.85, 1.0, 2.5, 4.39, 5.623, 7.623, 10.0}.

## Data Availability

The bioinformatics and machine learning protocols used in this work can be accessed via our Github repository: https://github.com/fredericraymond/MLCOG.

## SUPPLEMENTAL MATERIAL

Supplemental Figures msystems.00531-23-s0001.pdf Figures S1 to S46.

Supplemental Tables msystems.00531-23-s0002.xlsx Tables S1 and S2.

ASM does not own the copyrights to Supplemental Material that may be linked to, or accessed through, an article. The authors have granted ASM a non-exclusive, world-wide license to publish the Supplemental Material files. Please contact the corresponding author directly for reuse.
