# Supplementary material for: Gene-based microbiome representation enhances host phenotype classification
Source: mSystems. 2023 Jul 5;8(4):e00531-23. doi: 10.1128/msystems.00531-23 (PMC10469787; doi:10.1128/msystems.00531-23)

Supplementary Figure S1 − Performance of OB classification quantified with balanced accuracy for all algorithms

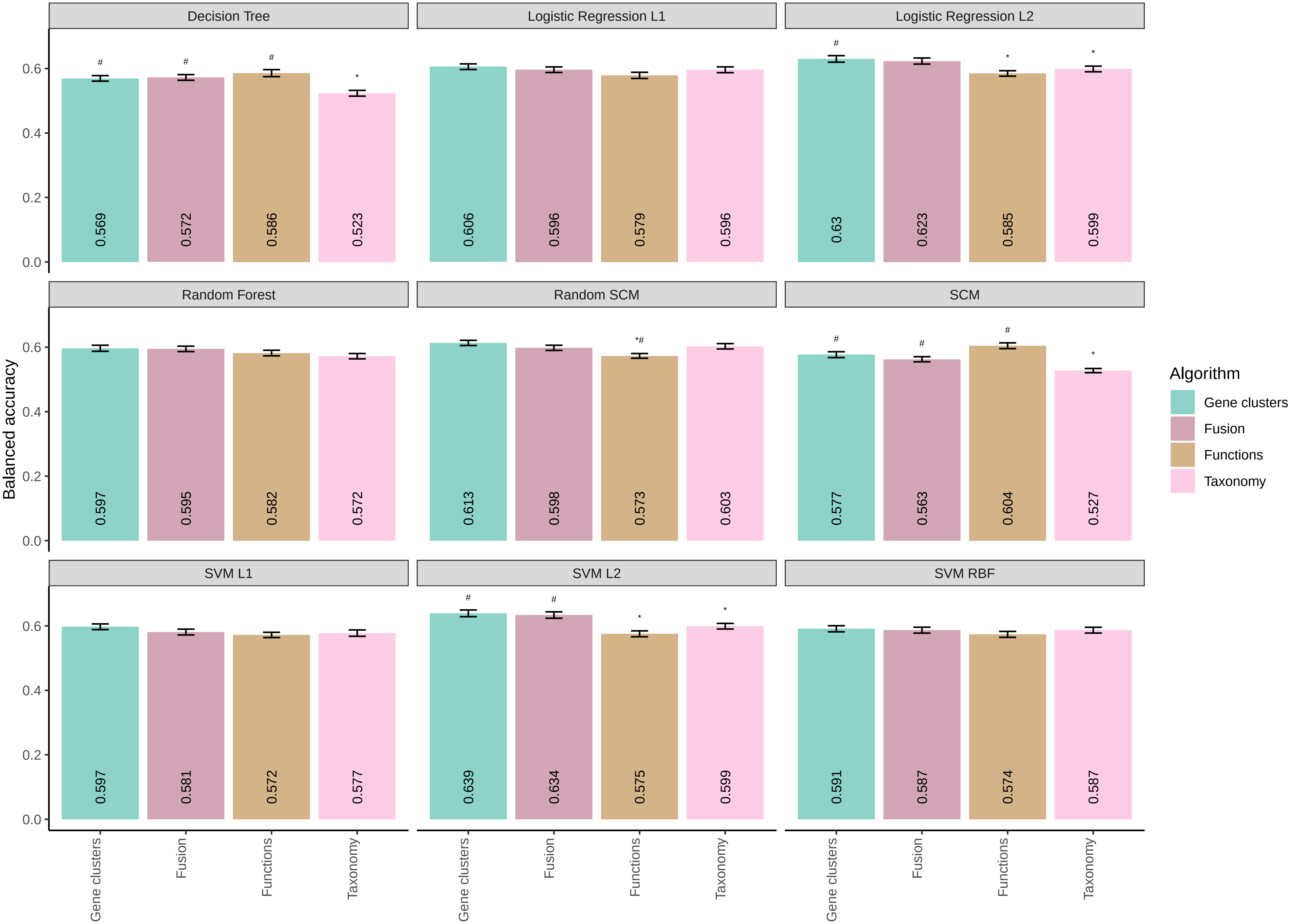

Supplementary Figure S2 − Performance of T2D classification quantified with balanced accuracy for all algorithms

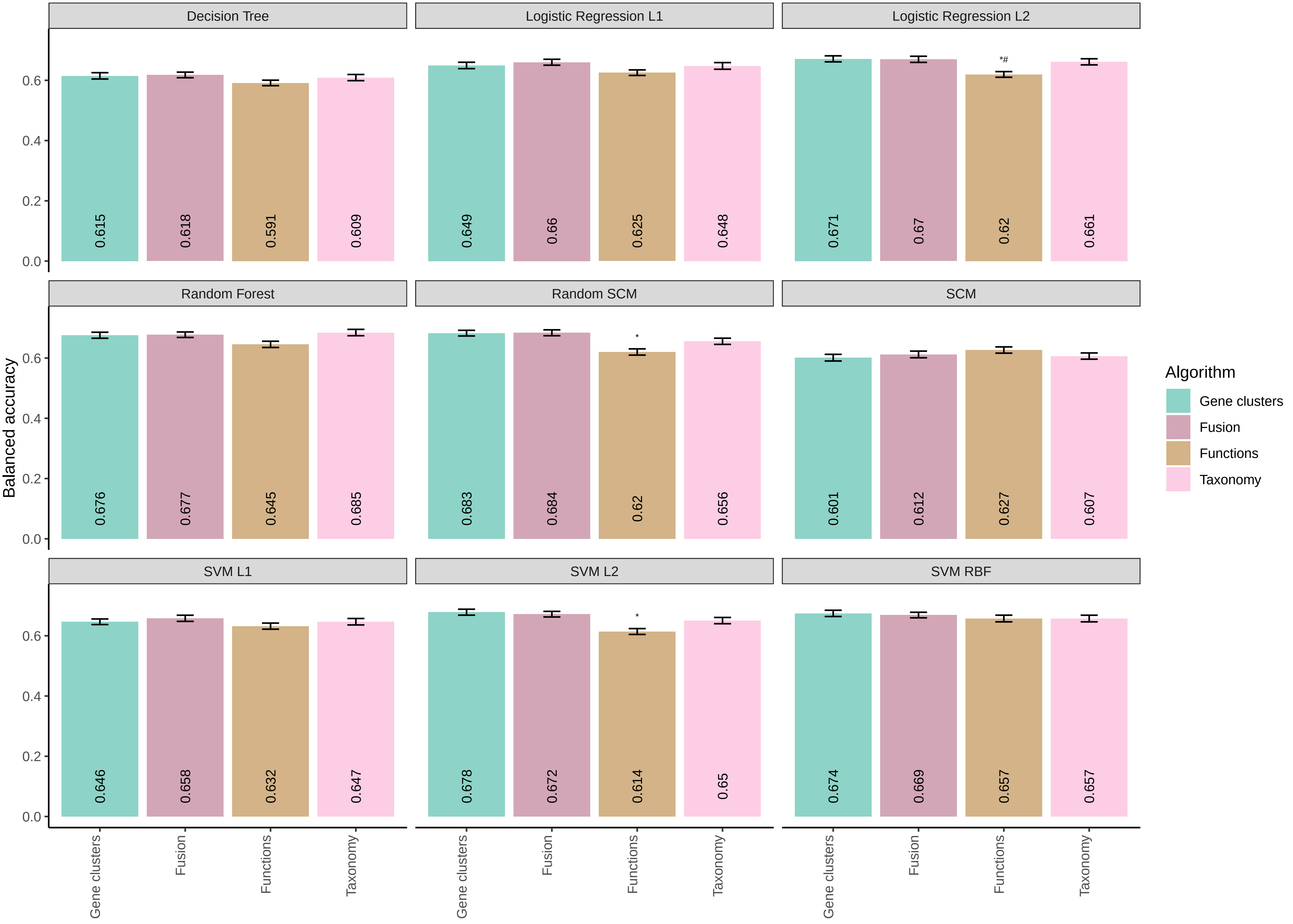

Supplementary Figure S3 − Performance of IBD classification quantified with balanced accuracy for all algorithms

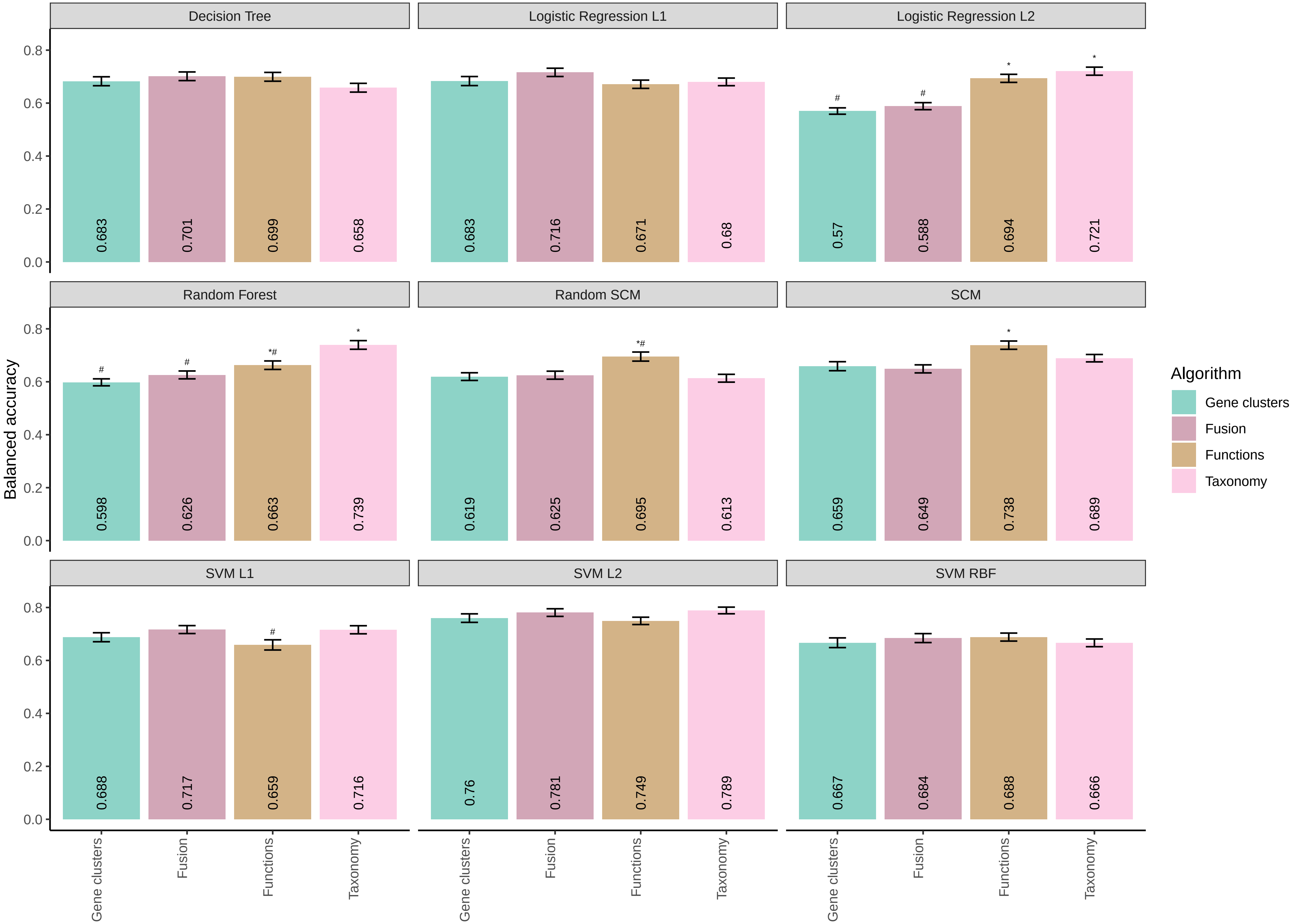

Supplementary Figure S4 − Performance of LC classification quantified with balanced accuracy for all algorithms

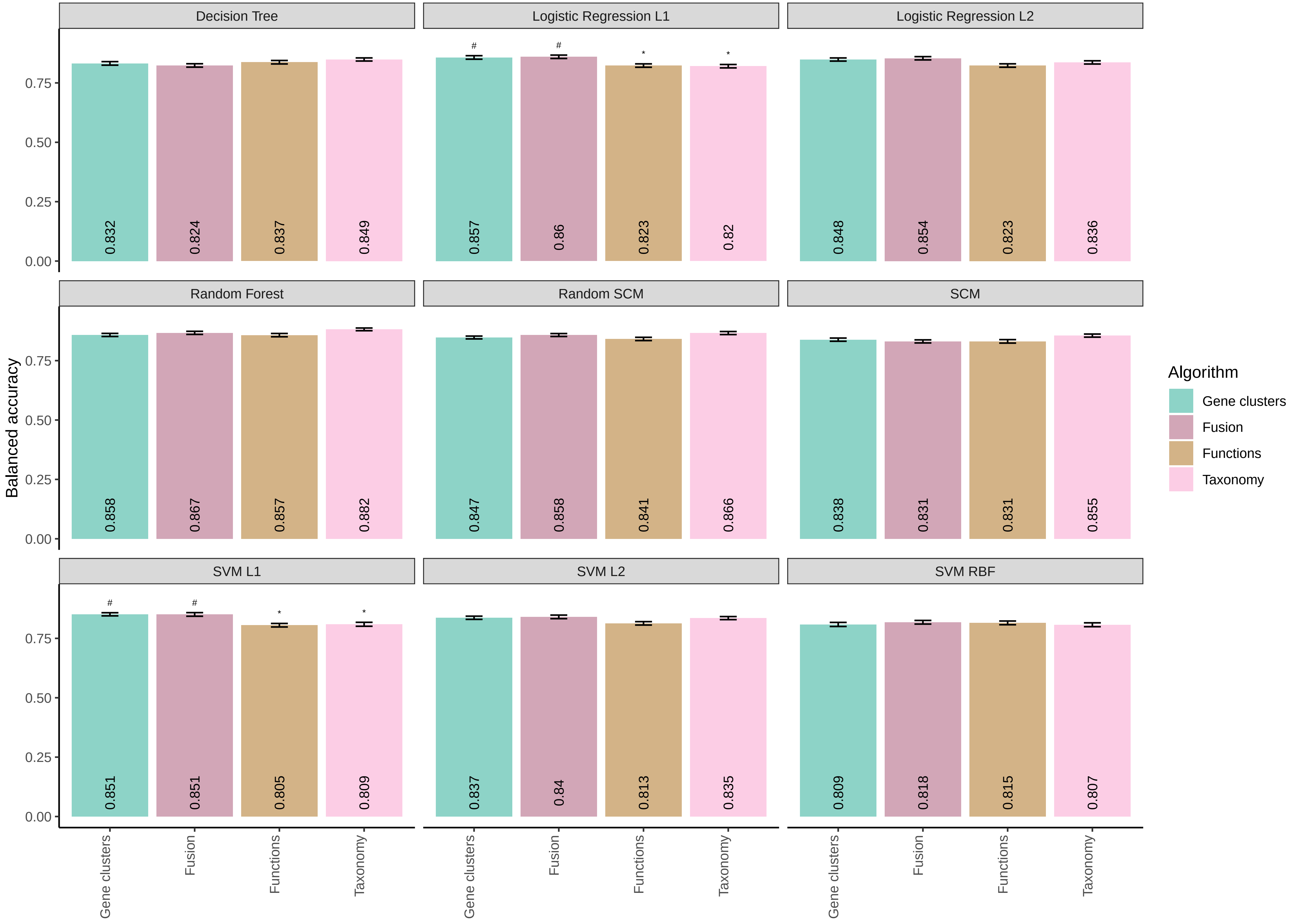

Supplementary Figure S5 − Performance of CRC classification quantified with balanced accuracy for all algorithms

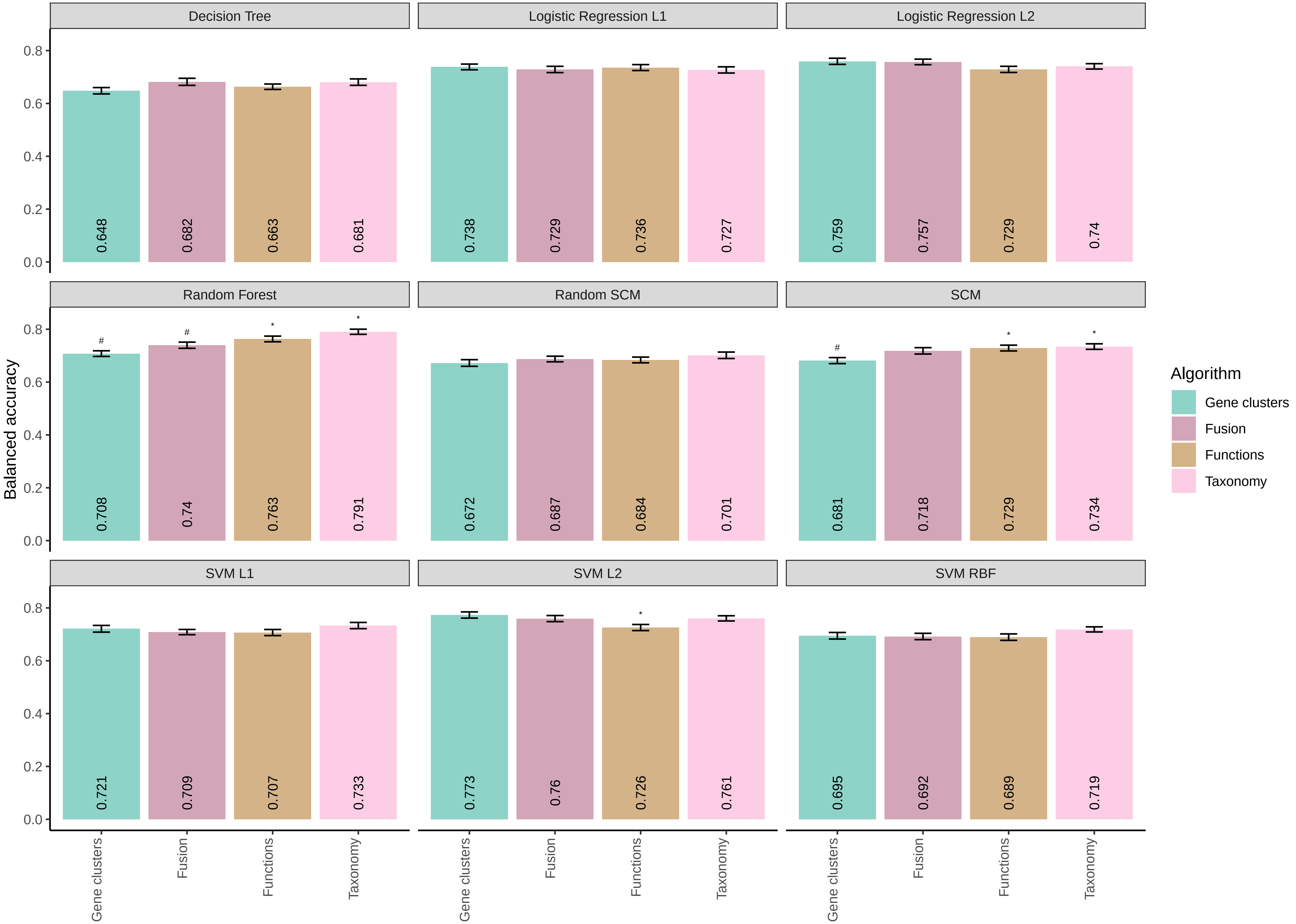

Supplementary Figure S6 − Performance of OB classification quantified with F1 score for all algorithms

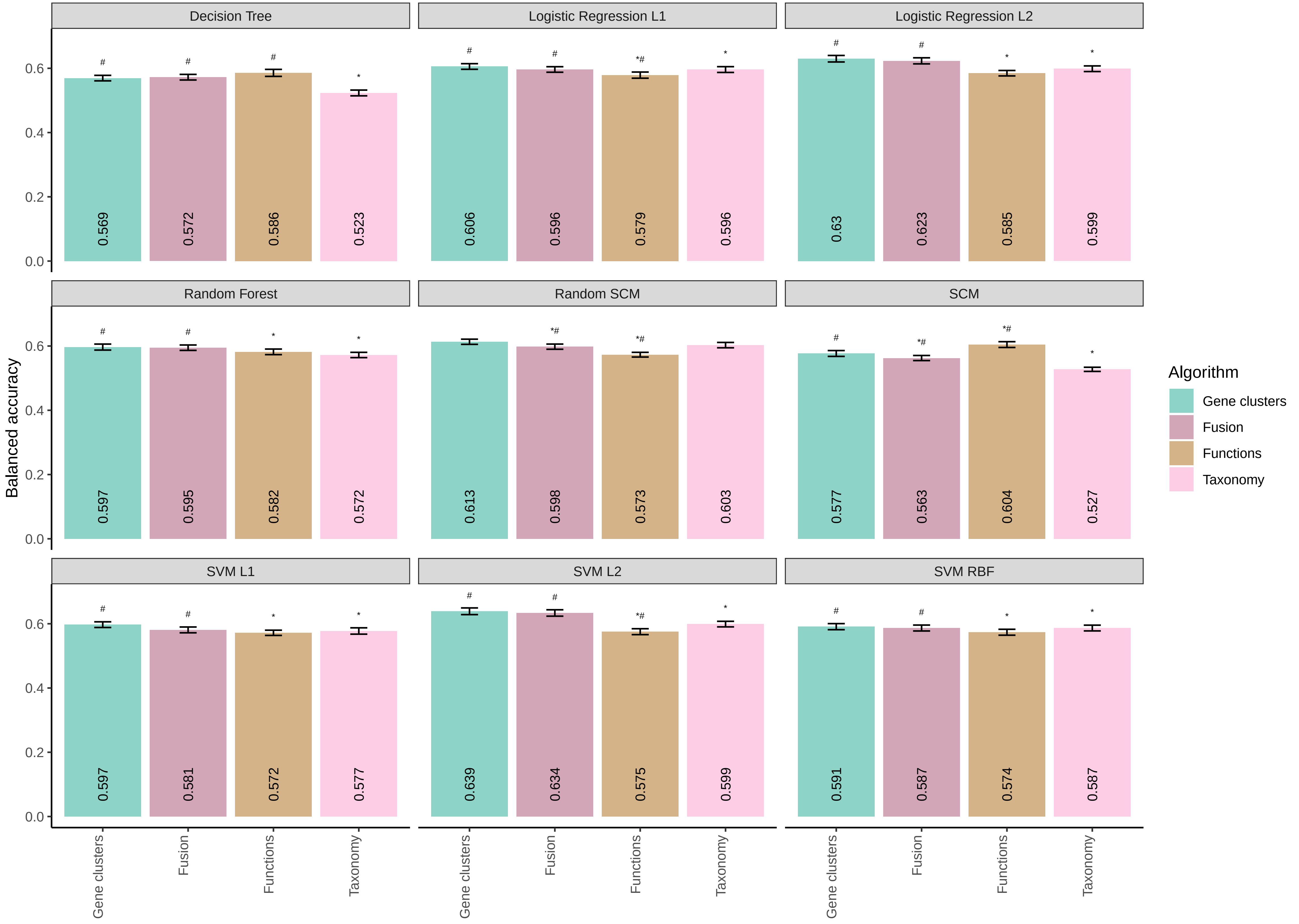

Supplementary Figure S7 − Performance of T2D classification quantified with F1 score for all algorithms

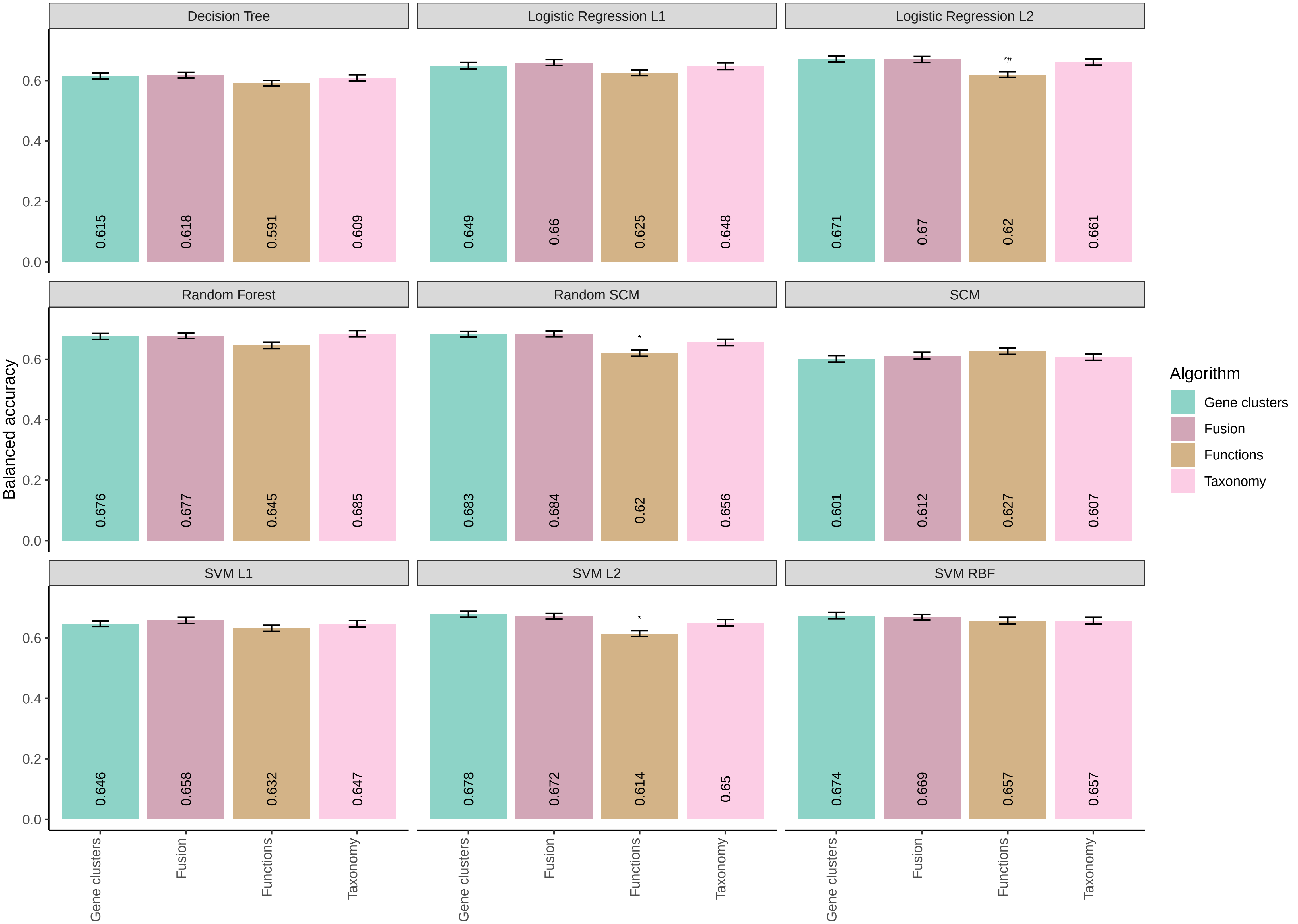

Supplementary Figure S8 − Performance of IBD classification quantified with F1 score for all algorithms

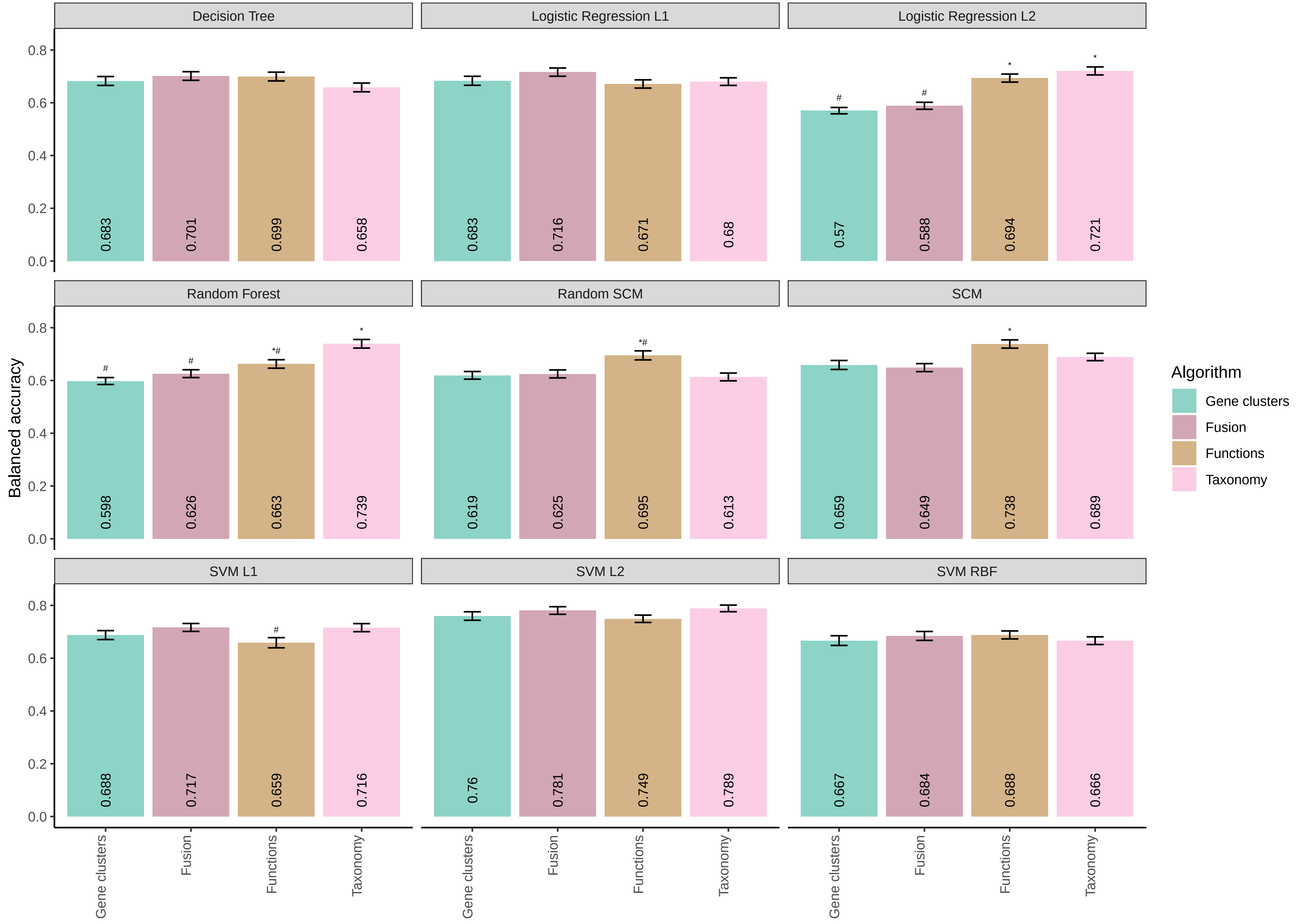

Supplementary Figure S9 − Performance of LC classification quantified with F1 score for all algorithms

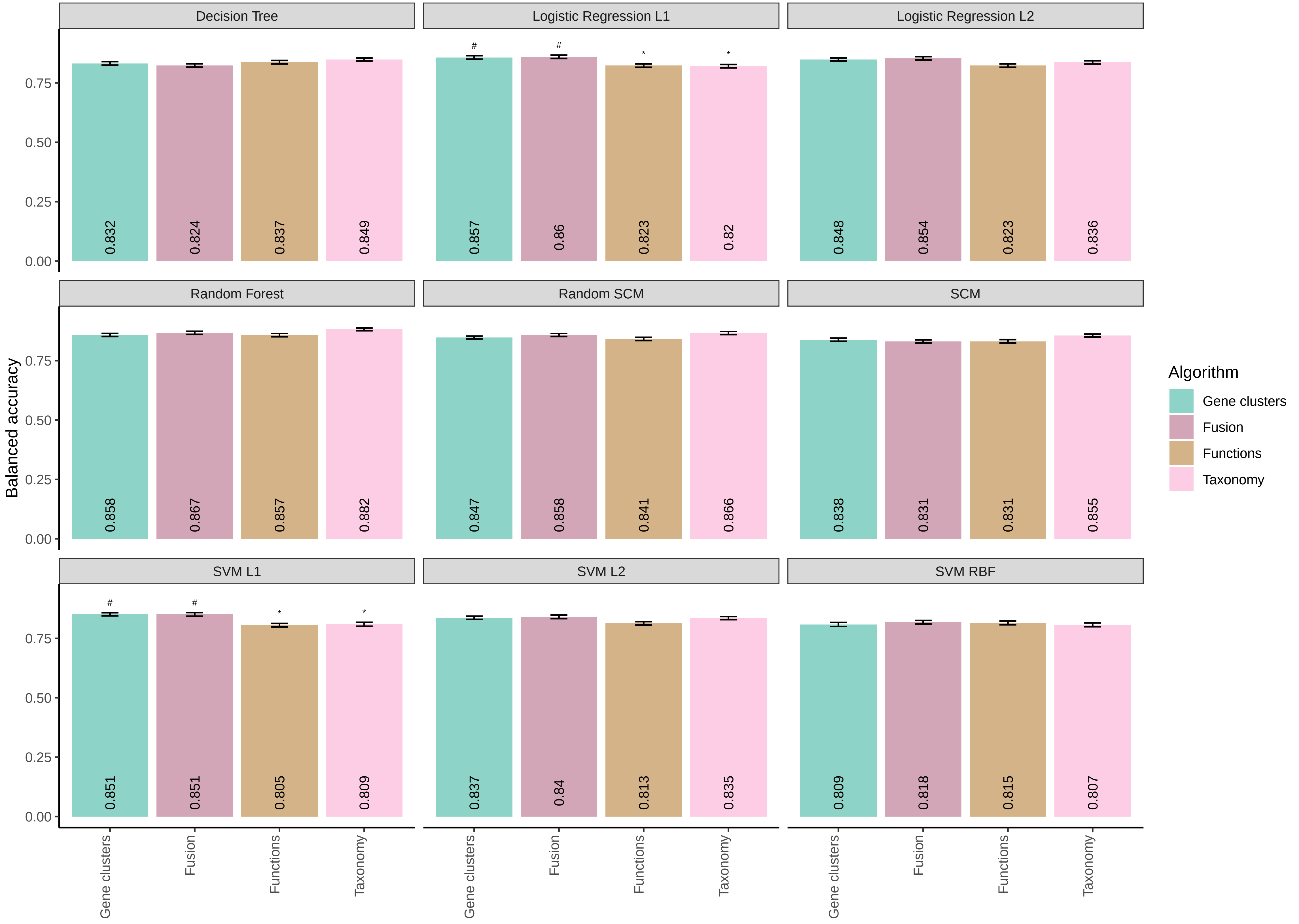

Supplementary Figure S10 − Performance of CRC classification quantified with F1 score for all algorithms

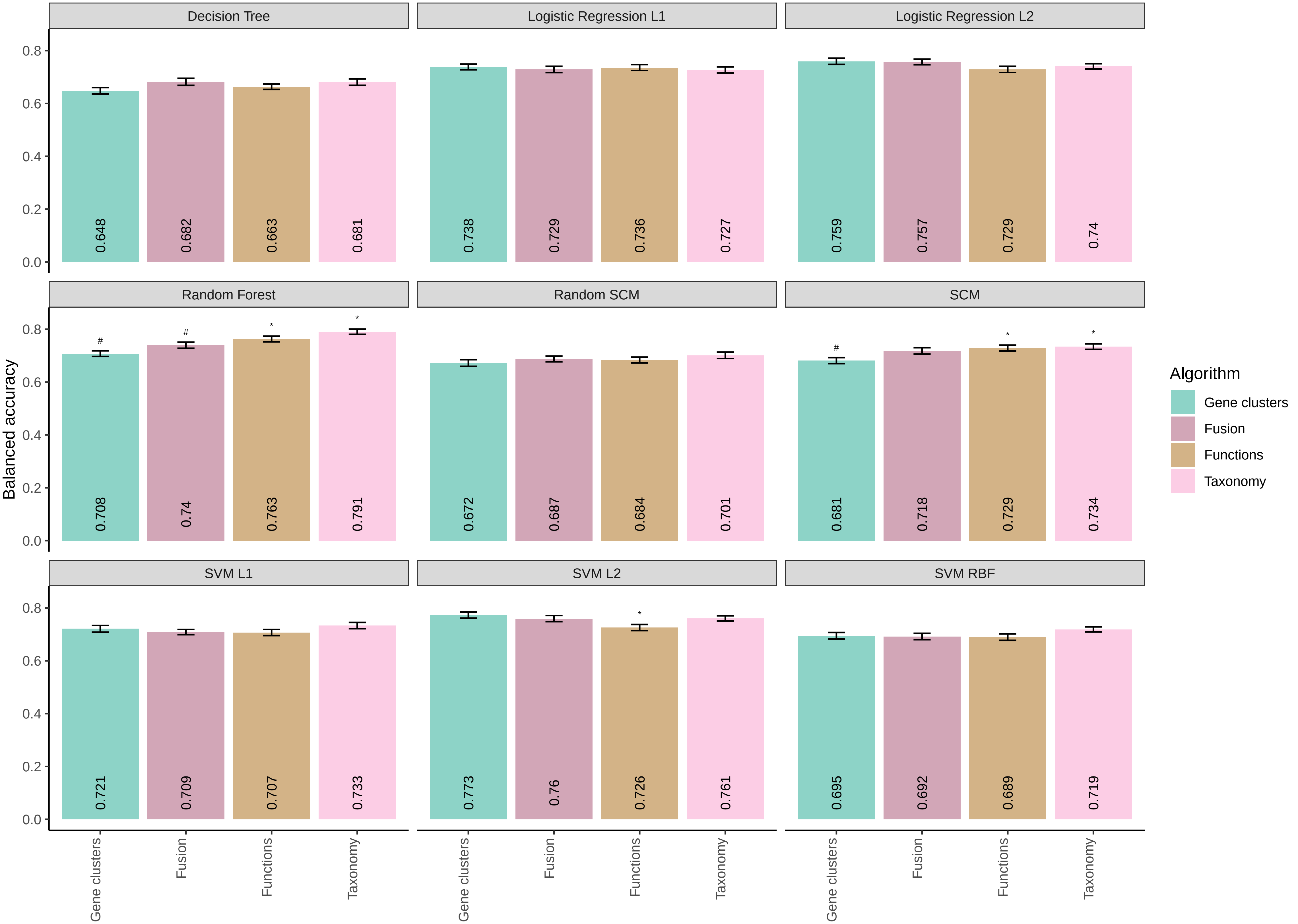

Supplementary Figure S11 − Performance of OB classification quantified with rocAUC for all algorithms

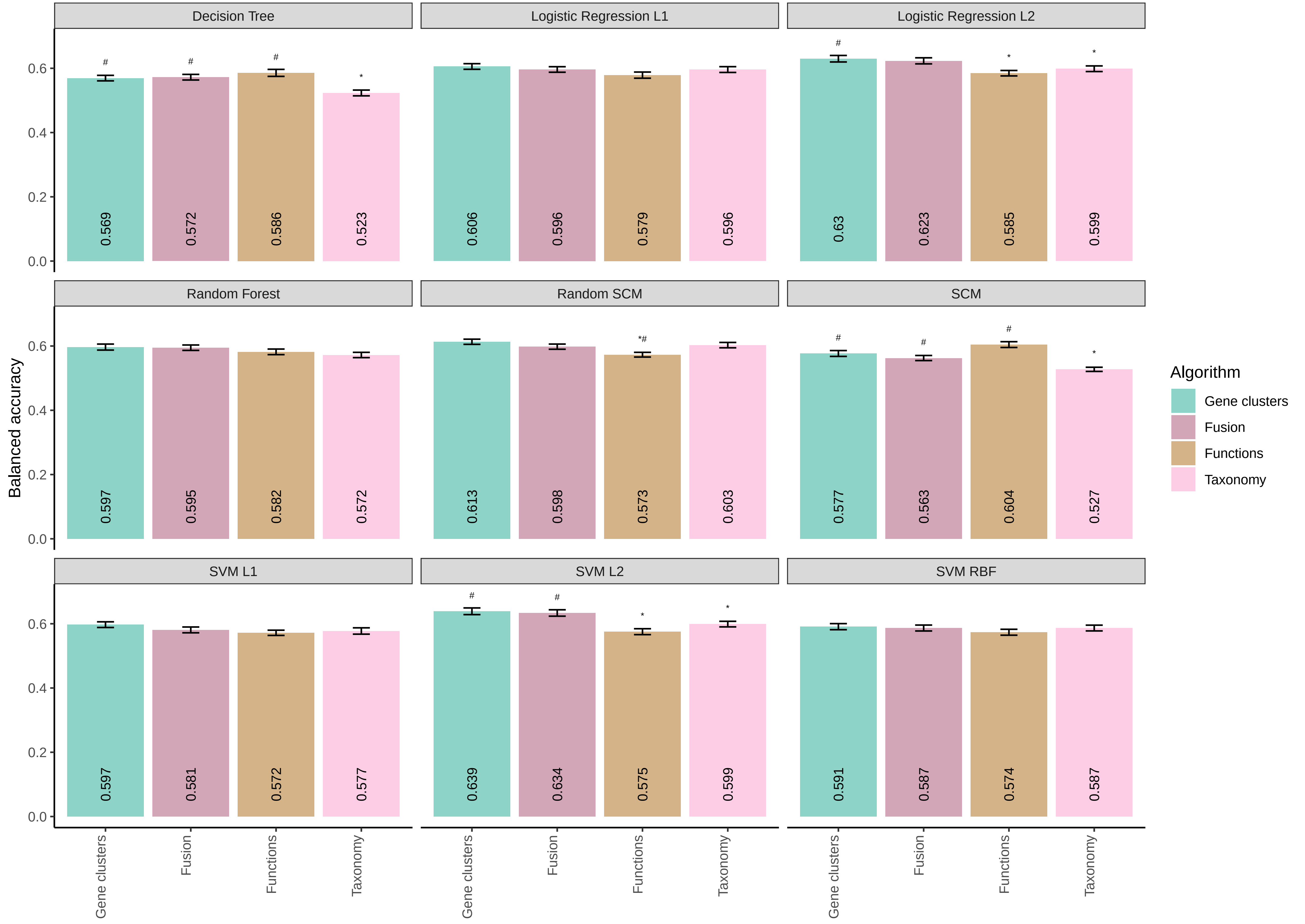

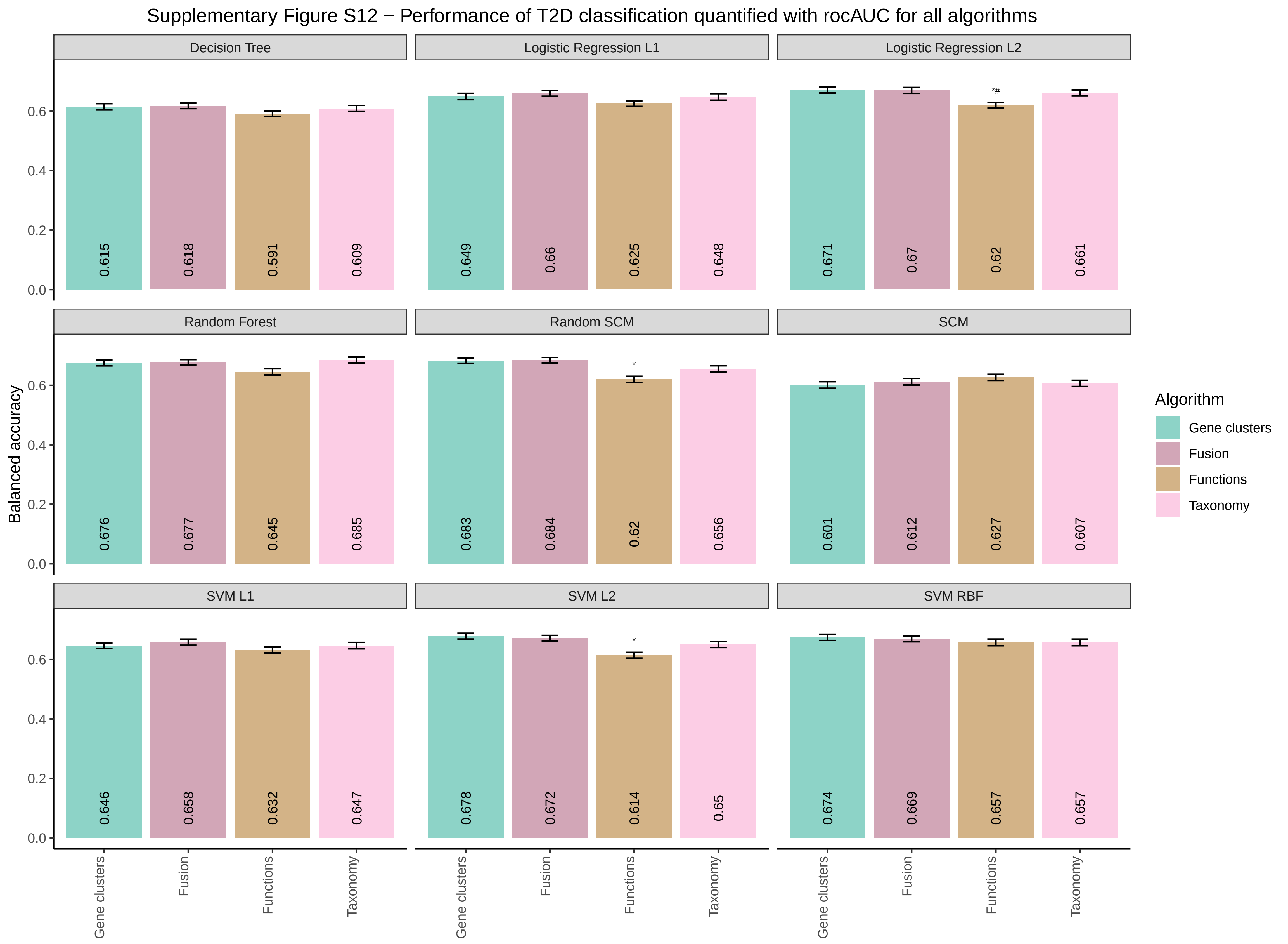
Supplementary Figure S12 − Performance of T2D classification quantified with rocAUC for all algorithms
Decision Tree
Logistic Regression L1
Logistic Regression L2
Random Forest
Random SCM
SCM
SVM L1
SVM L2
SVM RBF
Balanced accuracy
0.0
0.2
0.4
0.6
0.615
0.618
0.591
0.609
0.649
0.66
0.625
0.648
0.671
0.67
0.62
0.661
*#
0.676
0.677
0.645
0.685
0.683
0.684
0.62
0.656
*
0.601
0.612
0.627
0.607
0.646
0.658
0.632
0.647
0.678
0.672
0.614
0.65
*
0.674
0.669
0.657
0.657
Gene clusters
Fusion
Functions
Taxonomy
Algorithm
Gene clusters
Fusion
Functions
Taxonomy

Supplementary Figure S13 − Performance of IBD classification quantified with rocAUC for all algorithms

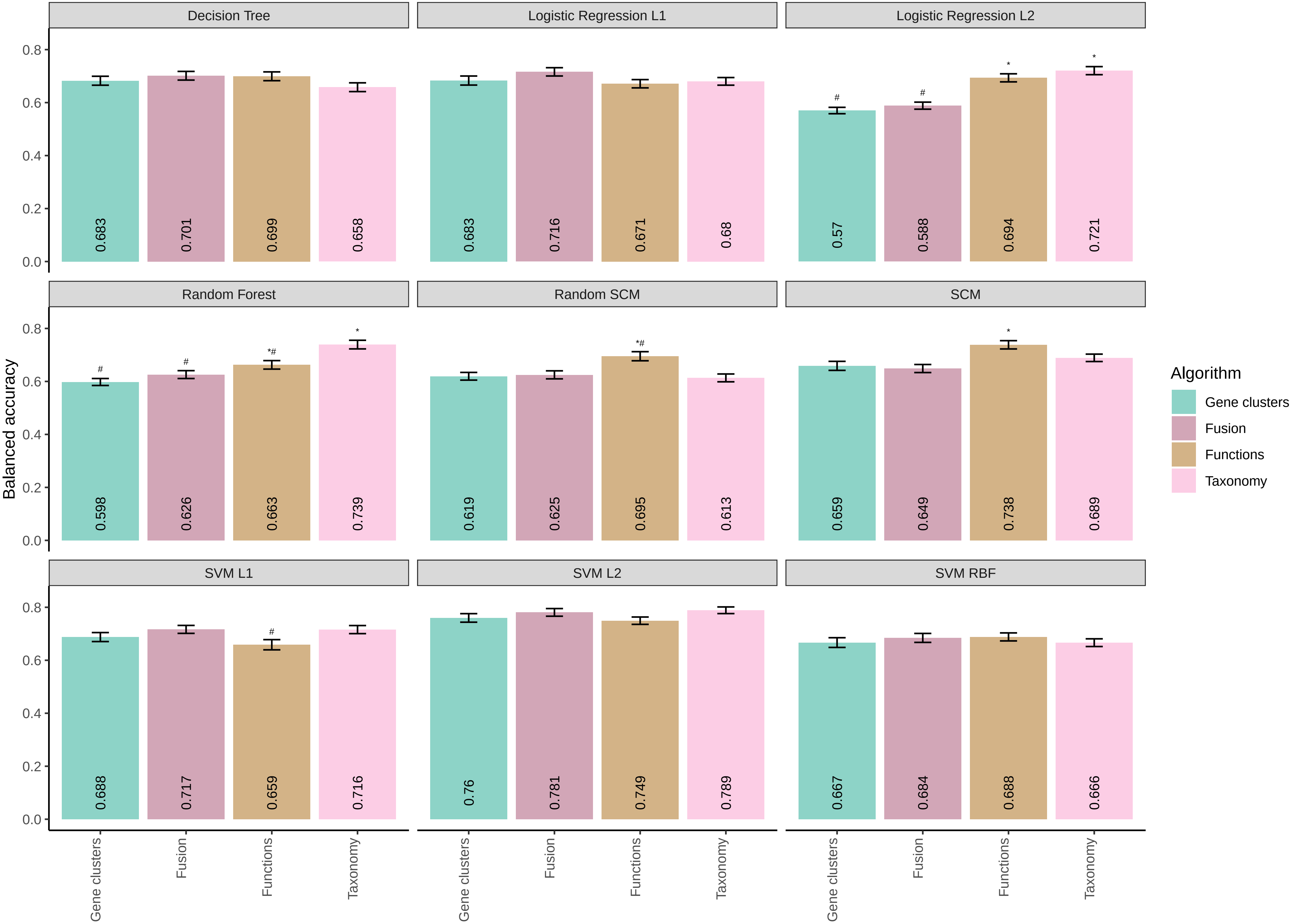

Supplementary Figure S14 − Performance of LC classification quantified with rocAUC for all algorithms

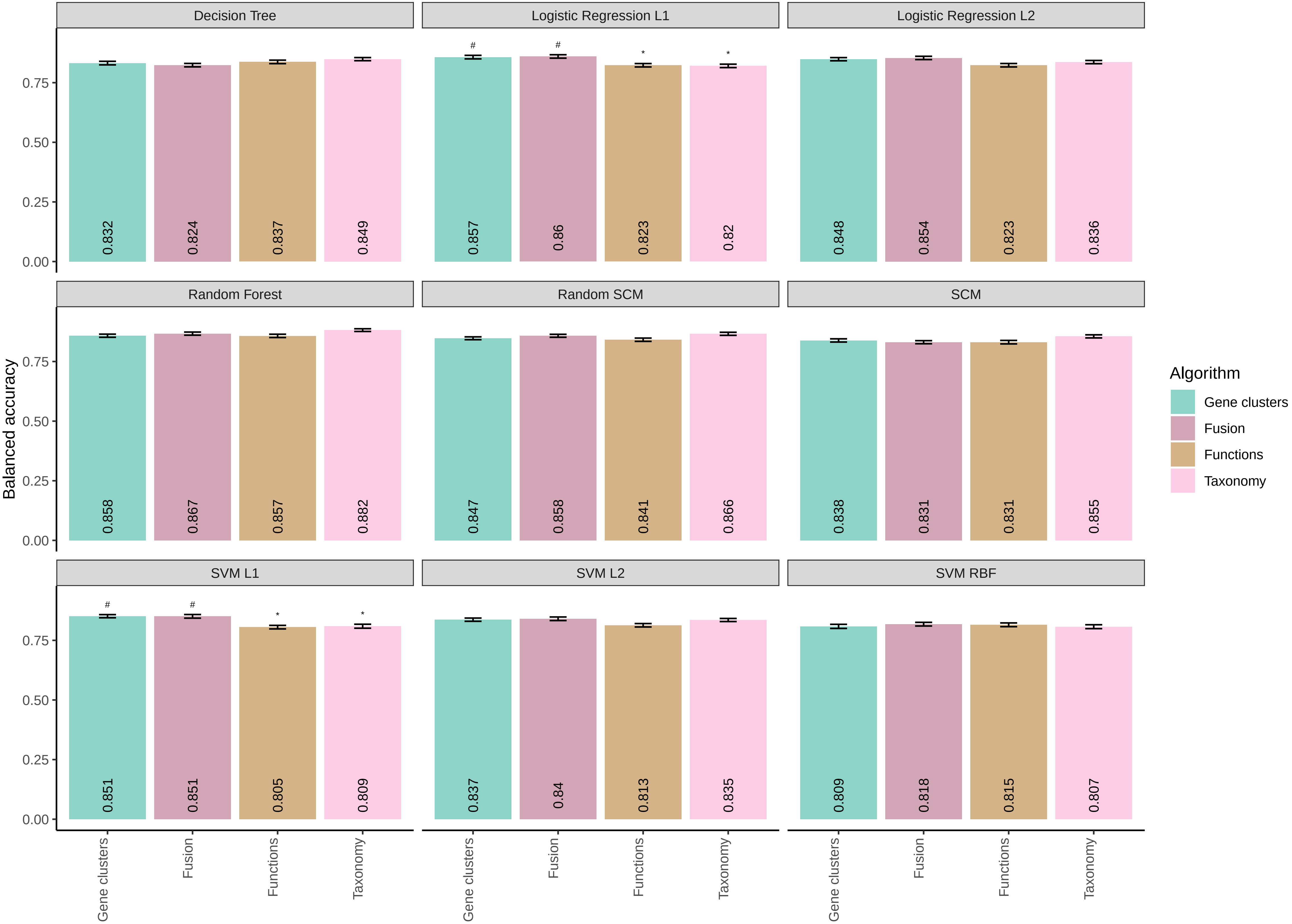

Supplementary Figure S15 − Performance of CRC classification quantified with rocAUC for all algorithms

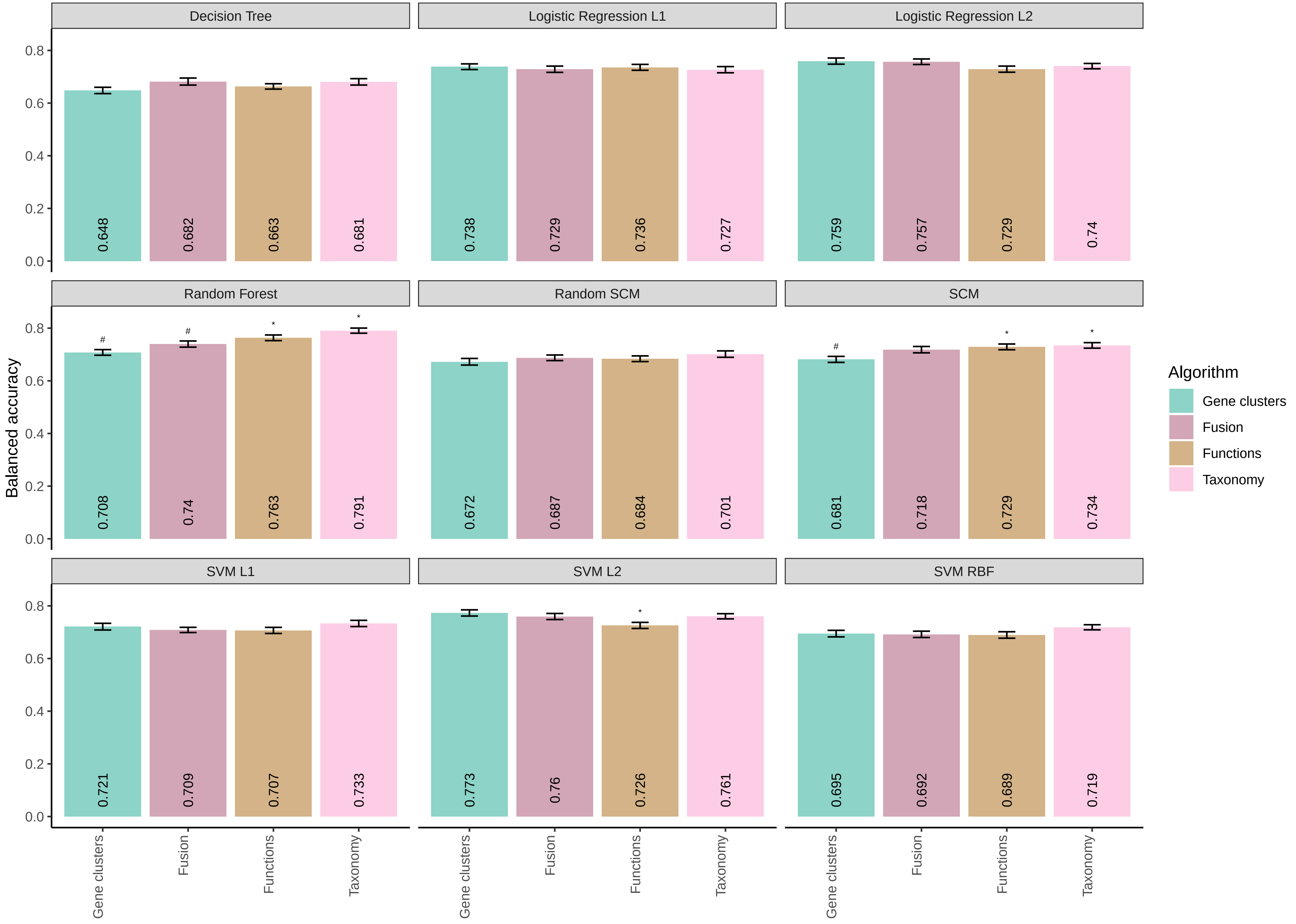

Supplementary Figure S16 − Performance of OB classification quantified with balanced accuracy for all algorithms

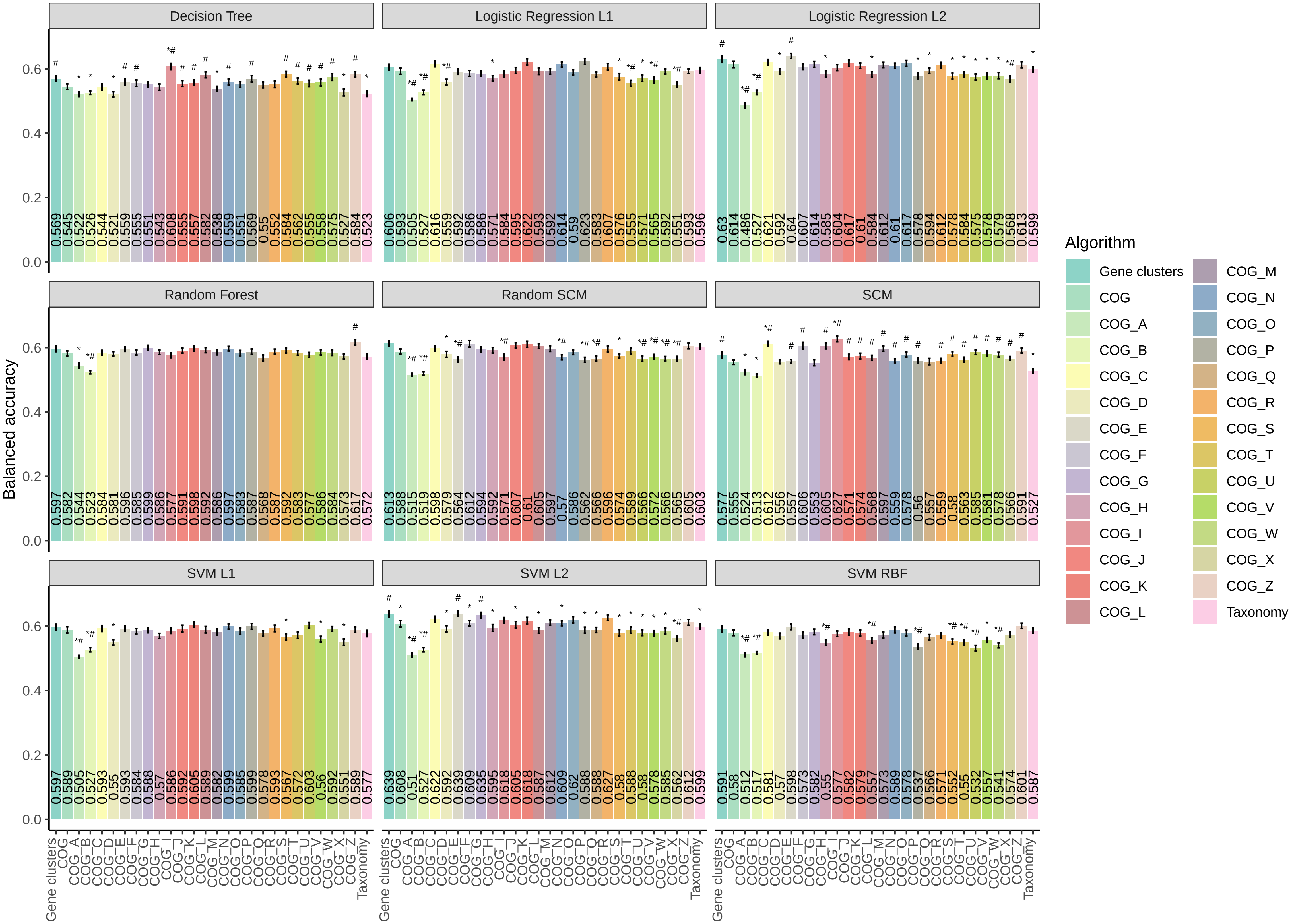

Supplementary Figure S17 − Performance of T2D classification quantified with balanced accuracy for all algorithms

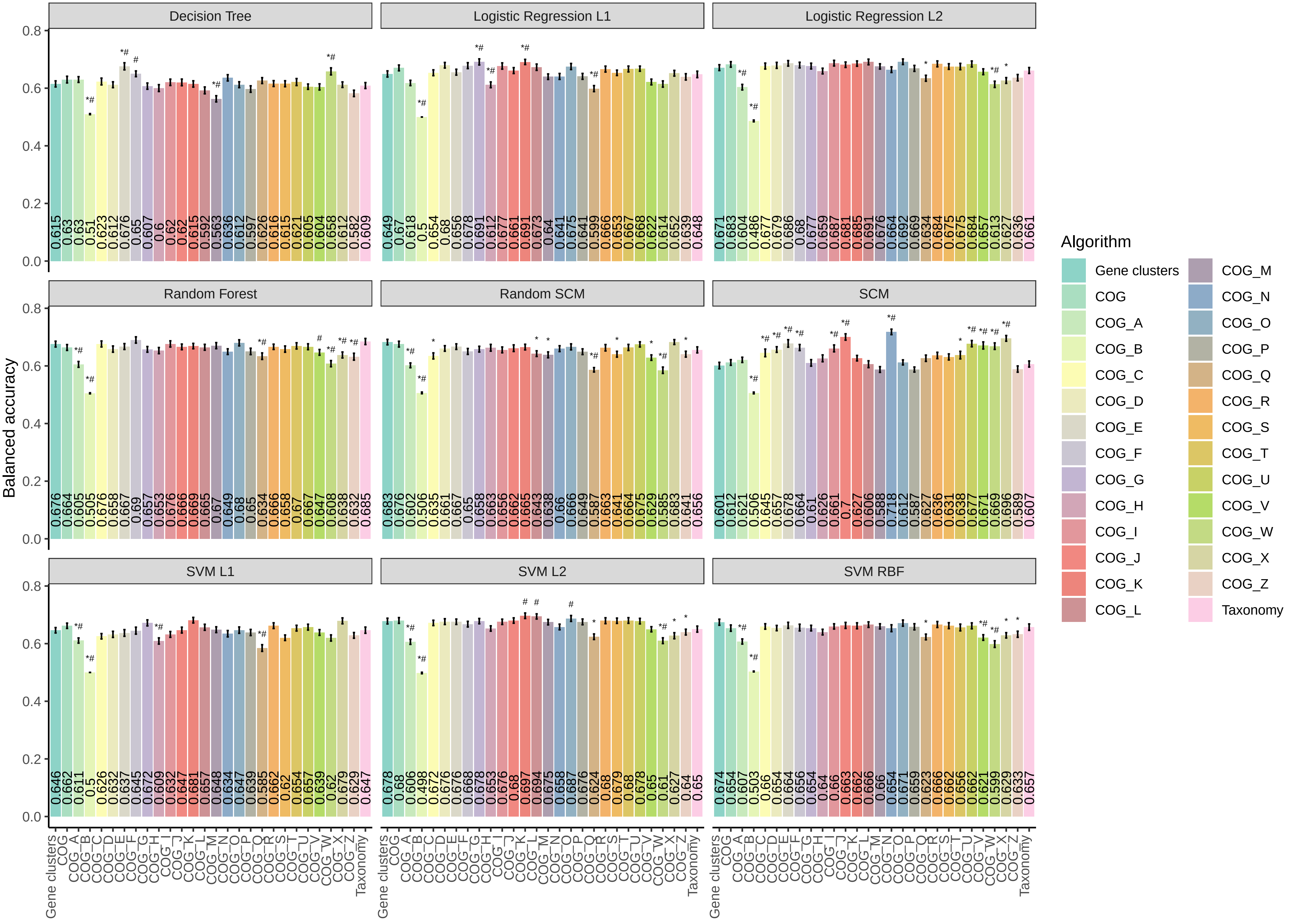

Supplementary Figure S18 − Performance of IBD classification quantified with balanced accuracy for all algorithms

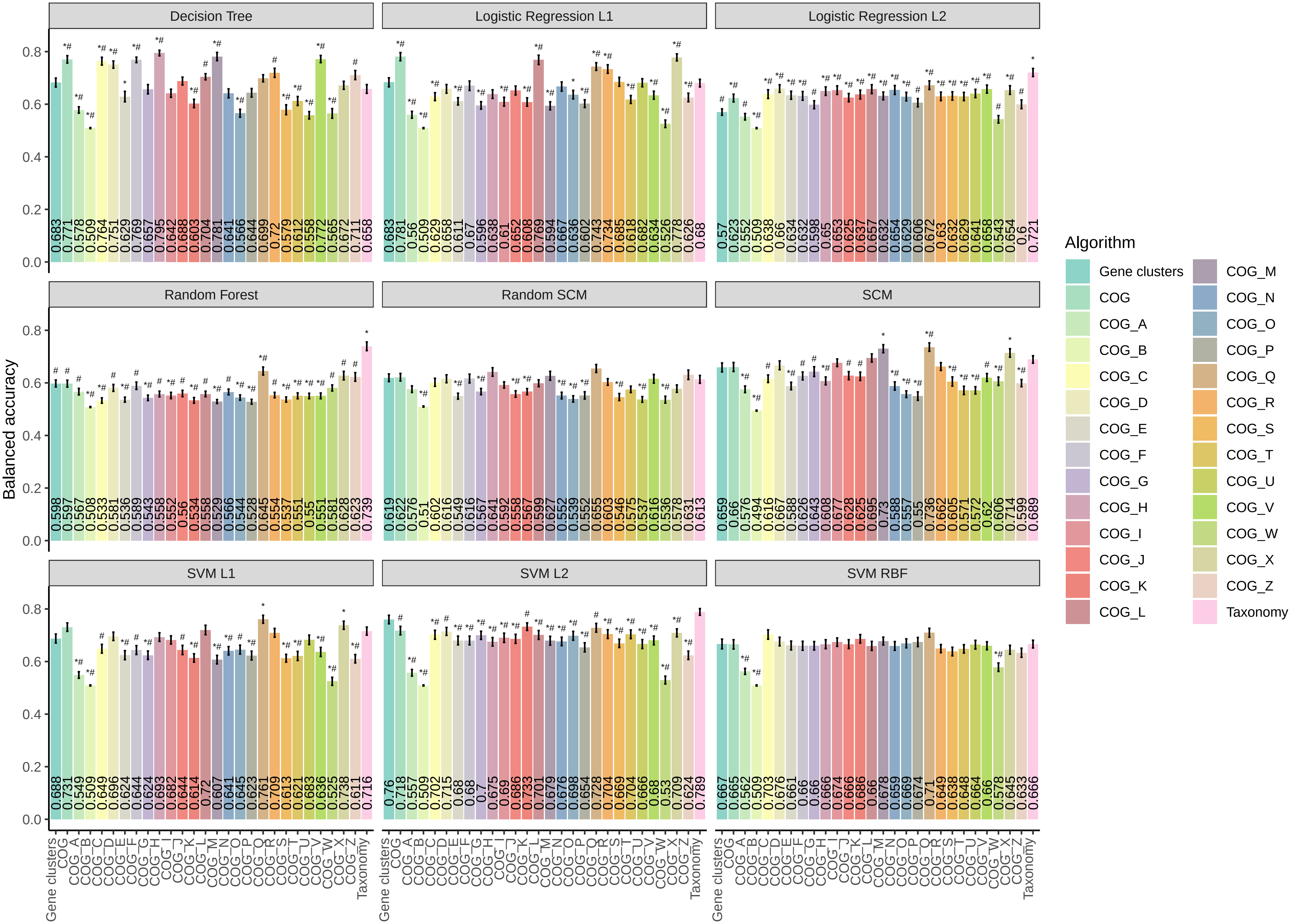

Supplementary Figure S19 − Performance of LC classification quantified with balanced accuracy for all algorithms

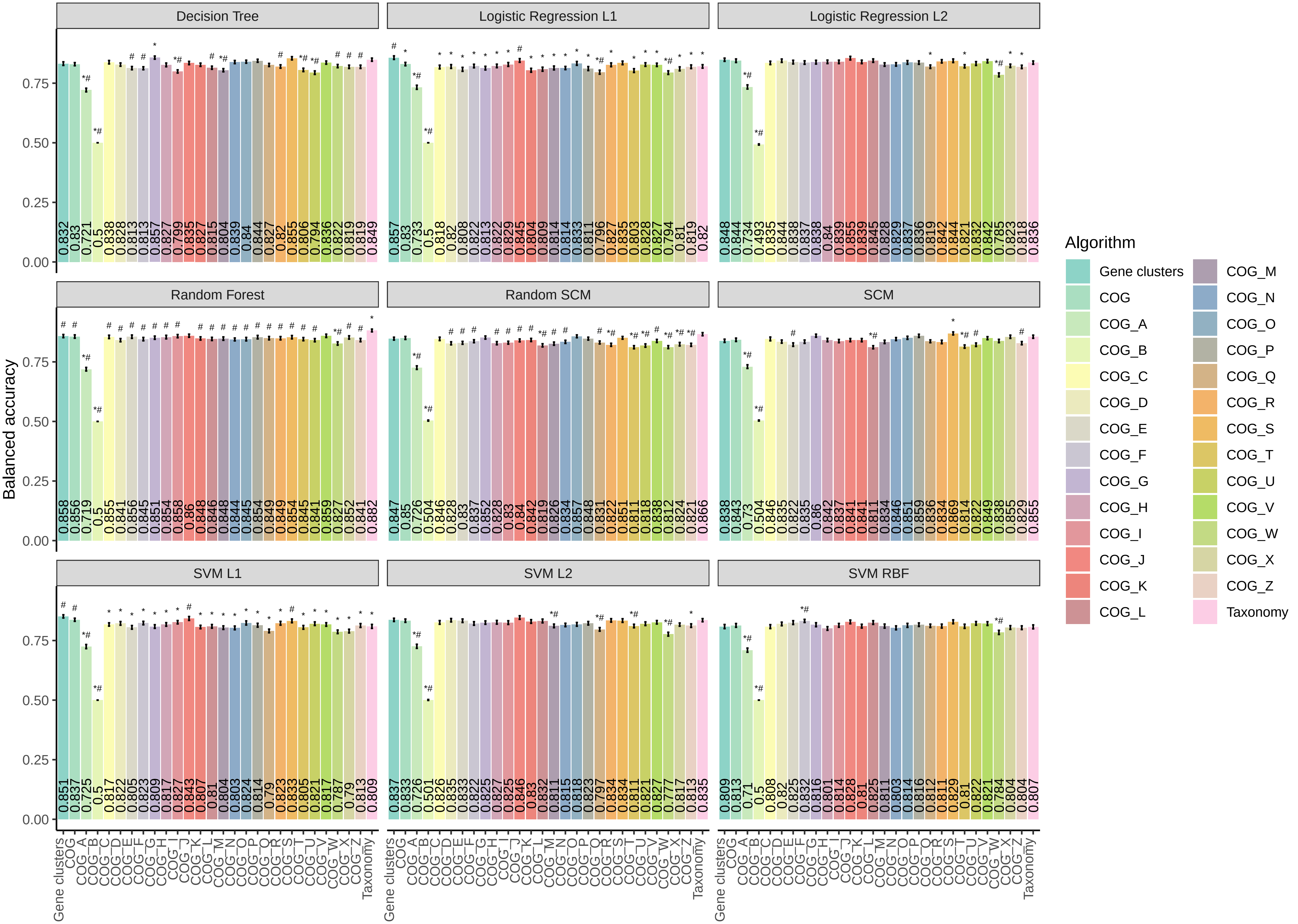

Supplementary Figure S20 − Performance of CRC classification quantified with balanced accuracy for all algorithms

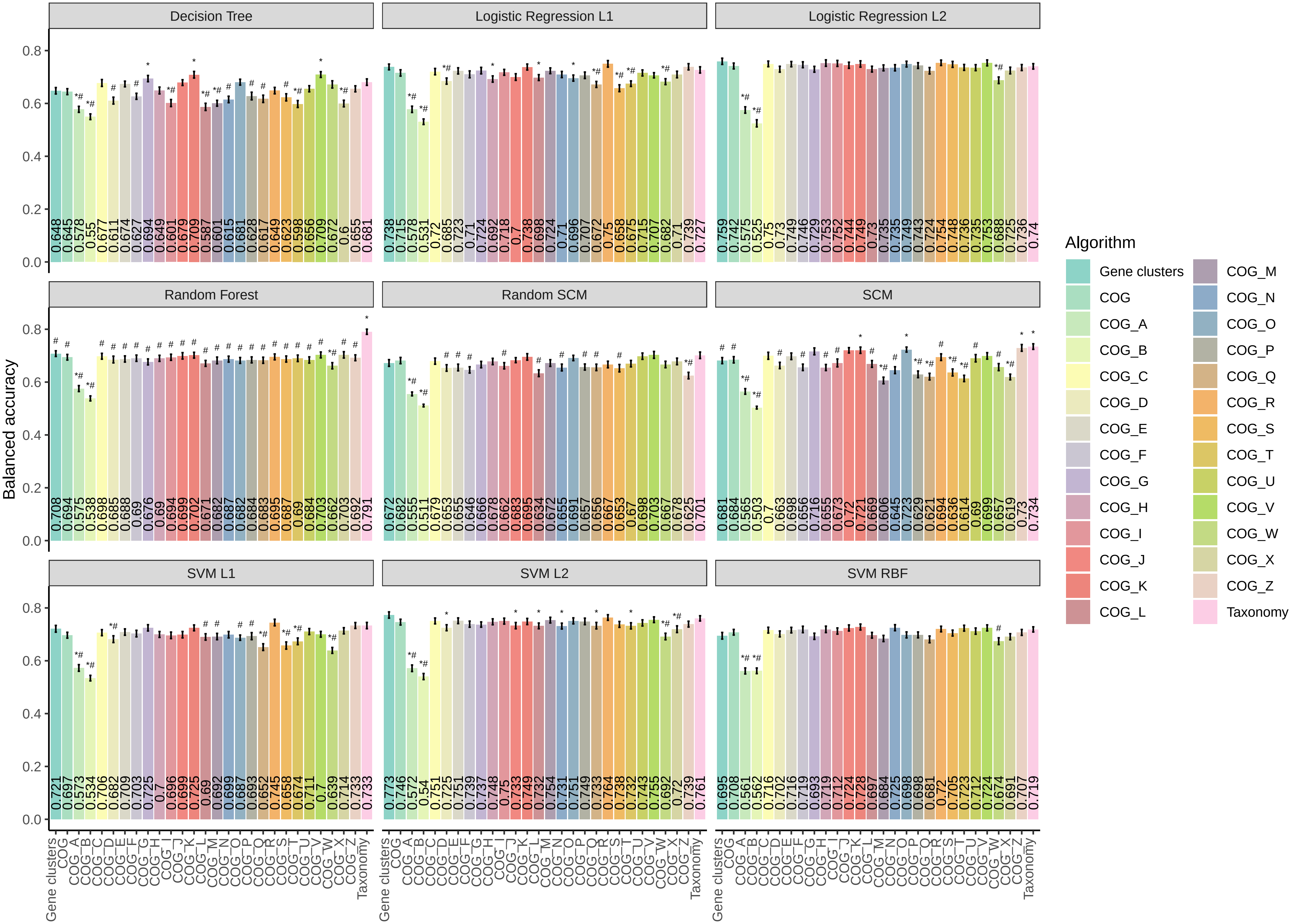

Supplementary Figure S21 − Performance of OB classification quantified with F1 score for all algorithms

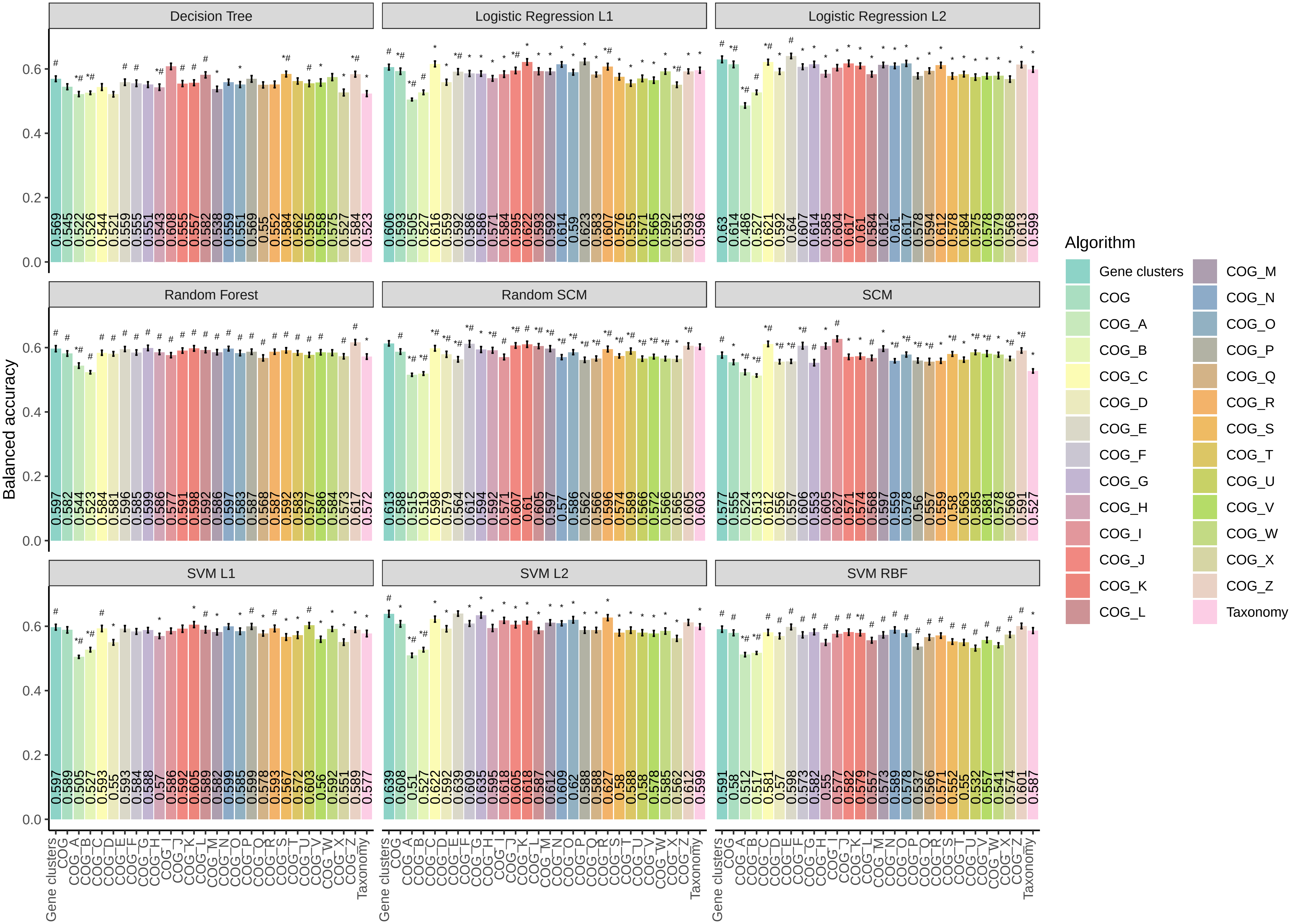

Supplementary Figure S22 − Performance of T2D classification quantified with F1 score for all algorithms

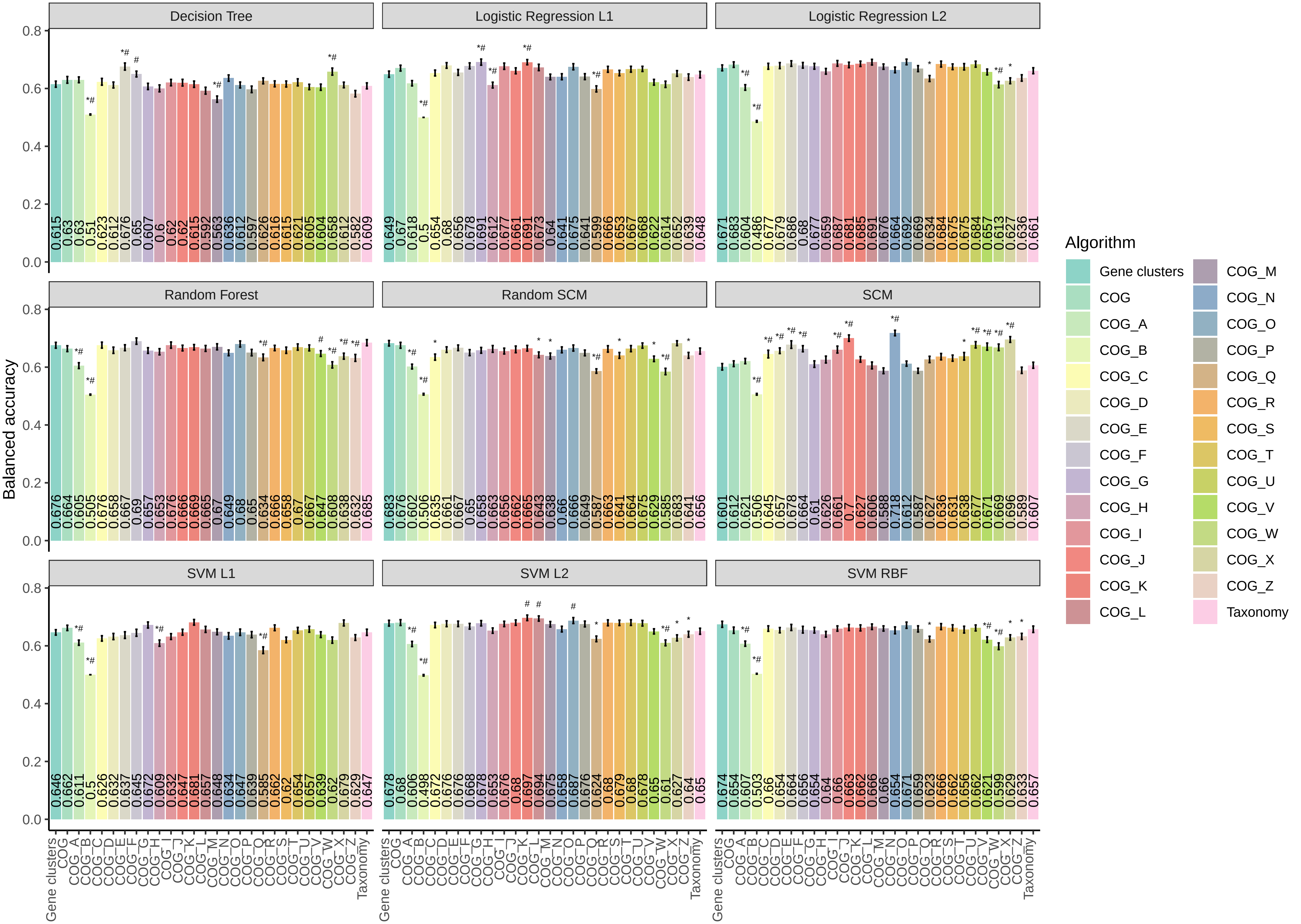

Supplementary Figure S23 − Performance of IBD classification quantified with F1 score for all algorithms

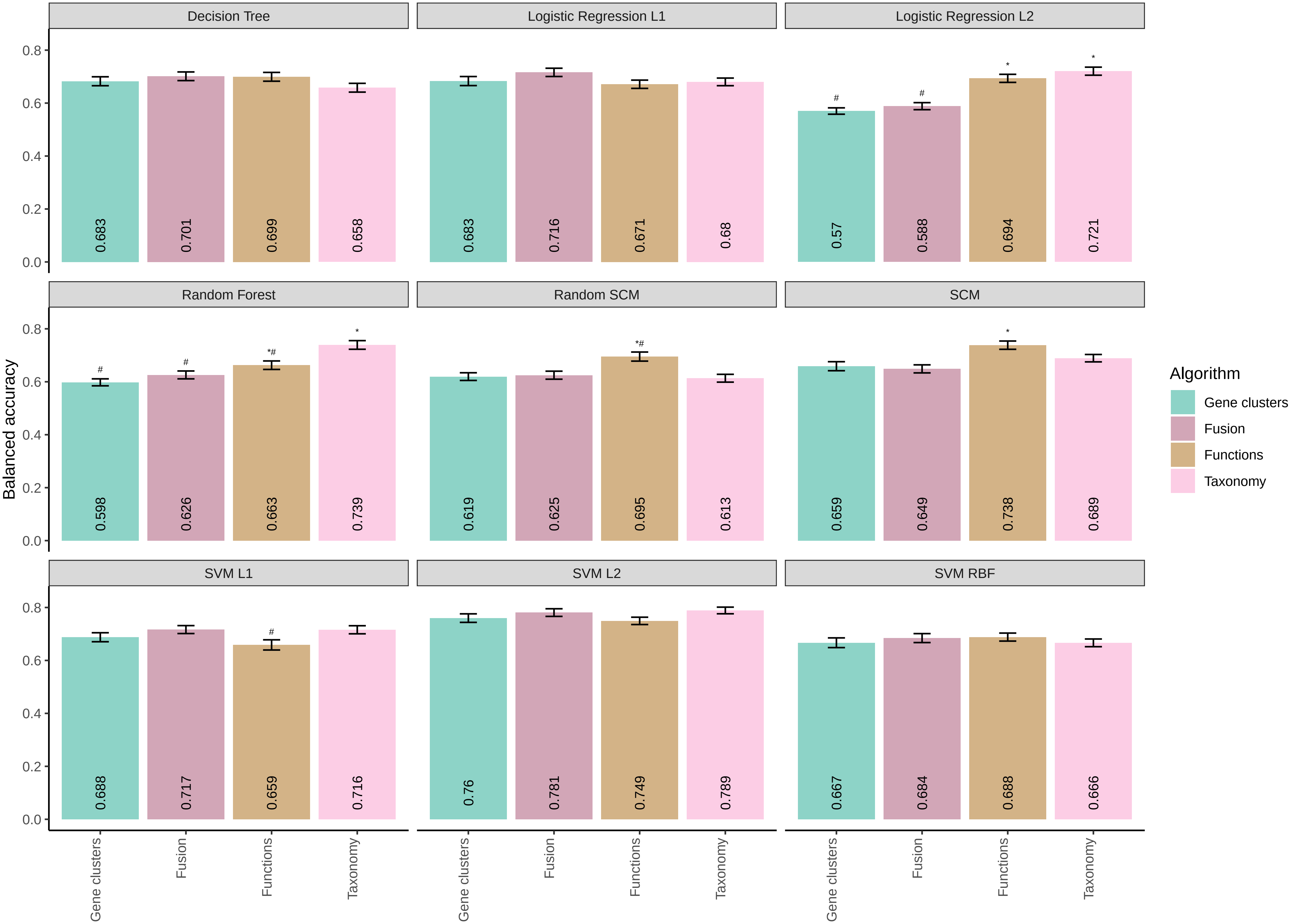

Supplementary Figure S24 − Performance of LC classification quantified with F1 score for all algorithms

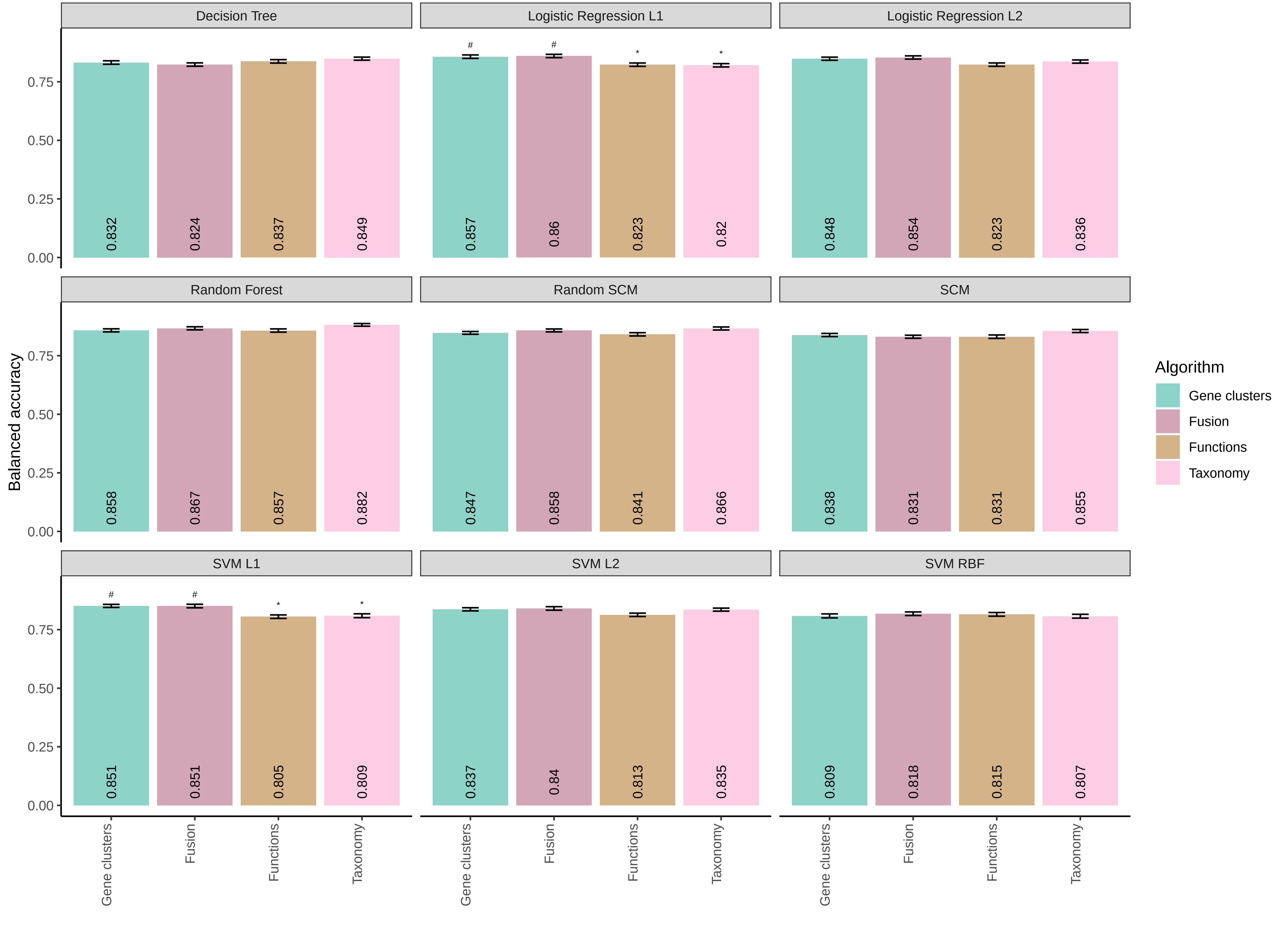

Supplementary Figure S25 − Performance of CRC classification quantified with F1 score for all algorithms

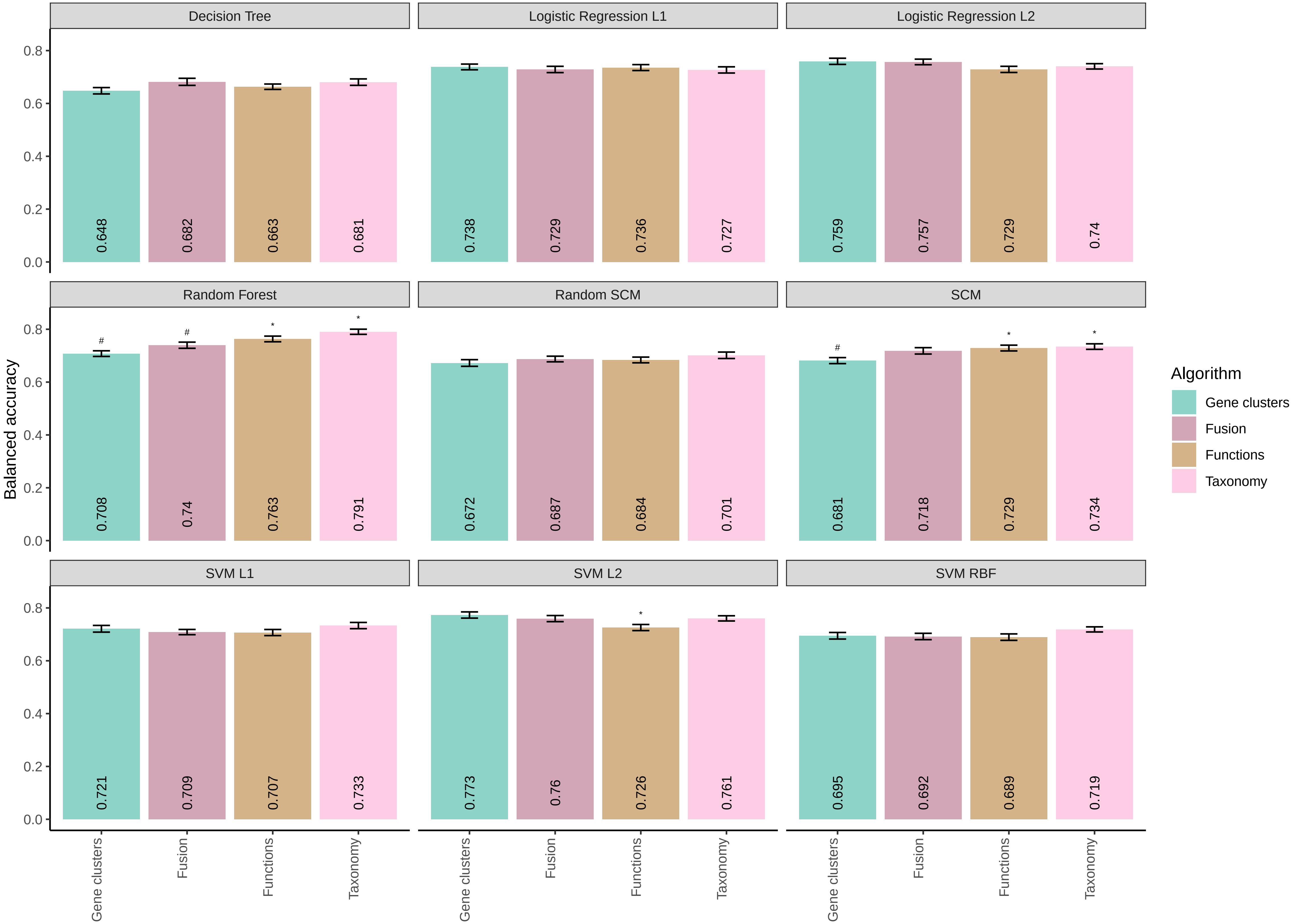

Supplementary Figure S26 − Performance of OB classification quantified with rocAUC for all algorithms

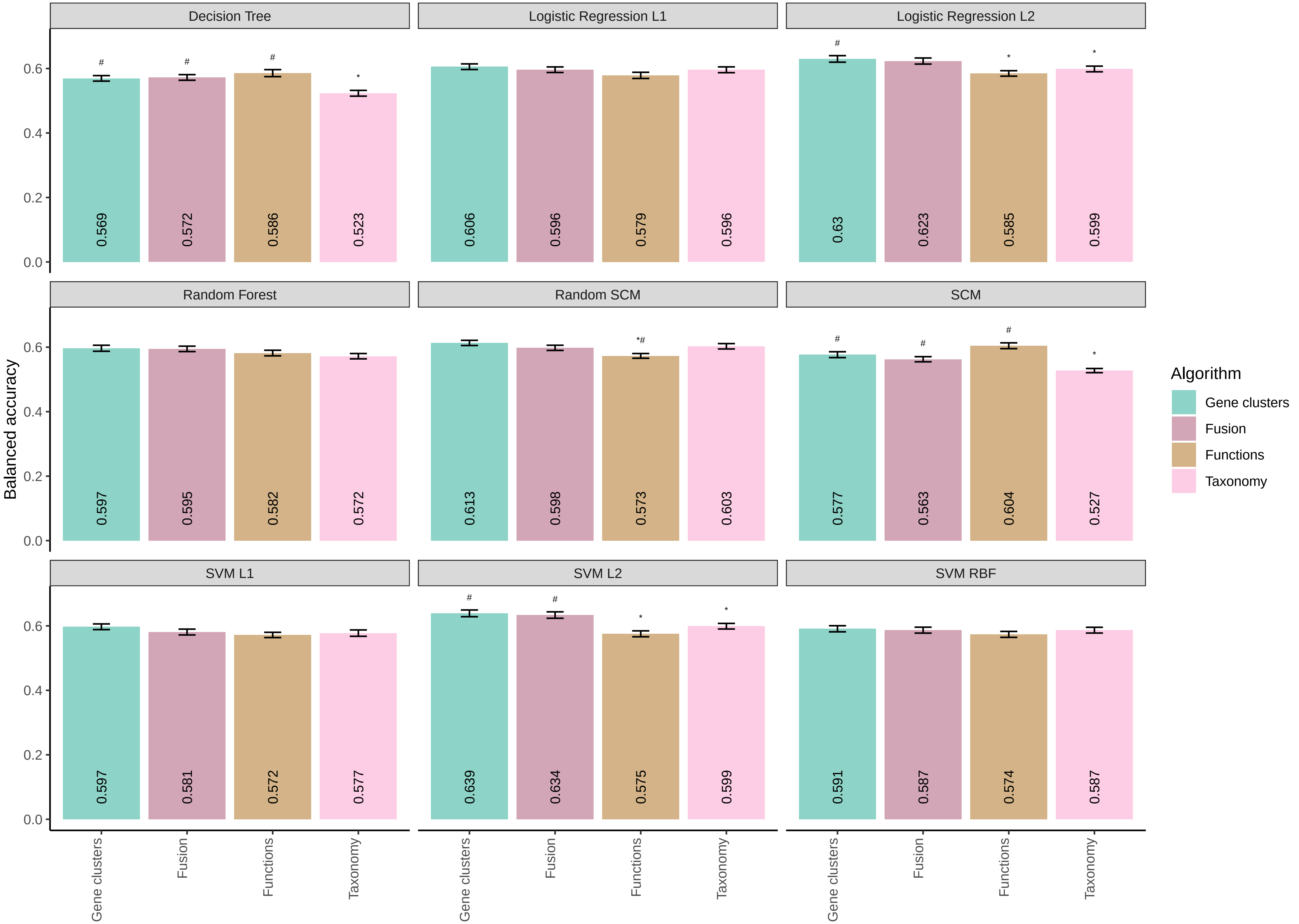

Supplementary Figure S27 − Performance of T2D classification quantified with rocAUC for all algorithms

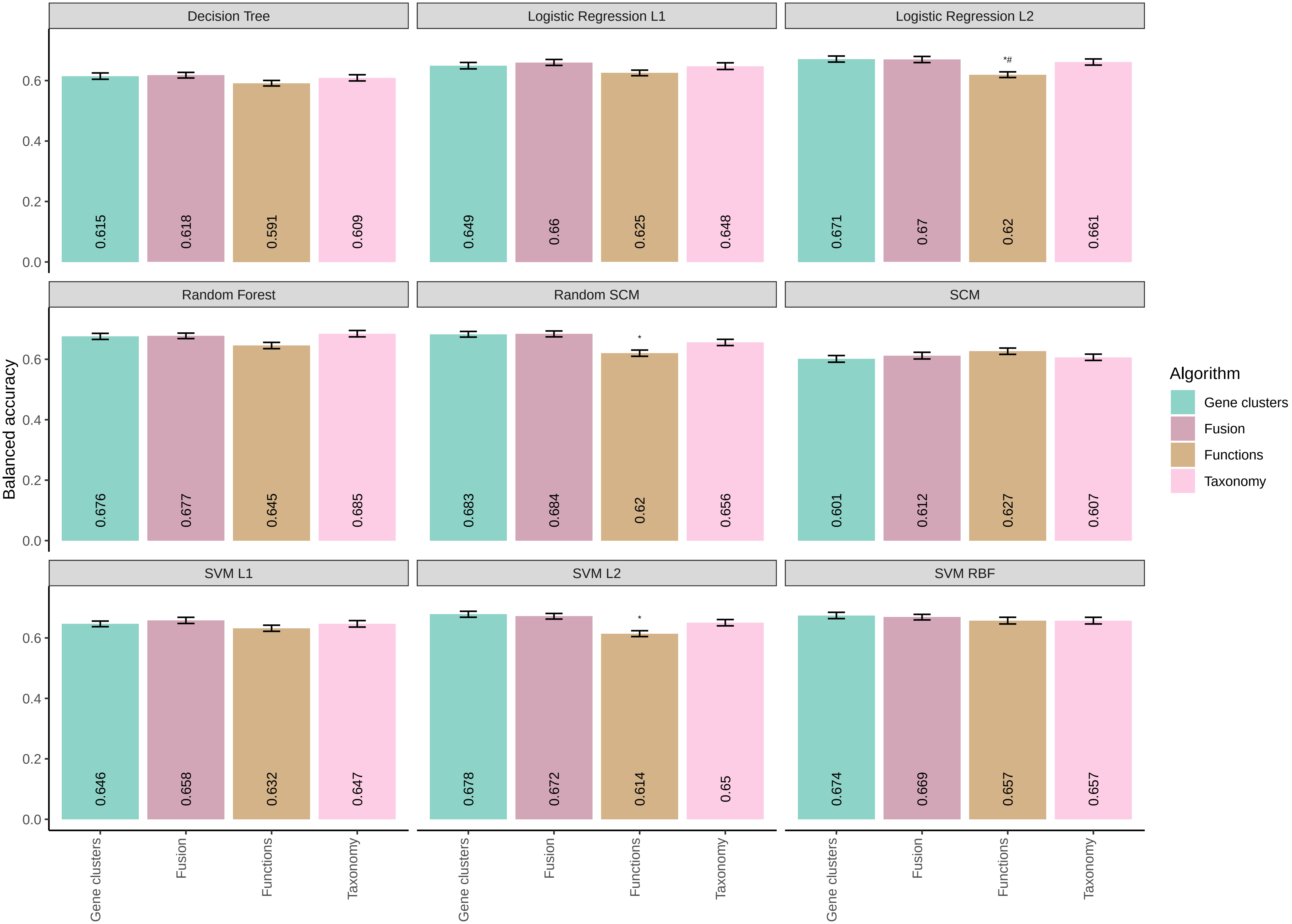

Supplementary Figure S28 − Performance of IBD classification quantified with rocAUC for all algorithms

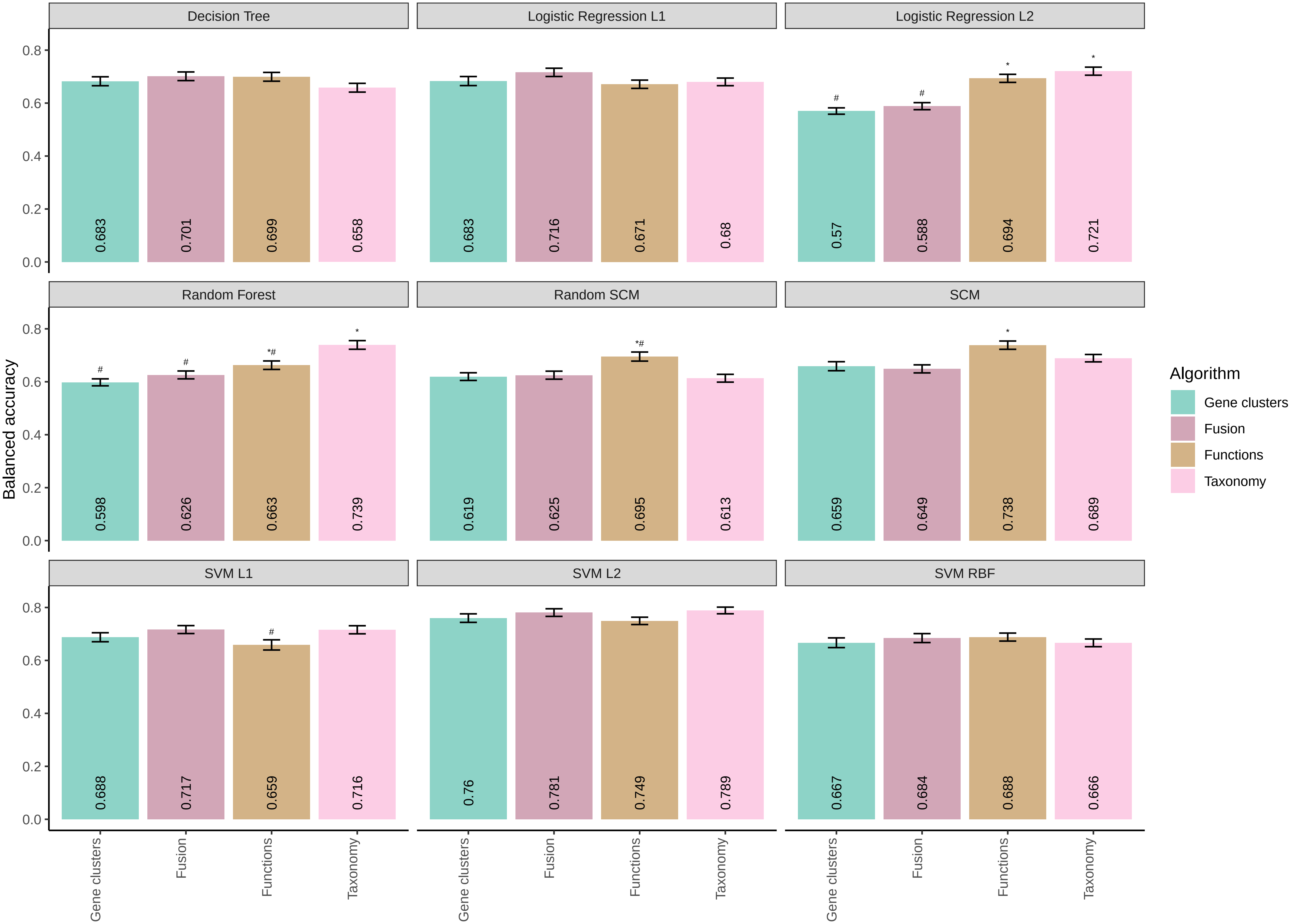

Supplementary Figure S29 − Performance of LC classification quantified with rocAUC for all algorithms

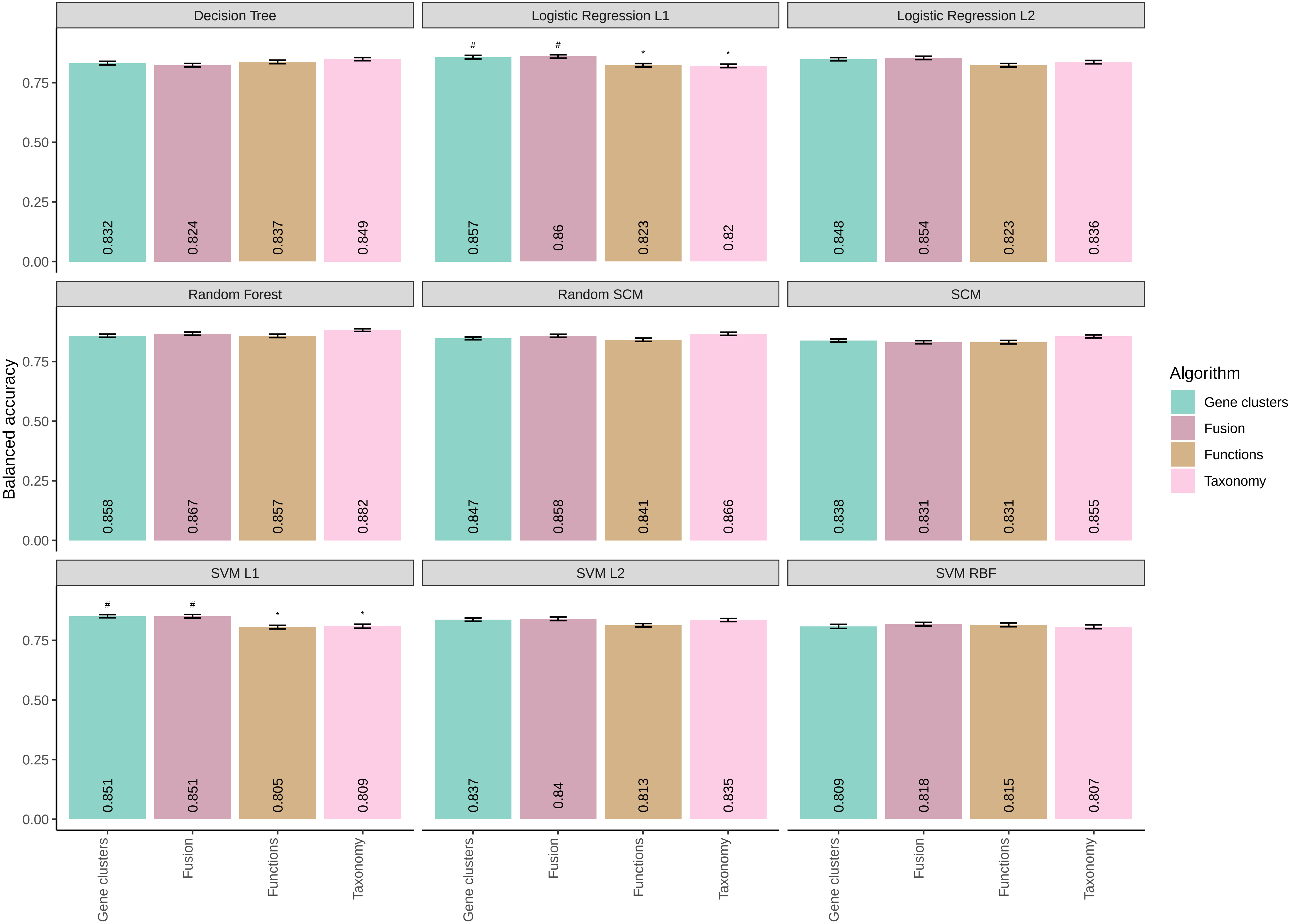

Supplementary Figure S30 − Performance of CRC classification quantified with rocAUC for all algorithms

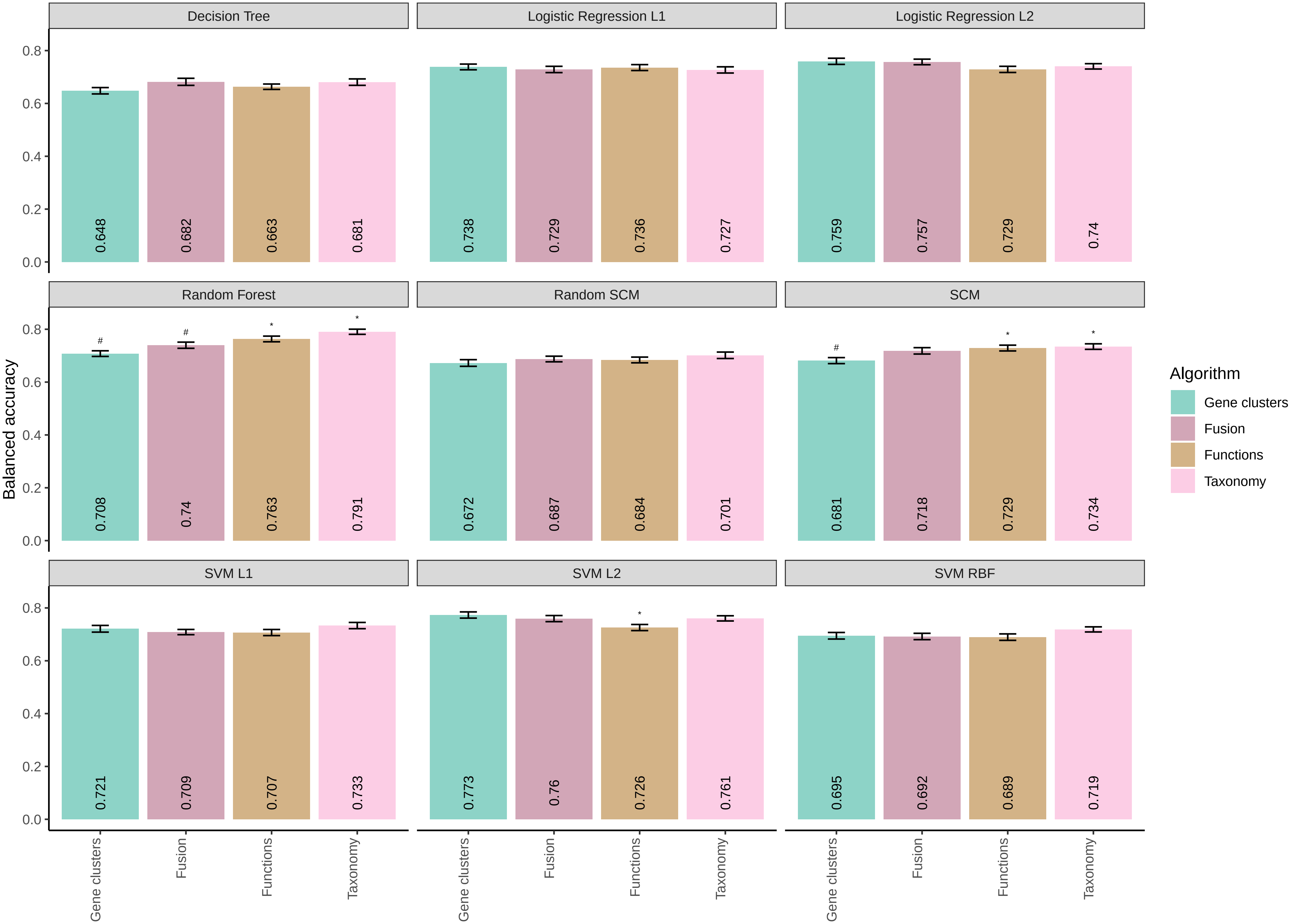

Supplementary Figure S31 − Performance of OB classification quantified with balanced accuracy for all algorithms

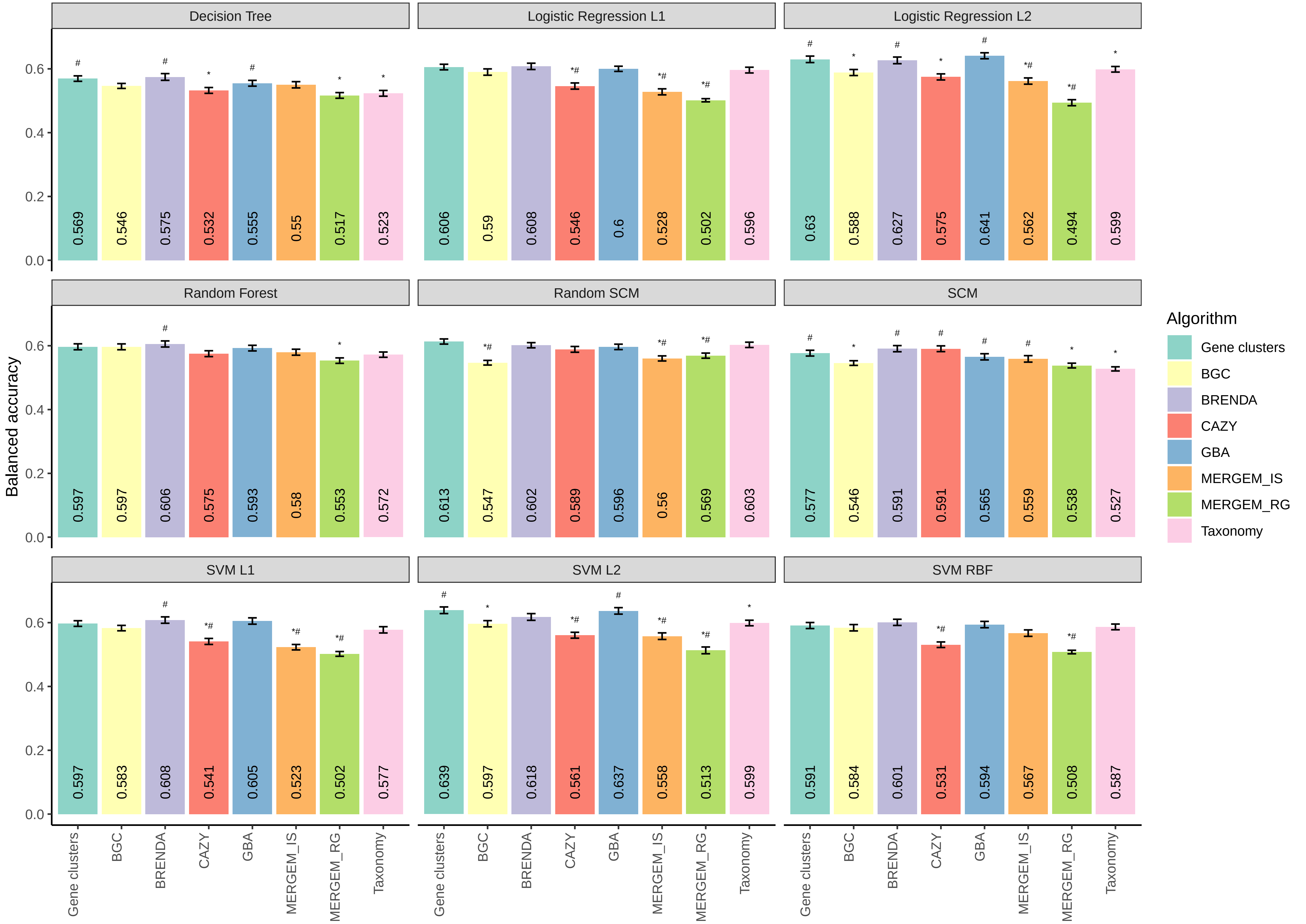

Supplementary Figure S32 − Performance of T2D classification quantified with balanced accuracy for all algorithms

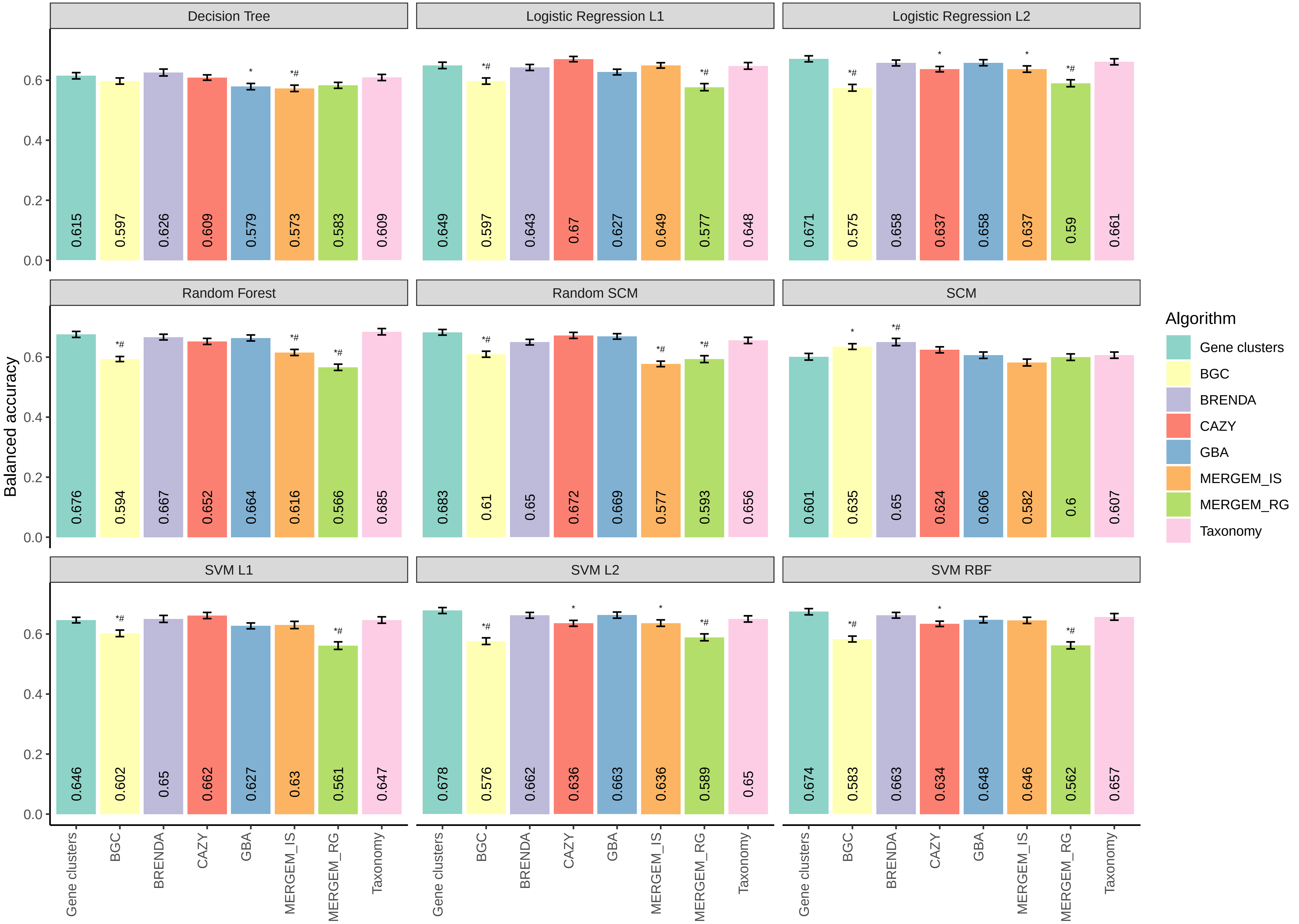

Supplementary Figure S33 − Performance of IBD classification quantified with balanced accuracy for all algorithms

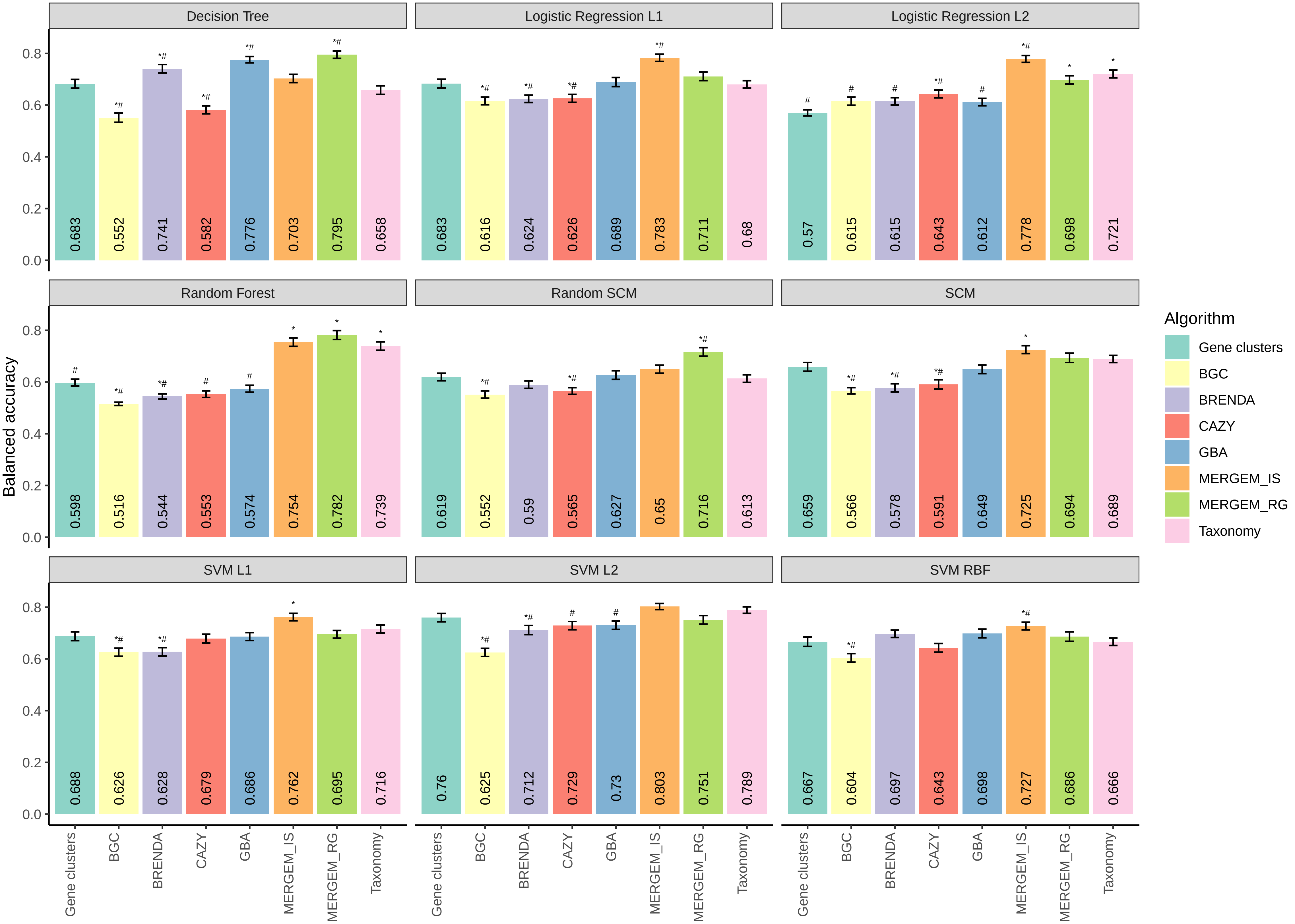

Supplementary Figure S34 − Performance of LC classification quantified with balanced accuracy for all algorithms

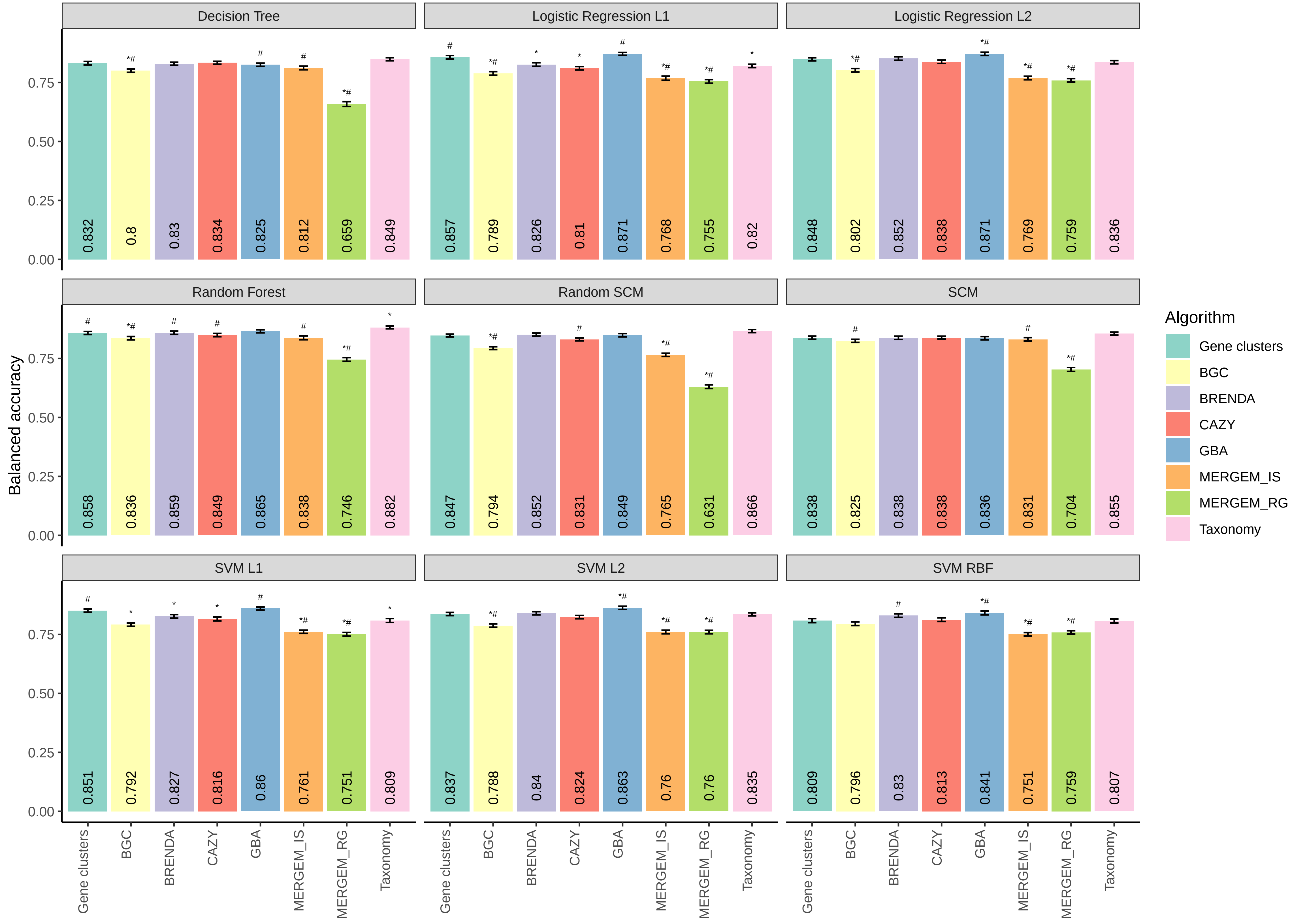

Supplementary Figure S35 − Performance of CRC classification quantified with balanced accuracy for all algorithms

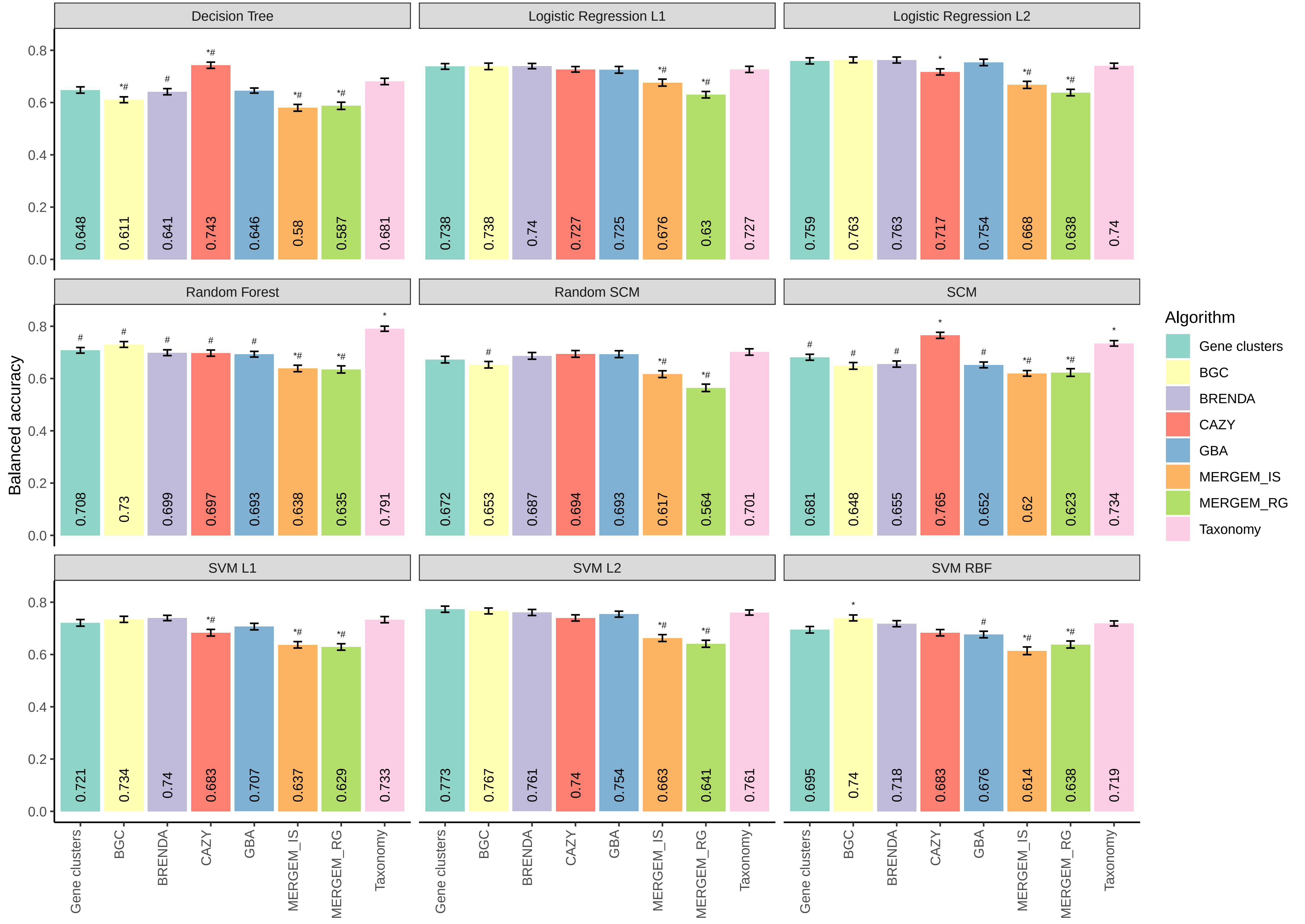

Supplementary Figure S36 − Performance of OB classification quantified with F1 score for all algorithms

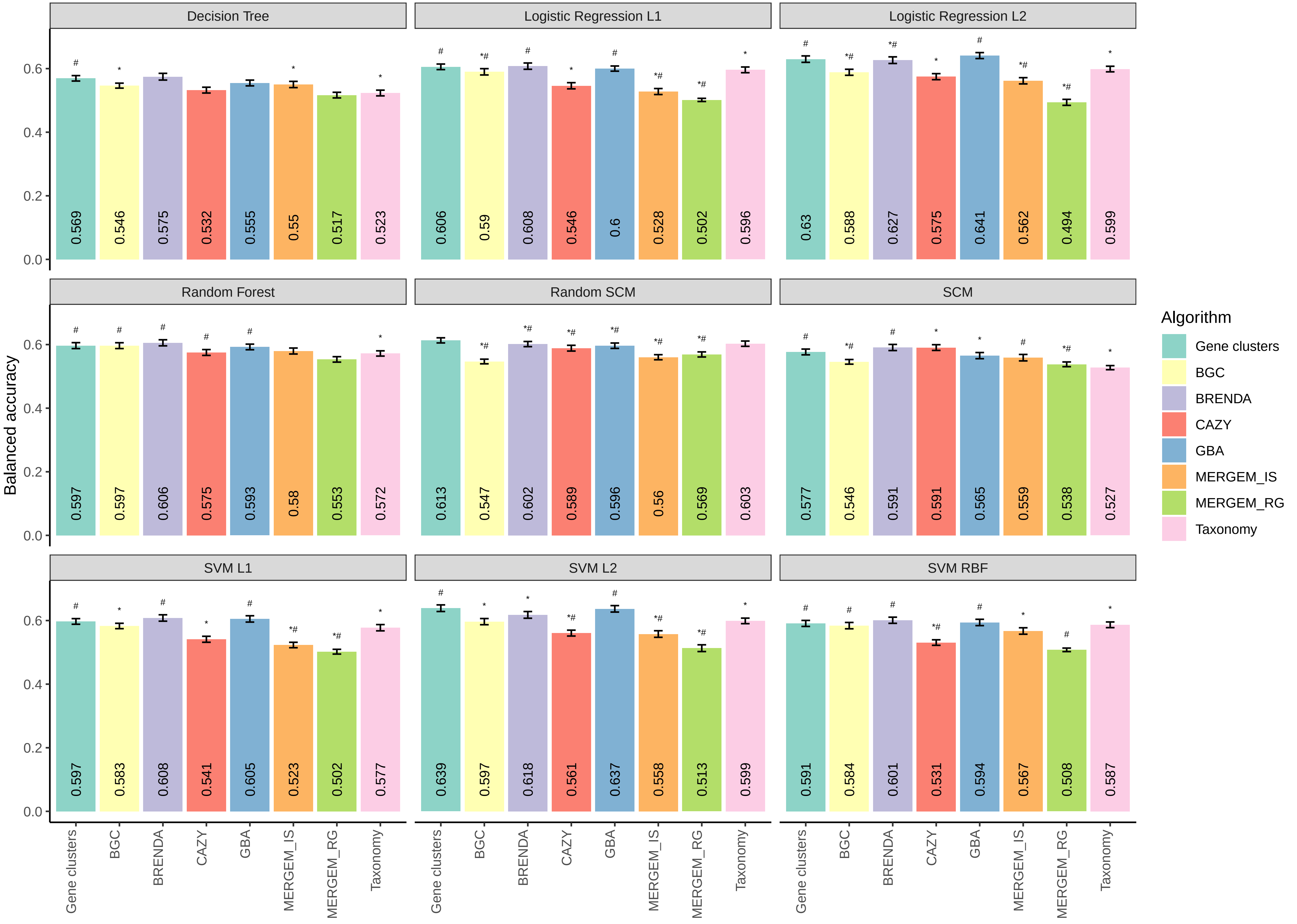

Supplementary Figure S37 − Performance of T2D classification quantified with F1 score for all algorithms

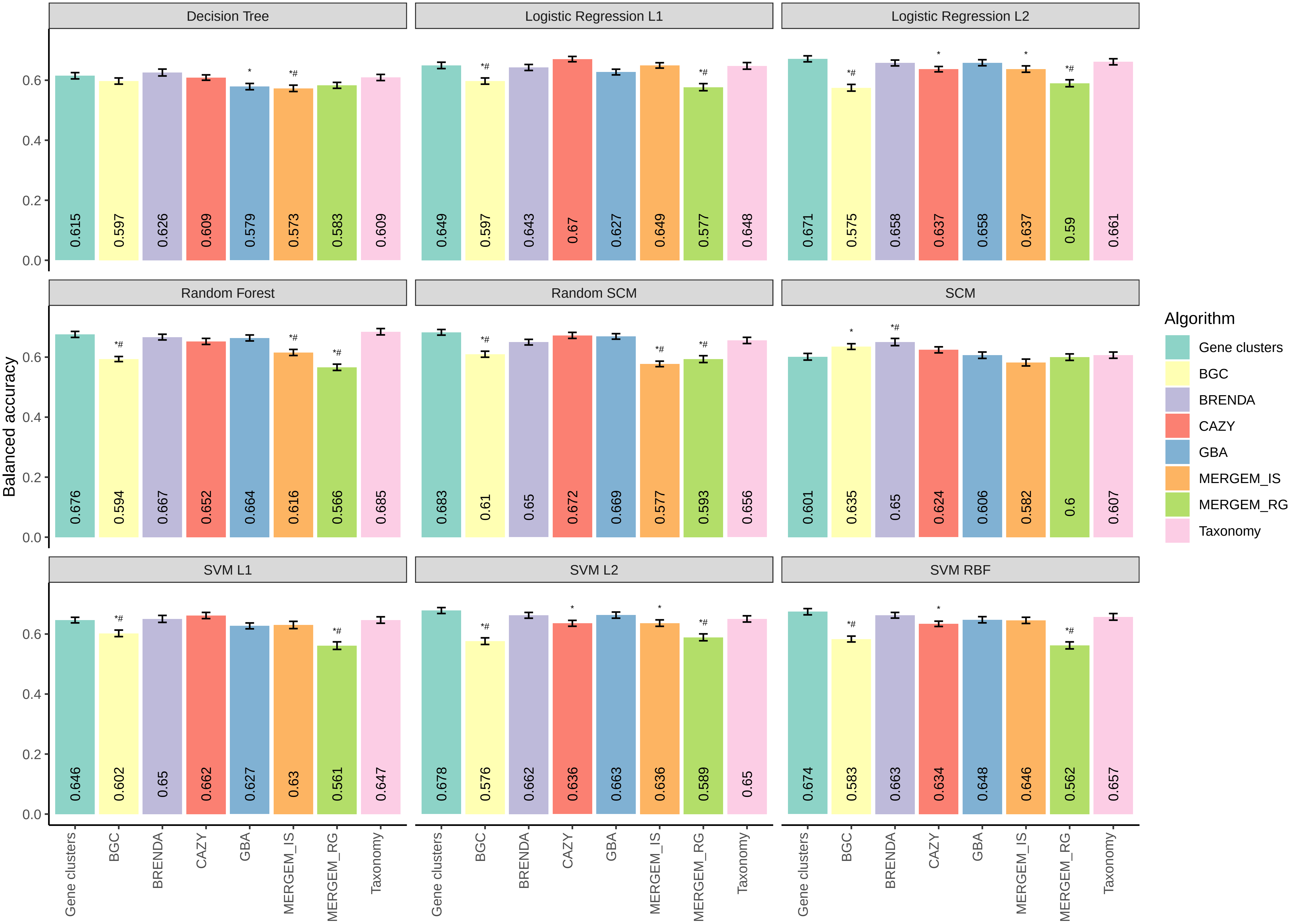

Supplementary Figure S38 − Performance of IBD classification quantified with F1 score for all algorithms

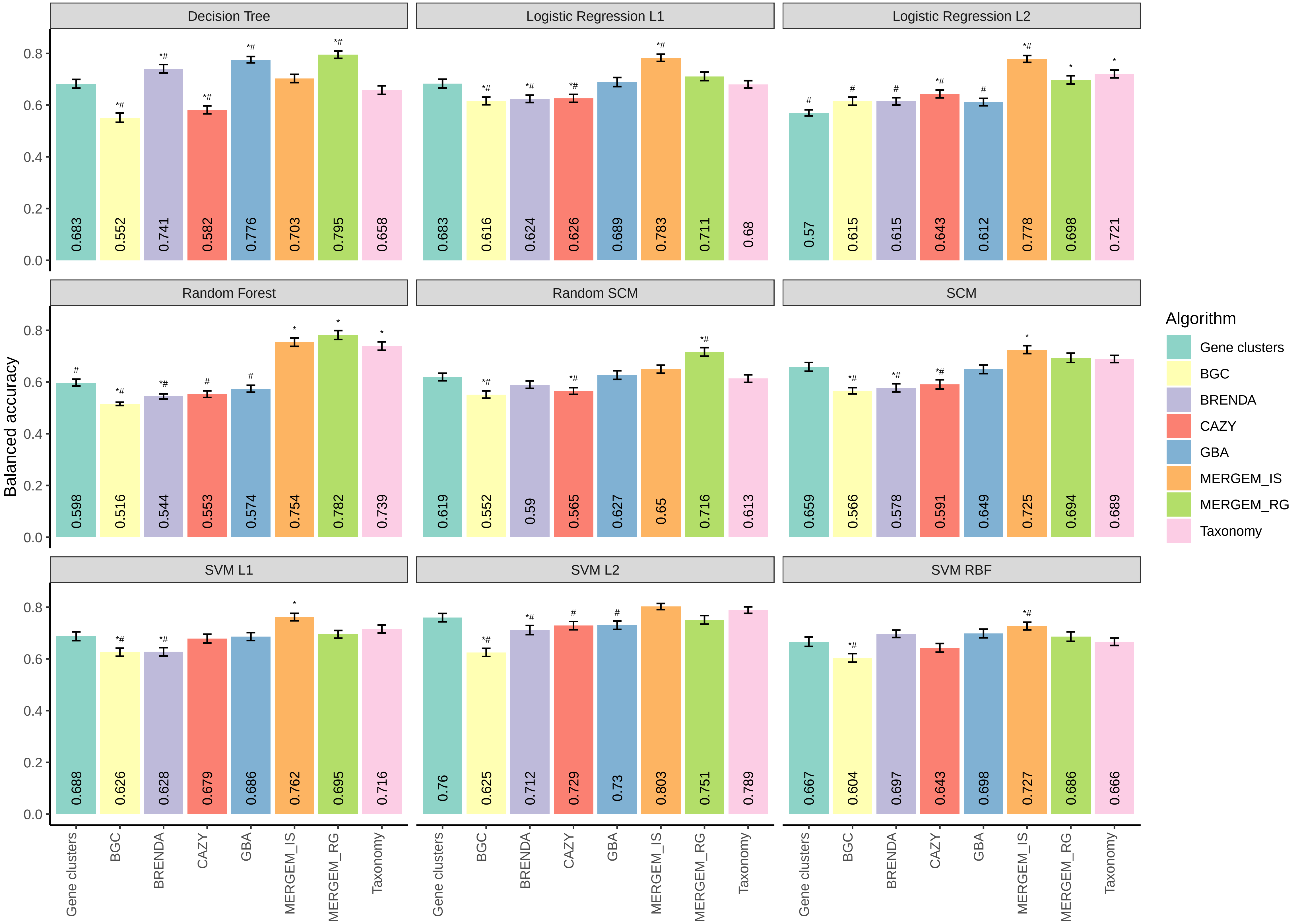

Supplementary Figure S39 − Performance of LC classification quantified with F1 score for all algorithms

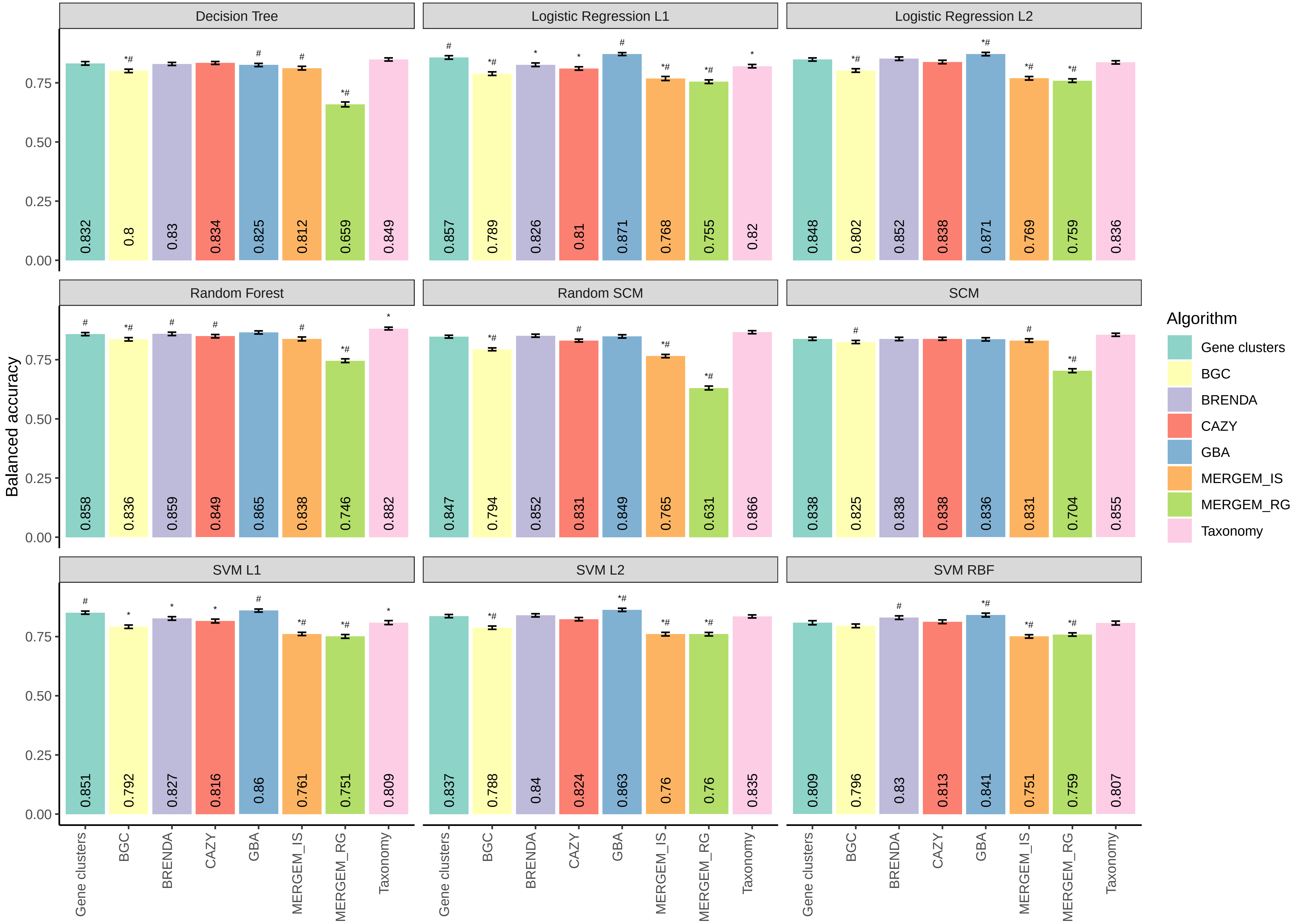

Supplementary Figure S40 − Performance of CRC classification quantified with F1 score for all algorithms

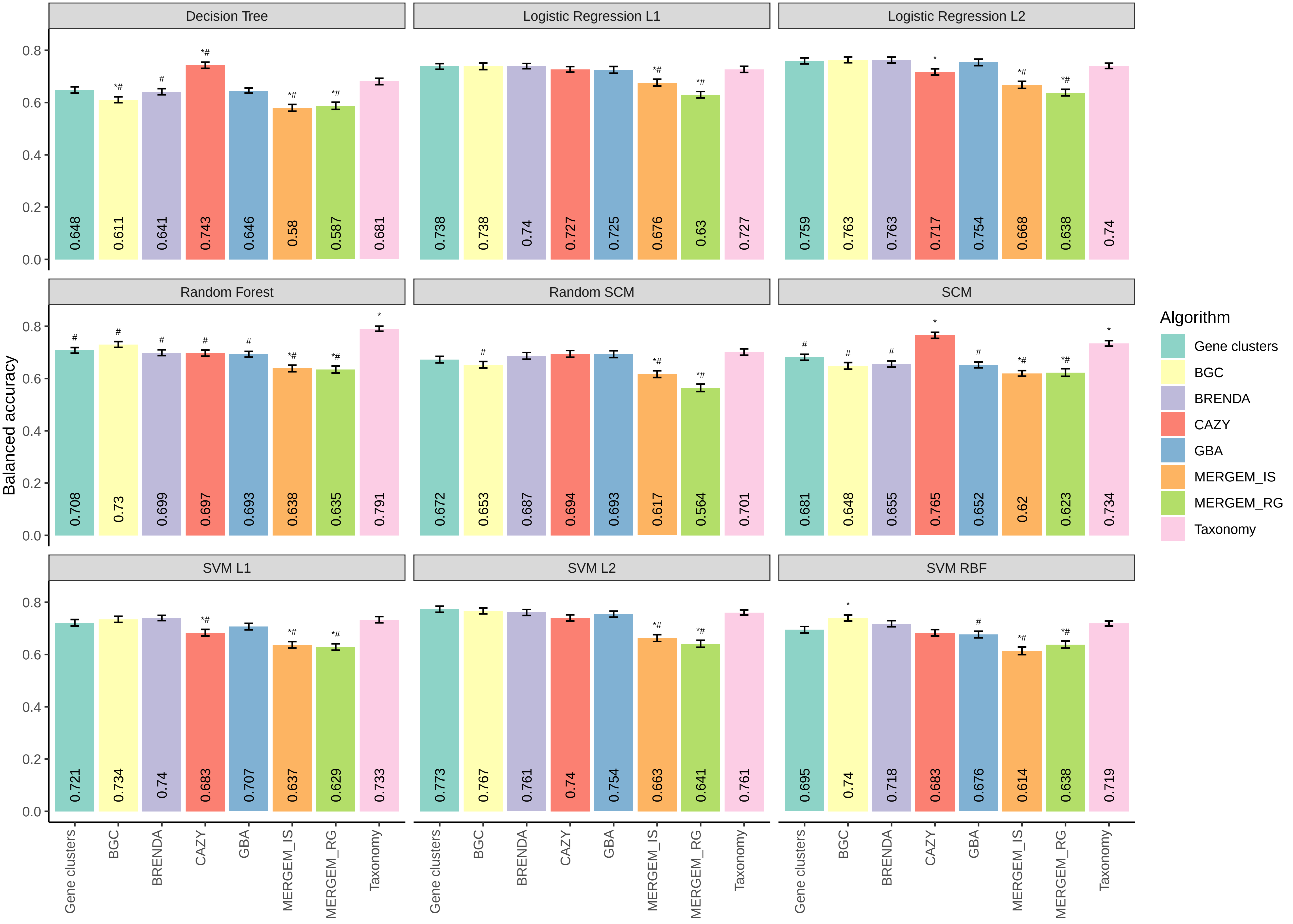

Supplementary Figure S41 − Performance of OB classification quantified with rocAUC for all algorithms

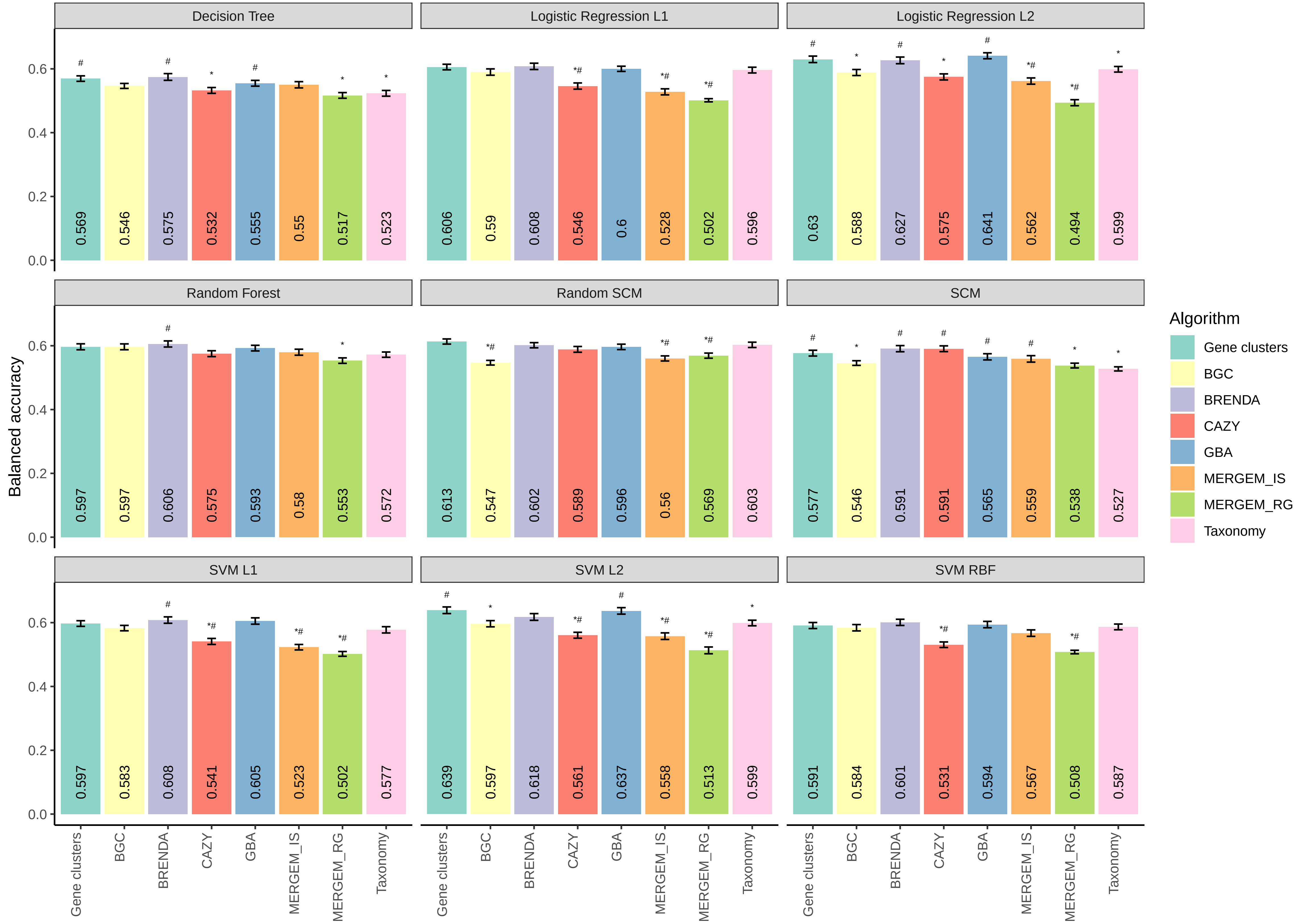

Supplementary Figure S42 − Performance of T2D classification quantified with rocAUC for all algorithms

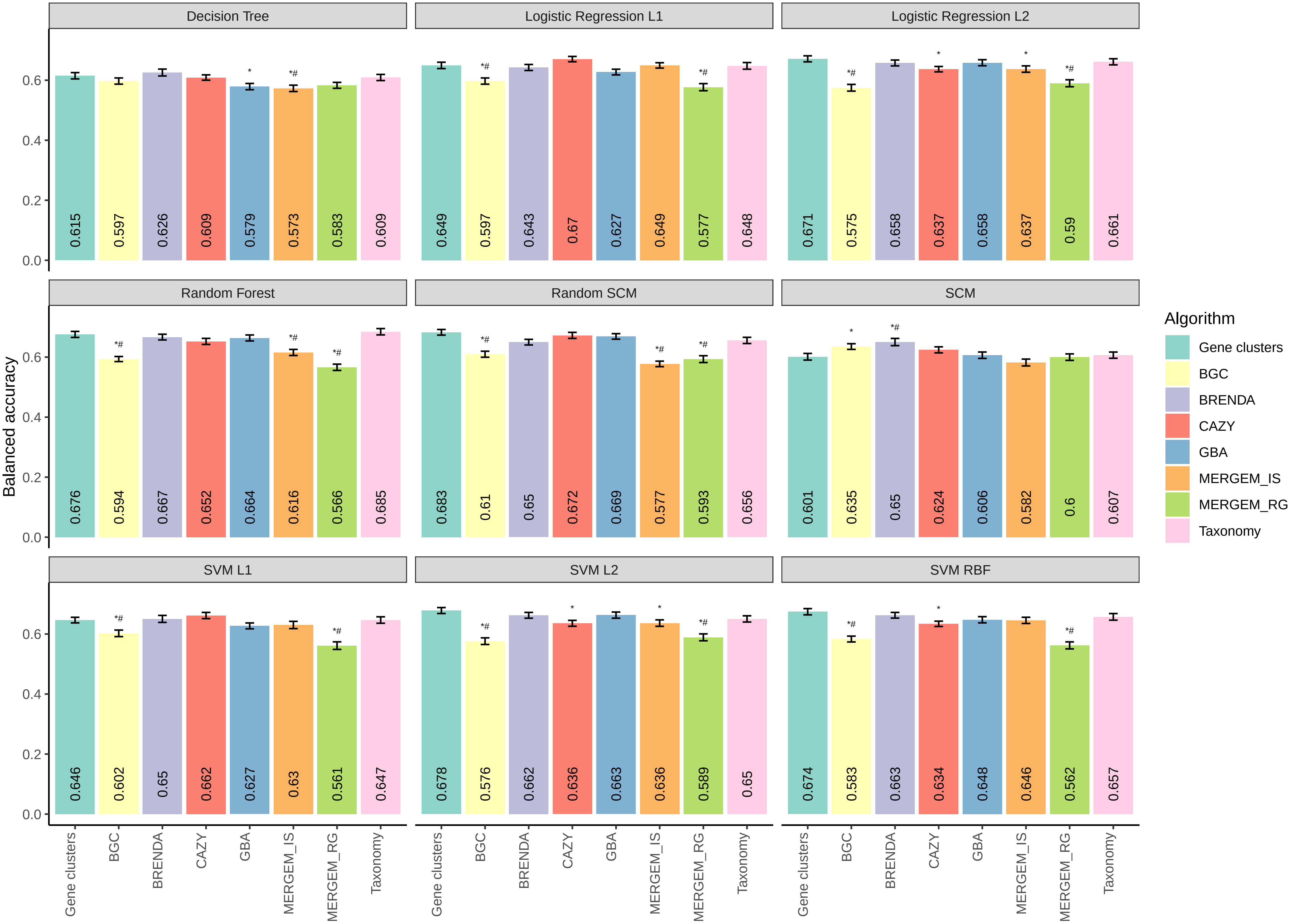

Supplementary Figure S43 − Performance of IBD classification quantified with rocAUC for all algorithms

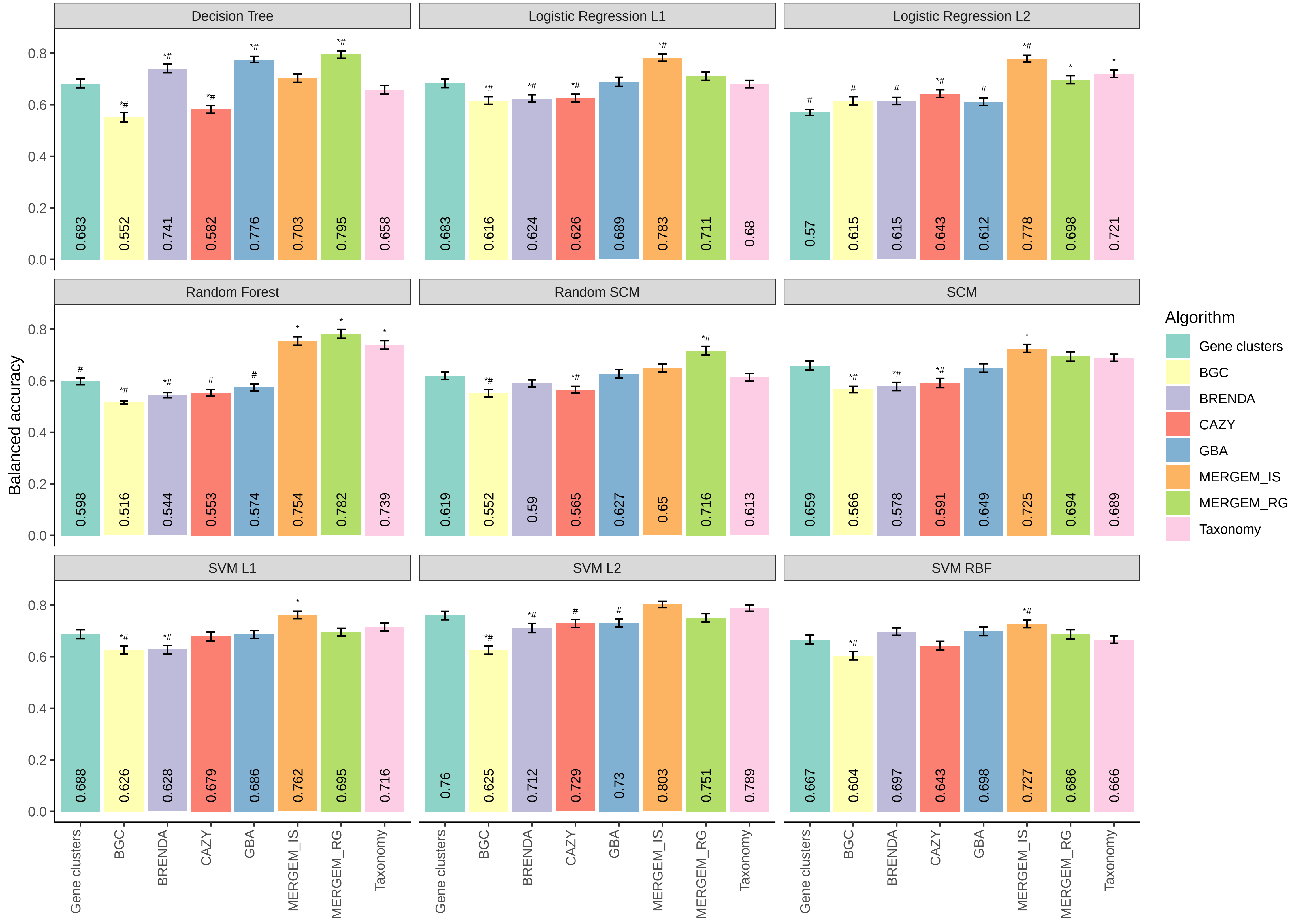

Supplementary Figure S44 − Performance of LC classification quantified with rocAUC for all algorithms

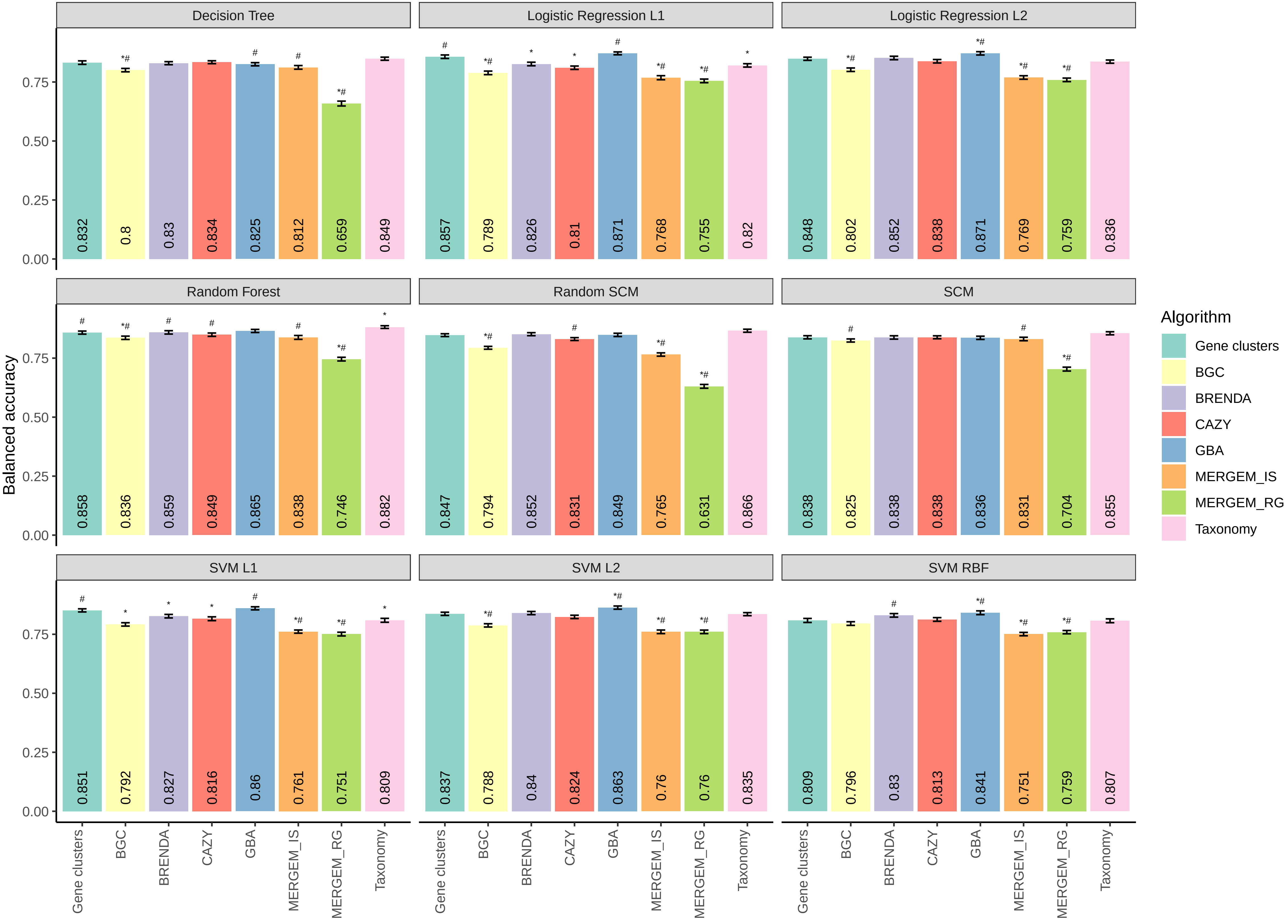

Supplementary Figure S45 − Performance of CRC classification quantified with rocAUC for all algorithms

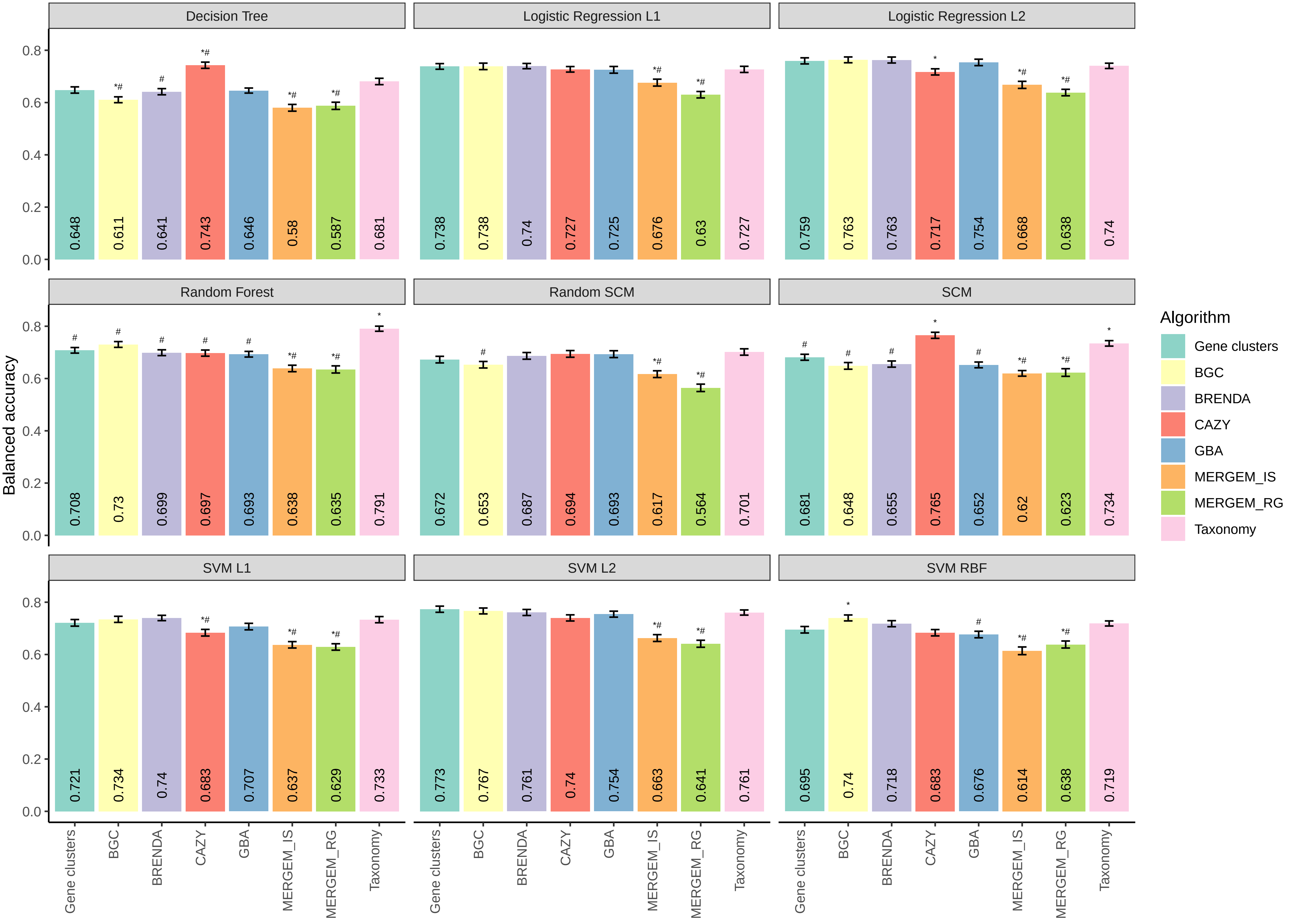

Supplementary Figure S46. Effect of CDHIT clustering parameters on classification performance. The balanced accuracies are average values for each combination of parameters and algorithms.

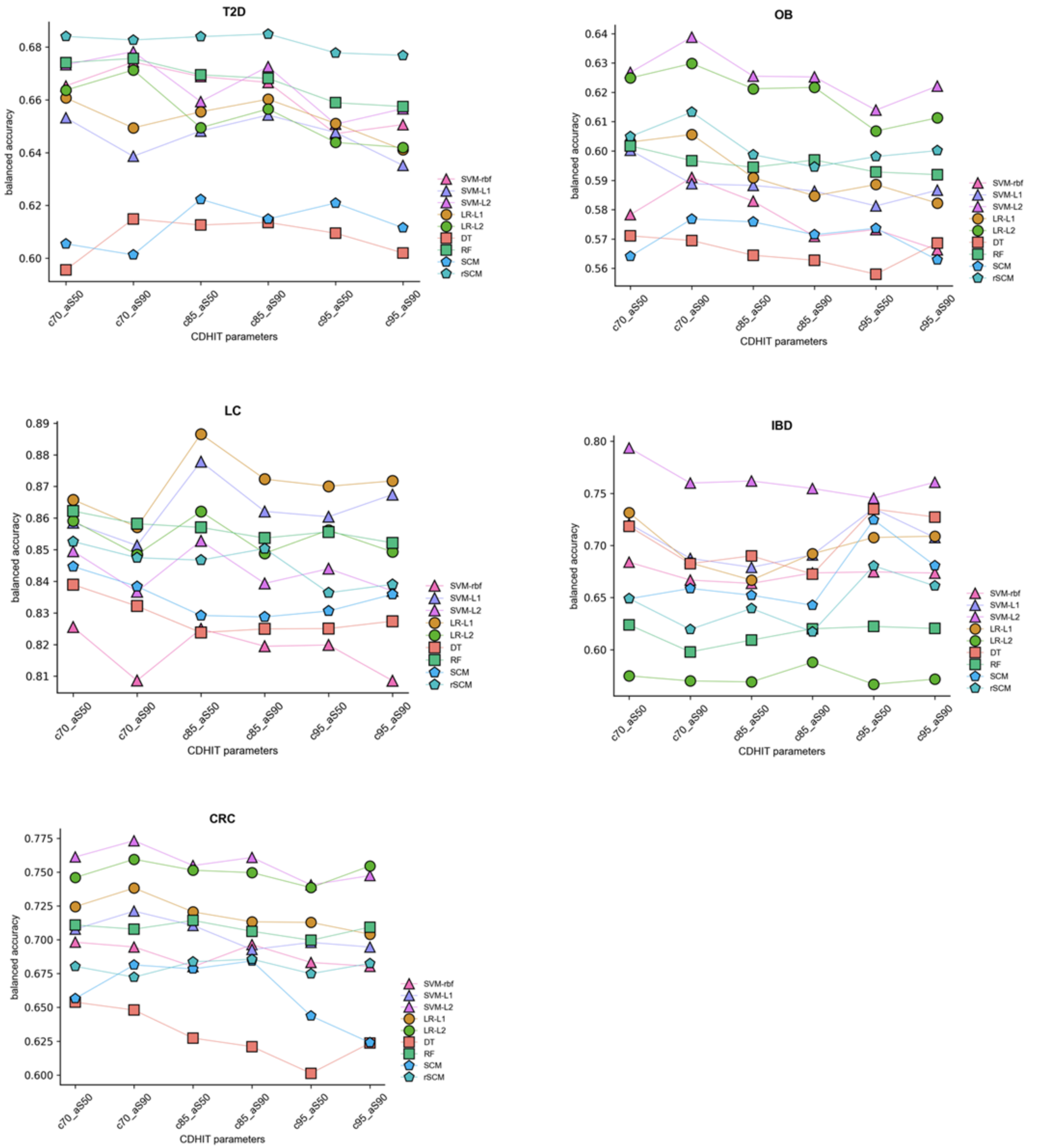

Supplement: Supplemental Figures — Figures S1 to S46. [file msystems.00531-23-s0001.pdf]
